# Blocking-cyclization technique for precise synthesis of cyclic polymers with regulated topology

**DOI:** 10.1038/s41467-018-07754-1

**Published:** 2018-12-14

**Authors:** Jie Chen, Hongfei Li, Hengchen Zhang, Xiaojuan Liao, Huijing Han, Lidong Zhang, Ruyi Sun, Meiran Xie

**Affiliations:** School of Chemistry and Molecular Engineering, East China Normal University, Shanghai, 200241 China

## Abstract

Ring-closure and ring-expansion techniques are the two routes for extensive synthesis of cyclic polymers. Here, we report an alternative blocking-cyclization technique referred to as the third route to prepare cyclic polymers with regulated ring size and ring number by ring-opening metathesis polymerization of di- and monofunctional monomers in a one-pot process, where the polymer intermediates bearing two single-stranded blocks are efficiently cyclized by the cyclizing unit of propagated ladderphane to generate corresponding mono-, bis-, and tricyclic polymers, and the well-defined ladderphane structure plays a crucial role in forming the cyclic topology. Monocyclic polymer is further modified via Alder-ene reaction and the cyclic molecular topology is clearly demonstrated. The diversity features of cyclic polymers are comprehensively revealed. This strategy has broken through the limitations of previous two cyclizing routes, and indeed opens a facile and popular way to various cyclic polymers by commercial Grubbs catalyst and conventional metathesis polymerization.

## Abstract (web summary)

Cyclic polymers can be usually accessed by ring-expansion and ring-closure approaches. Here, the authors report an alternative blocking-cyclization technique which enables the tunable synthesis of multicyclic polymers via commercial Ru-based Grubbs catalysts and conventional ring-opening metathesis polymerisation.

## Introduction

**Fig. 1:**
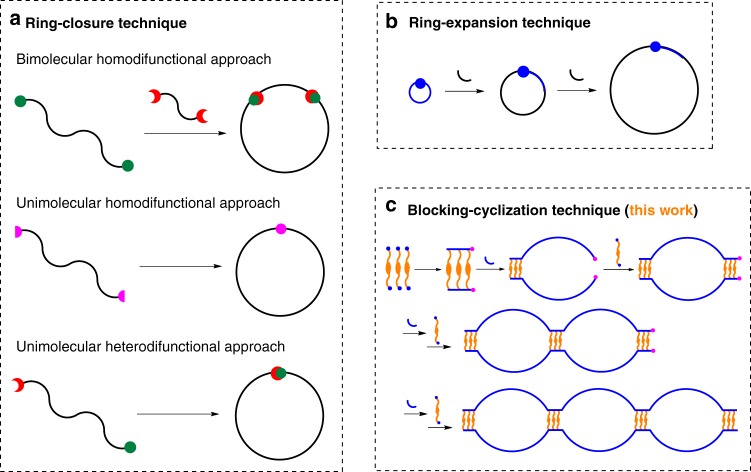
Schematic representation of different techniques for the preparation of cyclic polymers. **a** Ring-closure. **b** Ring-expansion. **c** Blocking-cyclization

In the past decades, cyclic polymers have attracted a large academic attention because of their unique physical and solution properties intrinsic to their endless molecular topology^1–4^, including a more significant difference in the surface structure^5,6^, smaller hydrodynamic volume^7,8^, lower viscosity^9,10^, and higher glass transition temperature^11^ in comparison to the usual linear analogues. Despite there have a large number of reports on the formation of cyclic polymers previously, the strict synthetic strategies were divided into only two categories: ring-closure technique and ring-expansion technique^10,12,13^. The ring-closure technique (Fig. 1a) is a traditional route, containing separated two steps of linear polymer precursor synthesis and its cyclization at extremely low concentration of 10^−5^ mol L^−1^ ^6,12,14,15^. Initial synthetic pathway is the one way focused primarily on the bimolecular homodifunctional cyclization, due to the relative easy synthesis of homodifunctional polymer precursors. However, if one of the homodifunctional polymers is excess or insufficient, both end-groups of the identical polymer will react with another homodifunctional polymer to produce linear by-product and thus prevent cyclization. Even with 1:1 stoichiometry, excluding this reaction still be difficult because of the detailed competitive kinetics between intramolecular cyclization and intermolecular oligomerization^12^. In order to overcome the complication of bimolecular homodifunctional polymer cyclization, an alternative approach that the direct coupling reaction between two end-groups of unimolecular homodifunctional polymer precursor has been extended^16,17^. For example, the linear precursor with telechelic allyl end-groups was easily converted to macrocycle by a Grubbs catalyst-mediated ring-closing metathesis at extremely low concentration^17^. The other known way is the cyclization of unimolecular heterodifunctional polymer precursors bearing highly active functional groups at opposite chain ends by efficient coupling reaction at high dilution^18,19^, resulting in a high purity cyclic polymer. The most common one is the click cyclization of linear *α*,*ω*-heterodifunctional polymer precursor bearing azido and ethynyl groups to form the 1,4-disubstituted 1,2,3-triazole-connected cyclic polymer^4,15,16,18,20^. This method requires a very low molarity of reactive end groups for the cyclization reaction at high dilution, while it is still highly challenging to synthesize the well-defined cyclic polymers with large rings by this method, because the cyclization reaction of long linear precursors is thermodynamically disfavored, and the entropic penalty associated with localizing two end-groups of identical polymer chain into a region of required space for cyclization increases exponentially with the chain length. Instead of small functional groups at chain-ends, the cyclization of the ABC triblock copolymer precursors, composed of long central block B and two short blocks A and C bearing reactive antagonist functions under highly dilute conditions, is an alternative pathway to cyclic polymers^5,6^. In a word, the linear polymer precursor and highly diluted reaction condition are inevitably required in ring-closure technique, and the cyclization always occurs on the two reactive end groups of isolated polymer precursor without living species and must be triggered by the extra initiator.

Another synthetic route is ring-expansion technique (Fig. 1b), and the in situ cyclization invariably arises from the cyclic living species, in which two types of cyclic catalysts play a crucial role. The one is the zwitterionic *N*-heterocyclic carbene^8,21,22^ or cyclic tin dialkoxide^23–26^ for initiating ring-opening polymerization of lactone or carbosiloxane to cyclic polymers with controlled number-average molecular weight (*M*_n_), narrow polydispersity index (PDI), and various topological structures; the other is cyclic Ru-based metathesis catalyst^2,3,9^ for initiating ring-expansion metathesis polymerization (REMP) of cyclic olefins to cyclic polyolefins, where monomer insertion into a reactive or weak bond expands the cycle and avoids the formation of linear coproducts^12,13^, and the cyclic polymer can either retain the propagating species or remove the catalysts by a back-biting or intramolecular chain transfer reaction in these cases^12^. REMP opens undoubtedly an attractive way to directly achieving cyclic polymers with high *M*_n_, narrow PDI, and high purity in a normal reactant concentration (>0.1 mol L^−1^). Nonetheless, the formidable synthesis challenge of cyclic catalyst make this approach hard to be popularized. Therefore, it is envisioned for designing a facile synthetic route to cyclic polymers by the commercial Grubbs catalyst.

As a class of double-stranded polymers, ladderphane structure^27–30^, constituted of multiple layers of rigid linkers connected to two polymeric backbones, has two independent propagating carbene ends in ring-opening metathesis polymerization (ROMP) process, which can further initiate the monofunctional monomers to form the AB_2_-type block copolymer, where ladderphane block A is a double-stranded segment and block B is a single-stranded segment^31–33^. Because polynorbornene (PNBE) chain with N-arylpyrrolidine pendants adopted a rigid rod-like structure^34,35^, the two B blocks attracted by the π–π stacking effect of pendants, resulting in that the two end groups of B blocks are in close proximity to each other.

Herein, we demonstrate that if the monofunctional monomer is completely reacted and the two propagating carbene ends of block B are maintained living feature in the process, when the difunctional monomer is added again into the living reaction system, a new ladderphane A was formed and served as the blocking unit to cyclize the linear AB_2_ copolymer intermediate. As a result, the monocyclic block copolymer AB_2_A could be generated in such sequential addition of di- and monofunctional monomers, and this strategy could be defined as the blocking-cyclization technique (Fig. 1c). Interestingly, when the di- and monofunctional monomers are alternately added one by one into the living reaction system, the multicyclic block copolymers A(B_2_A)_*x*_ are readily achieved in a controlled one-pot process. This blocking-cyclization technique is different from the previous two cyclization techniques, and possesses some distinguishing features: (i) cyclization is realized by conventional ROMP, and the used Grubbs catalyst is commercially available, (ii) using ladderphane as the cyclizing unit to ensure the efficient cyclization and high purity cyclic structure, (iii) a relatively high monomer concentration is permitted (initial concentration > 10^−4^ mol L^−1^, and the total concentration up to 0.2–1.6 × 10^−2^ mol L^−1^), (iv) without the separation and purification treatments of linear polymer precursor, and (v) the ring size and ring number are regulated by a simple process. In brief, the prominent feature of this technique is that the cyclization multiply happens in situ on the two ends of polymer intermediate containing living species, and the propagating ladderphane is simultaneously served as the cyclizing unit. Therefore, the developed blocking-cyclization strategy could be considered as the third route and a versatile platform to cyclic polymers by commercial Grubbs catalyst and conventional ROMP.

## Results

### Blocking-cyclization technique

**Fig. 2:**
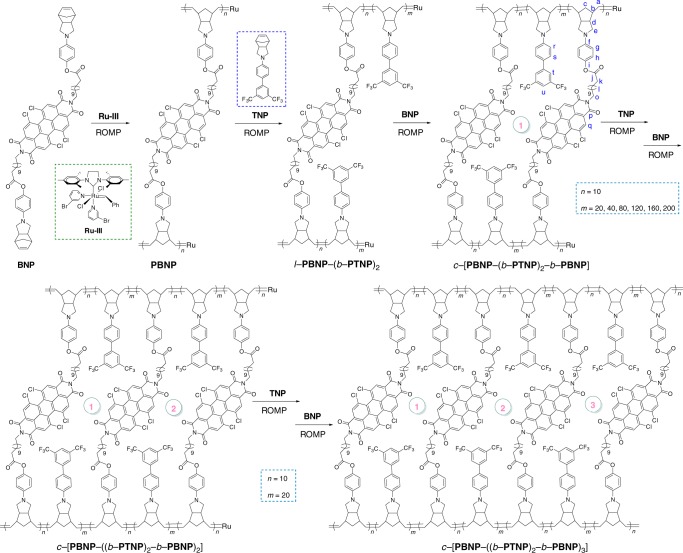
Blocking-cyclization process. Syntheses of mono-, bis-, and tricyclic polymers by successive ROMP

As a macrocyclization strategy, the effectiveness of blocking-cyclization technique for synthesizing cyclic polymer should be evaluated systematically. It is astonishing to find that this technique possessed a versatility for preparing monocyclic *c*-[**PBNP**-(*b*-**PTNP**)_2_-*b*-**PBNP**] and multicyclic *c*-[**PBNP**-((*b*-**PTNP**)_2_-*b*-**PBNP**)_*x*_] polymers by the third-generation Grubbs catalyst (**Ru-III**)-mediated successive ROMP of di- and monofunctional norbornene (NBE) derivatives, bis(norbornene pyrrolidine phenyl perylene bisimide) (**BNP**) containing the rigid perylene bisimide (PBI) linker and N-3,5-bis(trifluoromethyl)biphenyl-norbornene pyrrolidine (**TNP**), as depicted in Fig. 2 and Supplementary Fig. 1. The experimental details are described in Supplementary Methods.

### Synthesis and characterization of monocyclic polymers

As an initial attempt, the linear block copolymer *l*-**PBNP**_40_-(*b*-**PTNP**_20_)_2_ with short chain length and its cyclic counterpart *c*-[**PBNP**_20_-(*b*-**PTNP**_20_)_2_-*b*-**PBNP**_20_] were prepared by ROMP of difunctional **BNP** and monofunctional **TNP** with the feed ratios ([**BNP**]:[**TNP**]:[**BNP**]:[Cat]) of 20:20:0:1 and 10:20:10:1 (entries 1 and 2, Table 1), respectively. According to the feed ratio, the theoretical block ratio of **PBNP/PTNP** should be 40:40. By integrating the peak signals related to the aromatic protons (H_q_) on PBI linker at 8.72–8.54 ppm and those on bis(trifluoromethyl)biphenyl (H_s+t+u_) at 8.09–7.34 ppm in ^1^H NMR spectra (Supplementary Fig. 2a, b), as expected, the actual **PBNP**/**PTNP** ratios of linear and cyclic polymers were separately 40:41.3 and 40:44.8, which were similar to each other and almost consistent with the theoretical block ratios, indicating that the polymerization was well-controllable and the chain-transfer reaction in **Ru-III**-mediated ROMP of NBE derivatives rarely occurred at room temperature^36^. In other words, the repeat units of cyclic polymer and the corresponding linear counterpart should be consistent, because of the same feeding ratio of monomer to catalyst. Differently, the GPC curves (Supplementary Fig. 3) provided the *M*_n_s of 32.0 and 25.6 kg mol^−1^ for linear and cyclic copolymers (entries 1 and 2, Table 1), respectively, which was considered as a clear evidence for cyclic polymer with a longer elution time due to its smaller hydrodynamic volume compared with the linear analogue, while the precise *M*_n,NMR_ could not be calculated from the ^1^H NMR spectra by the end group analysis, because of the overlap for the signals of phenyl group on the chain end with those of aryl group.

**Table 1:** Characteristics for linear and cyclic polymers

Entry	Polymer	[BNP]:[TNP]:[BNP]:[TNP]: [BNP]:[TNP]:[BNP]:[Cat]^a^	[PBNP]:[PTNP]^b^	*M*_n_^c^ (kg mol^−1^)	PDI^c^	Yield (%)
1^d^	l-**PBNP**_40_-(b-**PTNP**_20_)_2_	20:20:0:0:0:0:0:1	40:41.3	32.0	1.35	95
2^e^	c-[**PBNP**_20_-(b-**PTNP**_20_)_2_-b-**PBNP**_20_]	10:20:10:0:0:0:0:1	40:44.8	25.6	1.31	93
3^f^	c-[**PBNP**_20_-(b-**PTNP**_20_)_2_-b-**PBNP**_20_]	10:20:10:0:0:0:0:1	40:48.9	22.9	1.36	88
4^d^	l-**PBNP**_20_-(b-**PTNP**_20_)_2_	10:20:0:0:0:0:0:1	20:39.2	22.1	1.32	93
5^f^	c-[**PBNP**_10_-(b-**PTNP**_20_)_2_-b-**PBNP**_10_]	5:20:5:0:0:0:0:1	20:37.2	15.0	1.28	91
6^d^	l-**PBNP**_20_-(b-**PTNP**_40_)_2_	10:40:0:0:0:0:0:1	–	48.6	1.25	93
7^f^	c-[**PBNP**_10_-(b-**PTNP**_40_)_2_-b-**PBNP**_10_]	5:40:5:0:0:0:0:1	20:68.2	43.7	1.31	92
8^d^	l-**PBNP**_20_-(b-**PTNP**_80_)_2_	10:80:0:0:0:0:0:1	–	81.8	1.45	92
9^f^	c-[**PBNP**_10_-(b-**PTNP**_80_)_2_-b-**PBNP**_10_]	5:80:5:0:0:0:0:1	20:148.0	67.2	1.43	95
10^d^	l-**PBNP**_20_-(b-**PTNP**_120_)_2_	10:120:0:0:0:0:0:1	–	99.2	1.39	91
11^f^	c-[**PBNP**_10_-(b-**PTNP**_120_)_2_-b-**PBNP**_10_]	5:120:5:0:0:0:0:1	20:234.4	84.5	1.41	91
12^d^	l-**PBNP**_20_-(b-**PTNP**_160_)_2_	10:160:0:0:0:0:0:1	–	115.2	1.43	95
13^f^	c-[**PBNP**_10_-(b-**PTNP**_160_)_2_-b-**PBNP**_10_]	5:160:5:0:0:0:0:1	20:328.6	96.8	1.38	94
14^d^	l-**PBNP**_20_-(b-**PTNP**_200_)_2_	10:200:0:0:0:0:0:1	–	122.3	1.45	83
15^f^	c-[**PBNP**_10_-(b-**PTNP**_200_)_2_-b-**PBNP**_10_]	5:200:5:0:0:0:0:1	20:374.7	110.5	1.36	85
16^d^	l-**PBNP**_20_-(b-**PTNP**_160_)_2_	10:160:0:0:0:0:0:1	–	110.2	1.38	91
17^f^	c-[**PBNP**_10_-(b-**PTNP**_160_)_2_-b-**PBNP**_10_]	5:160:5:0:0:0:0:1	–	93.1	1.31	89
18^d^	l-**PBNP**_30_-(b-**PTNP**_40_)_2_	15:40:0:0:0:0:0:1	30:81.7	58.9	1.39	94
19^f^	c-[**PBNP**_10_-((b-**PTNP**_20_)_2_-b-**PBNP**_10_)_2_]	5:20:5:20:5:0:0:1	30:81.1	38.2	1.35	94
20^d^	l-**PBNP**_40_-(b-**PTNP**_60_)_2_	20:60:0:0:0:0:0:1	40:89.5	87.4	1.48	83
21^f^	c-[**PBNP**_10_-((b-**PTNP**_20_)_2_-b-**PBNP**_10_)_3_]	5:20:5:20:5:20:5:1	40:86.8	44.0	1.36	85

Polymerization conditions: using **Ru-III** as catalyst, CH_2_Cl_2_ as solvent, temperature = 30 °C (or −20 °C for entries 16 and 17). Polymerization time: (30 + 30 min) for *l*-**PBNP**-(*b*-**PTNP**)_2_, (30 + 30 + 30 min) for *c*-[**PBNP**-(*b*-**PTNP**)_2_-*b*-**PBNP**], (10 + 20 + 10 + 20 + 30 min) for *c*-[**PBNP**_10_-((*b*-**PTNP**_20_)_2_-*b*-**PBNP**_10_)_2_], and (10 + 20 + 10 + 20 + 10 + 20 + 30 min) for *c*-[**PBNP**_10_-((*b*-**PTNP**_20_)_2_-*b*-**PBNP**_10_)_3_]

^a^The feeding ratios of monomers to catalyst for polymerization of di- and monofunctional monomers in sequential addition manner

^b^The block ratio of **PBNP** to **PTNP** by ^1^H NMR spectroscopy analysis

^c^Determined by GPC in THF relative to monodispersed polystyrene standards

^d^[**BNP**]_0_ = 2 × 10^−3^ mol L^−1^ refers to the initial monomer concentration of **BNP**

^e^[**BNP**]_0_ = 1 × 10^−3^ mol L^−1^ refers to the initial monomer concentration of **BNP**

^f^[**BNP**]_0_ = 5 × 10^−4^ mol L^−1^ refers to the initial monomer concentration of **BNP**

When further analyzing the ^1^H NMR spectrum of *c*-[**PBNP**_20_-(*b*-**PTNP**_20_)_2_-*b*-**PBNP**_20_] under the initial concentration (1 × 10^−3^ mol L^−1^) of difunctional **BNP** (Supplementary Fig. 2b), the weak signals of olefinic protons on the NBE ring at 6.2 ppm were still observed. By integrating the ^1^H NMR signals of olefinic protons at 6.2 ppm and those on the main chain of PNBE (H_a_) at 5.54–5.27 ppm, the molar ratio of the double bond residue on NBE to those of PNBE backbone was about 1:20, indicating that the cyclization of linear intermediate was mainly conducted and the second ladderphane **PBNP** was formed actually, even if there was a partial defective structure (Supplementary Fig. 4a). If polymerization occurred unlike this case, then polymer will have either a linear structure with a number of residual olefinic protons on the NBE ring and a larger hydrodynamic volume (Supplementary Fig. 4b) or a doubled *M*_n_ value (Supplementary Fig. 4c), which were all in contradiction with the results of ^1^H NMR and GPC analyses, meaning that the latter two cases should be of inexistence.

**Fig. 3:**
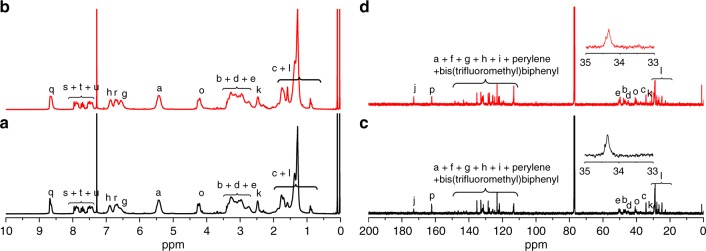
The NMR spectra of linear and monocyclic polymers. ^1^H (**a**, **b**) and ^13^C (**c**, **d**) NMR spectra of *l*-**PBNP**_20_-(*b*-**PTNP**_20_)_2_ (**a**, **c**) and *c*-[**PBNP**_10_-(*b*-**PTNP**_20_)_2_-*b*-**PBNP**_10_] (**b**, **d**) in CDCl_3_

The high reactant concentration and excessive difunctional monomer may be the key factors to cause the flawed structure of second ladderphane in polymerization process^30^. To avoid this issue, a simple way was tried by reducing the initial **BNP** concentration to 5 × 10^−4^ mol L^−1^ (entry 3, Table 1). As expected, the integral area ratio of olefinic protons from the ^1^H NMR spectrum (Supplementary Fig. 2c) decreased to 1:50, implying that the unreacted NBE group was further reduced, whereas these results were still not satisfactory as far as the perfect cyclic structure is concerned. Surprisingly, when the initial feed ratio of **BNP** to catalyst was halved to 5:1 (entry 5, Table 1), the signal of olefinic protons on the NBE ring at 6.2 ppm disappeared completely (Fig. 3b), indicating that the well-defined second ladderphane structure was indeed formed and the proposed defective cyclic structure (Supplementary Fig. 4a) was completely avoided under this optimum polymerization conditions. The calculated **PBNP**/**PTNP** ratios of 20:39.2 and 20:37.2 (entries 4 and 5, Table 1) by the ^1^H NMR analysis were compliant with the feed ratio of 20:40. Importantly, only a signal peak at 5.54–5.27 ppm in ^1^H NMR spectra (Fig. 3a, b) and a solely absorption band at 965 cm^−1^ (but no absorption appearance at 720 cm^−1^ for the *cis* double bond) in IR spectra (Supplementary Fig. 5) for the *trans* double bonds on PNBE backbone of block copolymers were observed, demonstrating that the PNBE backbone was highly *trans*-configuration. Raman spectra of polymers (Supplementary Fig. 6) showed the characteristic peak at 1606 cm^−1^ assigned to the *trans* C = C vibration (while nearly no *cis* C=C vibration appeared at about 1580 cm^−1^) in the PNBE backbone^37^, which is consistent with the results of ^1^H NMR and IR analyses. Besides, the ^13^C NMR spectra (Fig. 3c, d) of block copolymers showed that the signals of methylene carbon (C_c_) were observed as only one single peak at 34.5–34.0 ppm, suggesting that the backbone had high stereoregularity mostly because the size of *endo*-N-arylpyrrolidine NBE moiety in **BNP** and **TNP** matched well with the space requirement to control the stereoselectivity of single- and double-stranded PNBE chains during ROMP process^30–33,38,39^.

**Fig. 4:**
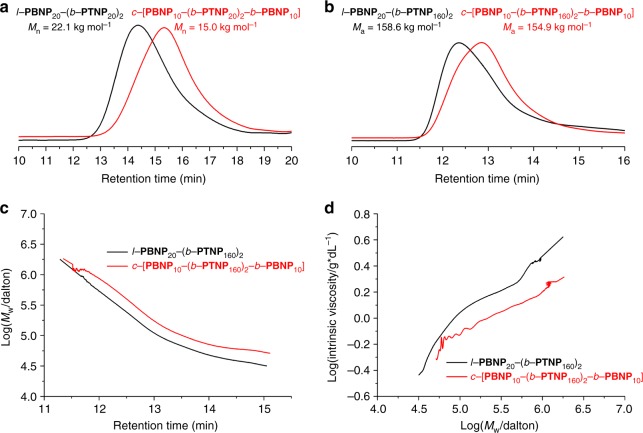
GPC traces of linear and monocyclic polymers. **a** Number-average molecular weight (*M*_n_). **b** Absolute molecular weight *(M*_a_) vs elution time (eluent = THF; the reported molecular weights were determined by light-scattering methods and are therefore considered absolute). **c** Plots of log weight-average molecular weight (*M*_w_) vs elution time. **d** Plots of log intrinsic viscosity vs log *M*_w_ (Mark–Houwink plot)

By comparing the GPC traces (Fig. 4a), the *M*_n_ of cyclic polymer was lower than that of linear analogue (15.0 vs 22.1 kg mol^−1^) due to its smaller hydrodynamic volume. The results of ^1^H NMR and GPC analyses proved that the effective cyclization was performed successfully. Inspired by the successful preparation of cyclic polymer with short repeat units, the loading of monofunctional **TNP** increased to enlarge the cyclic units and evaluate the universality of the proposed method. When the [**TNP**]/[Cat] ratios raised to 40, 80, 120, and 160 (entries 6–13, Table 1), the *M*_n_s of resulting four cyclic polymers *c*-[**PBNP**_10_-(*b*-**PTNP**_*x*_)_2_-*b*-**PBNP**_10_] (*x* = 40, 80, 120, and 160) were 43.7, 67.2, 84.5, and 96.8 kg mol^−1^ (Supplementary Fig. 7), respectively, which were lower than those (48.6, 81.8, 99.2, and 115.2 kg mol^−1^) of their linear counterparts *l*-**PBNP**_20_-(*b*-**PTNP**_*x*_)_2_. The absolute molecular weight (*M*_a_) and intrinsic viscosity of representative *l*-**PBNP**_20_-(*b*-**PTNP**_160_)_2_ and *c*-[**PBNP**_10_-(*b*-**PTNP**_160_)_2_-*b*-**PBNP**_10_] were also obtained by GPC analysis with dual angle laser light scattering and viscosity detectors (Fig. 4b). The linear and cyclic polymers had a similar *M*_a_ (158.6 vs 154.9 kg mol^−1^) because of the same feed ratio of monomer to catalyst, and the *M*_a_ values were about 1.5 times larger than those of the related *M*_n_. The more physically compacted cyclic polymer possessed smaller hydrodynamic volume and lower intrinsic viscosity than their linear analogues (Fig. 4c, d), and the viscosity discrepancy between the linear and cyclic polymers was extended as the weight-average molecular weight (*M*_w_) increase (Fig. 4d). In addition, the ^1^H NMR spectra of *c*-[**PBNP**_10_-(*b*-**PTNP**_*x*_)_2_-*b*-**PBNP**_10_] showed that the well-defined second ladderphane in all cyclic polymers was formed, because the signal of residual olefinic protons on the NBE ring was not observed (Supplementary Fig. 8). The actual **PBNP**/**PTNP** ratios of cyclic polymers evaluated by their ^1^H NMR spectra were 20:68.2, 20:148.0, 20:234.4, and 20:328.6 successively, which increased with the **TNP** loading increase and were much closer to the corresponding theoretical block ratios. Furthermore, the representative ^1^H NMR spectrum of *c*-[**PBNP**_10_-(*b*-**PTNP**_160_)_2_-*b*-**PBNP**_10_] showed no signal of olefinic protons on the NBE ring at 6.2 ppm even if the coherent accumulation number was added up to 5000 (Supplementary Fig. 9), suggesting that the second ladderphane structure was well-defined, and the proposed defective cyclic structure or impossible structures (Supplementary Fig. 4) were effectively ruled out, otherwise, the signal of unreacted olefinic protons on the NBE ring should be detected (like as those in Supplementary Figs. 2b and 2c with 5% and 2% of residual NBE groups, respectively). When the [**TNP**]/[Cat] ratio further increased to 200 (entries 14 and 15, Table 1), *c*-[**PBNP**_10_-(*b*-**PTNP**_200_)_2_-*b*-**PBNP**_10_] has an increased *M*_n_ of 110.8 kg mol^−1^, which was also lower than that (122.3 kg mol^−1^) of its linear analogous *l*-**PBNP**_20_-(*b*-**PTNP**_200_)_2_ with the yield of about 85%, and the resulting **PBNP**/**PTNP** ratio of 20:374.7 was lower than the theoretical value of 20:400. The PDIs of all polymers were narrow between 1.25 and 1.45, and the *trans*-configuration did not change in the successive ROMP process for the preparation of linear and cyclic polymers.

These results strongly proved that the cyclic polymers with small to large ring size and perfect ring structure could be achieved successfully by the blocking-cyclization technique.

In general, the chain-transfer rate was much slower than the chain-propagation rate in ROMP of NBE derivatives initiated by **Ru-III** at room temperature^36,40^, namely, it was rare to encounter the problem that the active species at the end of polymer chains were transferred or the chain termination reactions happened before cyclizing the linear intermediate. To preliminarily examine the influence of temperature on chain-transfer reaction, ROMP of **BNP** and **TNP** with the feed ratio of 10:160 at −20 °C (entries 16 and 17, Table 1) was selected as a representative case, generating linear *l*-**PBNP**_20_-(*b*-**PTNP**_160_)_2_ and its cyclic counterpart *c*-[**PBNP**_10_-(*b*-**PTNP**_160_)_2_-*b*-**PBNP**_10_] with the *M*_n_s and PDIs of 110.2 kg mol^−1^ and 1.38 as well as 83.1 kg mol^−1^ and 1.31 (Supplementary Fig. 10), respectively, which were slightly lower and narrower than those of the corresponding polymers obtained with the same feed ratio at 30 °C (entries 12 and 13, Table 1), predicating that the chain transfer reaction was unconspicuous at room temperature, and the negative effect of temperature on the formation of cyclic polymers could be ignored. In a word, the synthesis of monocyclic polymers with short to long repeat units was realized by ROMP under the conventional reaction conditions.

### Synthesis and characterization of bis- and tricyclic polymers

Compared to the traditional techniques for synthesizing cyclic polymers, the predominant advantage of the blocking-cyclization technique is the easy synthesis of multicyclic polymers with controlled *M*_n_ by successive ROMP of di- and monofunctional monomers in one-pot procedure. On the basis of the optimized conditions for monocyclic polymers under low monomer loading, the blocking-cyclization technique was tried to prepare bis- and tricyclic polymers, *c*-[**PBNP**_10_-((*b*-**PTNP**_20_)_2_-*b*-**PBNP**_10_)_2_] and *c*-[**PBNP**_10_-((*b*-**PTNP**_20_)_2_-*b*-**PBNP**_10_)_3_], by successive ROMP via alternating monomer addition in five and seven times, respectively. Similarly, comparing the bis- and tricyclic polymers with their linear counterparts *l*-**PBNP**_30_-(*b*-**PTNP**_40_)_2_ and *l*-**PBNP**_40_-(*b*-**PTNP**_60_)_2_, the approximated **PBNP**/**PTNP** ratios for the above two pairs of polymers were calculated to be 30:81.1 vs 30:81.7 and 40:86.8 vs 40:89.5 (entries 18–21, Table 1) by the ^1^H NMR analysis (Supplementary Figs. 26 and 27), and the *M*_n_s (Supplementary Figs. 28 and 29) of cyclic polymers were less than those of linear analogues (38.2 vs 58.9 kg mol^−1^ and 44.0 vs 87.4 kg mol^−1^, respectively), indicating that the successive cyclization was conducted effectively. What is more important, for the bis- and tricyclic polymers, the well-defined ladderphane segments were generated and thus the perfect macrocyclic structures were obtained, because the signal of residual olefinic protons on the NBE ring was never observed in ^1^H NMR spectra (Supplementary Figs. 26 and 27). In addition, the ^1^H NMR spectra of all polymers had only a signal peak at 5.54–5.27 ppm, and the IR spectra (Supplementary Figs. 30 and 31) showed the solely absorption band at 965 cm^−1^ for the *trans* double bond without the absorption appearance at 720 cm^−1^ for the *cis* double bond, demonstrating that the PNBE backbones were highly *trans*-configuration, which were similar with those of monocyclic polymers. Therefore, it can be concluded from the above investigations that the developed blocking-cyclization technique is an efficient strategy for synthesizing mono- and even multicyclic polymers.

### Post-polymerization of monocyclic polymers

Post-polymerization is an effective way to modify polymers with varied structures and improved properties. As for *c*-[**PBNP**_10_-(*b*-**PTNP**_x_)_2_-*b*-**PBNP**_10_], it has the unsaturated PNBE backbone and could be easily modified by triazolinedione (TAD)-based Alder-ene chemistry^41^. Taking *c*-[**PBNP**_10_-((*b*-**PTNP**_160_)_2_-*b*-**PBNP**)_10_] as an example, its post-polymerization modification was conducted to make it be transformed into the bulky POSS moiety-contained *c*-[**PBNP**_10_-((*b*-**PTNP**_160_)_2_-*b*-**PBNP**)_10_]-**POSS** (Supplementary Fig. 37) by using the molar ratio of polyhedral oligomeric silsesquioxane propylisobutyl-1,2,4-triazoline-3,5-dione (**POSS-TAD**) (Supplementary Fig. 39) to the double bonds on PNBE backbone (1:1). After connecting POSS moieties, the position of double bonds on the PNBE backbone of *c*-[**PBNP**_10_-((*b*-**PTNP**_160_)_2_-*b*-**PBNP**)_10_]-**POSS** was isomerized, and a new signal peak at 3.62–3.47 ppm ascribed to the introduced methylene group (H_w_) of POSS moiety was observed in its ^1^H NMR spectrum (Supplementary Fig. 40a) by comparison to the signals of *c*-[**PBNP**_10_-((*b*-**PTNP**_160_)_2_-*b*-**PBNP**)_10_] (Fig. 3b), clearly indicating the successful modification. In addition, by virtue of the peak area ratio of H_w_ to that of double-bond protons (H_a_) on the PNBE backbone at 5.59–5.24 ppm, the quantitative incorporated **POSS-TAD** was calculated as around 25% in this case, which was lower than the feed ratio, because the POSS moieties attached to the PNBE backbone prevented the adjacent double bond from reacting with incoming **POSS-TAD**, due to the steric hindrance of bulky POSS moiety. The signals of methylene carbon (C_c_) in the ^13^C NMR spectrum (Supplementary Fig. 40b) of *c*-[**PBNP**_10_-(*b*-**PTNP**_20_)_2_-*b*-**PBNP**_10_]-**POSS** were observed as three asymmetrical single peaks at 34.5–34.0 ppm, indicating the disruption of stereoregularity microstructure feature of PNBE backbone. The GPC curve showed a monomodal peak with the *M*_n_ and PDI of 206.4 kg mol^−1^ and 1.45 (Supplementary Fig. 41), respectively, and the peak position shifted to a high molecular weight region comparing to that of *c*-[**PBNP**_10_-((*b*-**PTNP**_160_)_2_-*b*-**PBNP**)_10_].

### Topology of cyclic polymers

When the single block copolymer chains were dispersed uniformly in the highly diluted good solvent, the inter-chain interactions could be ignored. In dynamic light scattering (DLS) testing, the linear *l*-**PBNP**_20_-(*b*-**PTNP**_20_)_2_ has one distinct peak with a dominating volume-average hydrodynamic diameter (*D*_h_) of 4–5 nm corresponding to a single macromolecule at a low concentration of 0.1 mg mL^−1^ in CHCl_3_, while the *D*_h_ of cyclic *c*-[**PBNP**_10_-((*b*-**PTNP**_20_)_2_-*b*-**PBNP**_10_)] with the same repeat units reduced to 3–4 nm (Supplementary Fig. 47). This was because that the confined **PTNP** segments in a single cyclic polymer molecule was easy to entangle itself by the π–π stacking effect of pendants due to the closer molecular distance in comparison to the corresponding linear polymer molecule, resulting in a smaller hydrodynamic size. As the repeat units increase, the *D*_h_s of all copolymers had a slight increase and the size of cyclic polymers was still smaller than the corresponding linear polymers. When the copolymers incorporated the POSS moiety, the sizes of all copolymers increased, and DLS diagrams of copolymers displayed two peaks with a dominating *D*_h_ of about 7 nm and a little small *D*_h_ of about 20 nm in CHCl_3_ (Supplementary Fig. 48). Noteworthy, the *D*_h_s of POSS-modified cyclic polymers had no obvious difference with those of linear counterparts (Supplementary Table 2), because the intra-chain entanglement junctions disappeared and the POSS-modified cyclic macromolecule was free from constriction.

**Fig. 5:**
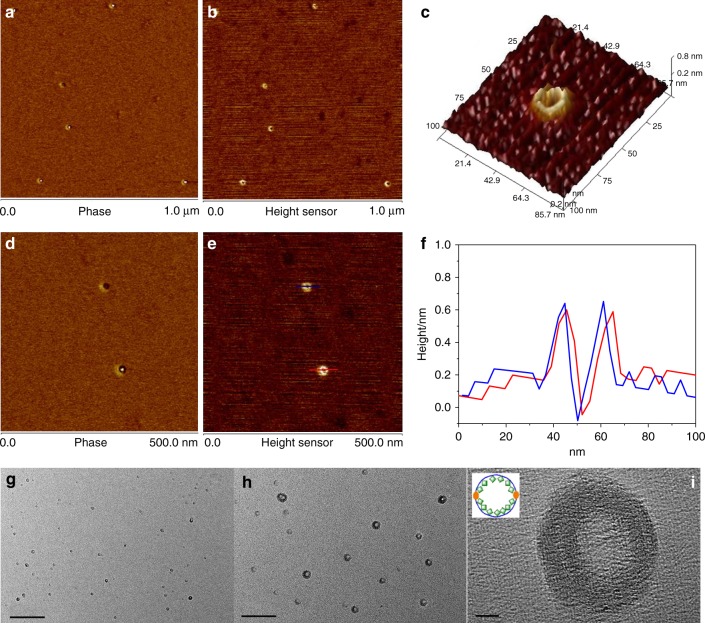
Topology of monocyclic polymer. **a**–**e** AFM phase (**a**, **d**) and height (**b**, **e**) images of *c*-[**PBNP**_10_-(*b*-**PTNP**_160_)_2_-*b*-**PBNP**_10_]-**POSS**. **c** 3-D plot of toroidal feature. **f** Profile analysis of cyclic polymer marked by the red and blue lines in (**e**). **g**–**i** TEM images of *c*-[**PBNP**_10_-(*b*-**PTNP**_160_)_2_-*b*-**PBNP**_10_]-**POSS** in CHCl_3_ at 0.005 mg mL^−1^ (scale bar: 500, 100, and 5 nm for **g**, **h**, and **i**, respectively) and the inset in (**i**) is the diagrammatic sketch of cyclic topology

Further insight into the visualization of the single cyclic polymer molecule was carried out by atomic force microscopy (AFM). AFM phase and height images were a strong supporting evidence for the cyclic polymer topology^42–44^. Samples of *c*-[**PBNP**_10_-(*b*-**PTNP**_160_)_2_-*b*-**PBNP**_10_], *c*-[**PBNP**_10_-(*b*-**PTNP**_160_)_2_-*b*-**PBNP**_10_]-**POSS**, and *l*-**PBNP**_20_-(*b*-**PTNP**_160_)_2_-**POSS** were prepared by spin-coating a polymer solution at 50 ng mL^−1^ in CHCl_3_ onto the freshly cleaved mica. According to the feed ratio ([**TNP**]/[Cat]) of 160:1, the repeat units were about 320 because the single cyclic polymer molecule had two **PTNP** segments. Based on the length of each repeat unit of 0.65 nm ^27^, the circumference and diameter of macrorings were about 208 and 66 nm, respectively. The AFM height image of unmodified *c*-[**PBNP**_10_-(*b*-**PTNP**_160_)_2_-*b*-**PBNP**_10_] was shown in Supplementary Fig. 49. Typically, the irregular nanoparticles with the average diameters and heights of 15–25 nm and 0.6–0.8 nm were observed, respectively, and there were no any cyclic features due to the intra-chain entanglement. When the cyclic polymers incorporated the POSS moieties on the PNBE backbone, the intra-chain entanglement of **PTNP** segments in *c*-[**PBNP**_10_-(*b*-**PTNP**_160_)_2_-*b*-**PBNP**_10_]-**POSS** was restrained, and the outer and inner diameters of macrorings could be observed as 25–35 and 8–12 nm (Fig. 5a–e), respectively, which was less than the theoretical values, indicating that the cyclic polymer backbone was not fully extended. The heights of macrorings ranged between 0.6 and 0.8 nm, and the corresponding analysis of the line scan revealed highly uniform profile features (Fig. 5f). Importantly, no any linear topology was found in Fig. 5a–e and Supplementary Fig. 50, suggesting that the adopted blocking-cyclization technique was really an effective strategy for preparing high-molecular-weight cyclic polymers.

For *l*-**PBNP**_20_-(*b*-**PTNP**_160_)_2_-**POSS**, however, only solid nanospheres with the average diameter of 30–35 nm and height of 1 nm were observed in the AFM images (Supplementary Fig. 51), while there were no any macrorings, indicating that the POSS moiety itself did not bring the linear polymer to form the cyclic topology, just to restrain the intra-chain entanglement of cyclic polymer and expand the single-stranded chains away from the close proximity to each other.

The single cyclic macromolecular topology other than the self-assembly or aggregated morphology was hard to be observed by the transmission electron microscopy (TEM) analysis, and therefore there had been seldom reported to date^44^. As stated, *c*-[**PBNP**_10_-(*b*-**PTNP**_160_)_2_-*b*-**PBNP**_10_] and *c*-[**PBNP**_10_-(*b*-**PNP**_80_)_2_-*b*-**PBNP**_10_] did not exhibit the macrorings but showed the solid nanosphere morphology in their TEM images with the average diameters of about 25 and 20 nm (Supplementary Figs. 52 and 53), respectively, because the strong π–π stacking effect of large aromatic pendants in the two cyclic polymers caused the intra-chain entanglement. Fortunately, the bulky POSS-modified cyclic molecular topology of *c*-[**PBNP**_10_-(*b*-**PTNP**_160_)_2_-*b*-**PBNP**_10_]-**POSS** was clearly seen from the TEM images with the average outer and inner diameters of 25–35 and 10–14 nm at different sample concentrations (Fig. 5g–i and Supplementary Fig. 54), while the TEM images of linear *l*-**PBNP**_20_-(*b*-**PTNP**_160_)_2_ and *l*-**PBNP**_20_-(*b*-**PTNP**_160_)_2_-**POSS** showed only solid nanospheres with the average diameters of about 30 and 35 nm (Supplementary Figs. 55 and 56), respectively, which were almost consistent with those of AFM analysis. In particular, the POSS moieties connected to the PNBE backbone enabled the polymer chain to expand, and thus the POSS-modified cyclic polymer exhibited a unique concentric macroring molecular topology due to the difference in electron cloud density (Fig. 5i), in which the dark domain represented the POSS moieties with higher electron density and the bright domain was ascribed to the cyclic chains with lower electron density.

## Discussion

We have developed the blocking-cyclization technique, which could be considered as the third strategy and a versatile platform for precisely synthesizing the mono- and multicyclic polymers by successive ROMP of mono- and di-functional NBE derivatives. The cyclic repeat units and ring number could be controlled by simply tuning the loading ratios and adding times of monomers. In cyclic polymers, the π–π stacking effect of the single-stranded segments generates the intra-chain entanglement junctions, resulting in the *T*_g_, photophysical feature, hydrodynamic size, and dielectric loss of each cyclic polymer are apparently different from those of linear counterpart. By virtue of Alder-ene chemistry, the cyclic polymer with unsaturated PNBE backbone was modified by POSS moiety-contained TAD so that the intra-chain entanglement junctions depressed. The cyclic features were fully demonstrated through hydrodynamic volume, *T*_g_, UV–vis absorption, fluorescence emission, and dielectric loss; meanwhile, the unique cyclic molecular topology was revealed by AFM and TEM images. Thanks to the well-defined ladderphane structure, which will enable this blocking-cyclization strategy to open a new way and have a potential to cyclic polymers with various backbone and more ring number via commercial Ru-based catalyst and conventional ROMP.

## Methods

### Materials

The materials and the synthesis of chemicals are described in Supplementary Methods.

### Instrumentation

^1^H (500 MHz) and ^13^C (125 MHz) NMR spectra were recorded using tetramethylsilane as an internal standard on a Bruker DPX spectrometer. UV–vis absorption spectra were measured on a UV-1800 spectrometer with the concentration of 0.01 mg mL^−1^. Fluorescence measurements were performed on an F-7000 spectrofluorometer equipped with a xenon light source (UXL-150S, Ushio). The emission and excitation slit widths were 5 nm and 5 nm, respectively. The samples were excited at 360 nm and emission spectra were recorded from 360 to 600 nm. IR spectra were recorded on a Perkin Elmer Spectrum using KBr pellets. Gel permeation chromatography (GPC) was used to calculate relative molecular weight and molecular weight distribution equipped with a Waters 1515 Isocratic HPLC pump, a Waters 2414 refractive index detector, and a set of Waters Styragel columns (7.8 × 300 mm, 5 mm bead size; 10^3^, 10^4^, and 10^5^ Å pore size). GPC measurements were carried out at 40 °C using THF as the eluent with a flow rate of 1.0 mL min^−1^, and the system was calibrated with polystyrene standard. The intrinsic viscosity and absolute molecular weight of polymers were determined using high-performance size-exclusion chromatography (HPSEC), Viscotek (Viscotek TDAmax) with a differential viscometer (DV), right angle laser-light scattering (RALLS, Viscotek), low-angle laser-light scattering (LALLS, Viscotek), and refractive index (RI) detectors. The column set consisted of a PL 10 mm guard column (50 × 7.5 mm^2^) followed by one Viscotek T6000 column (8.0 × 300 mm, 10 mm bead size; 10^4^ Å pore size) and one Viscotek T4000 column (8.0 × 300 mm, 6 mm bead size; 1.5 × 10^3^ Å pore size). Matrix-assisted laser desorption ionization time-of-flight mass spectroscopy (MALDI-TOF MS) was performed on a flight mass spectrometer (5800). Mass spectrum was recorded in linear mid-mass positive mode. The matrix, α-cyano-4-hydroxycinnamic acid, was dissolved in THF (10 mg mL^−1^), and the solution was mixed with the polymer solution (50 mg mL^−1^ in CHCl_3_). The 1.0 µL analyte solution was spotted directly onto the thin-layer formed by depositing 1.0 µL saturated NaI cationizing agent in methanol. Thermal gravimetric analysis (TGA) was performed using an SDTA851e/SF/1100 TGA instrument under nitrogen flow at a heating rate of 10 °C min^−1^ from 40 to 600 °C. Differential scanning calorimeter (DSC) was performed on a Q2000 DSC system in nitrogen atmosphere. An indium standard was used for temperature and enthalpy calibrations. All the samples were first heated from 40 to 250 °C and held at this temperature for 3 min to eliminate the thermal history, and then, they were cooled to room temperature and heat again from 40 to 250 °C at a heating or cooling rate of 10 °C min^−1^. AFM images were taken using a Nanoscope IV Scanning Probe Microscope Controller (Digital Instruments, Veeco Metrology Group) in tapping mode in air at room temperature using silicon tips (spring constant = 40–50 N/m, resonance frequency = 170–190 kHz, and tip radius of curvature <10 nm). The samples were prepared by spin casting dilute solutions (50 ng mL^−1^) in chloroform onto freshly cleaved mica for the cyclic copolymers. TEM images were recorded on the JEOL2100F microscopes operating at 120 kV. Samples for TEM measurement were prepared by depositing a drop of THF solution with a different concentration on the copper grids coated with carbon, followed by air-drying. Additionally, the samples were not stained before measurement because the electron density difference between the two blocks provided sufficient contrast for TEM imaging. The hydrodynamic diameter was determined by means of DLS analysis using a Malvern Zetasizer Nano-ZS light scattering apparatus (Malvern Instruments, UK) with a He–Ne laser (633 nm, 4 mW). The Nano ZS instrument incorporates noninvasive backscattering (NIBS) optics with a detection angle of 173°. The *z*-average diameter of the sample was automatically provided by the instrument using cumulate analysis. Dielectric measurements were carried out by a Novocontrol BDS40 dielectric spectrum analyzer over the frequency range of 100 Hz to 1 MHz at room temperature, and a contacting electrode method was used. The edge side of the guarded electrode is 0.3 mm. The capacitance and the dissipation factor (dielectric loss, or tan*δ*) of the tested films were recorded.

## Electronic supplementary material

Supplementary Information

## Data Availability

The authors declare that the data supporting this study are available within the paper and its Supplementary Information File. All other data is available from the authors upon reasonable request.
