# Supplementary material for: Blocking-cyclization technique for precise synthesis of cyclic polymers with regulated topology
Source: Nat Commun. 2018 Dec 14;9:5310. doi: 10.1038/s41467-018-07754-1 (PMC6294010; doi:10.1038/s41467-018-07754-1)
Supplement: Supplementary file 1 — Supplementary Information [file 41467_2018_7754_MOESM1_ESM.pdf]

Supplementary Information File

Blocking-cyclization technique for precise synthesis of cyclic polymers with regulated topology

Xie et al.

Supplementary Methods

Supplementary Discussion

Supplementary Note 1. Characteristics of linear and monocyclic polymers

Supplementary Note 2. More insight into the ladderphane and the rigid single-stranded polymer structure

Supplementary Note 3. Verification of cyclic topology by hydrolysis of cyclic polymer

Supplementary Note 4. Characteristics of bis- and tricyclic polymers

Supplementary Note 5. Thermal properties of linear and cyclic polymers

Supplementary Note 6. Post-polymerization of monocyclic polymers

Supplementary Note 7. Photophysical properties of linear and cyclic polymers

Supplementary Note 8. Hydrodynamic diameter and topology of linear and cyclic polymers

Supplementary Note 9. Dielectric feature of linear and cyclic polymers

Supplementary References

Supplementary Methods

Materials

Bis(norbornene pyrrolidine phenyl perylene bisimide) (**BNP**),[1] *endo*-N-3,5-bis(trifluoromethyl)biphenyl-norbornene pyrrolidine (**TNP**),[2] and [1,3-bis(2,4,6-trimethylphenyl)-4,5-dihydroimidazol-2-ylidene][bis(3-bromopyridine)]benzylidene ruthenium dichloride (**Ru-III**)[3] were prepared according to the previous procedures. Aminopropylisobutyl polyhedral oligomeric silsesquioxane (NH_2-POSS) was obtained from Energy Chemical Company. 2-Ethylhexyl acrylate, norbornene-5,6-*endo*-dicarboxylic anhydride (NDA), trichloroisocyanuric aid, 4-nitrophenyl chloroformate, triethylamine, and K_2CO_3 (99%) were purchased from Shanghai Chemical Reagents Company. Solvents were distilled over drying agents under nitrogen prior to use: dichloromethane (CH_2Cl_2), trichloromethane ($CHCl_3$) from calcium hydride. All reactions were performed under dry nitrogen atmosphere using standard Schlenk-line technique.

Synthesis of bis(norbornene imide) diethylene glycol di(dodecanoic acid ester) (BNI)

A solution of 12-amino dodecanoic acid (2.15 g, 10 mmol) in 10 mL of HAc was added dropwise to the stirred solution of norbornene-5,6-*endo*-dicarboxylic anhydride (1.64 g, 10 mmol) in 50 mL of HAc, DMAP (0.244 g, 2 mmol) was then added at room temperature, and the mixture was stirred at 80 °C for 5 hour. Upon cooling, the reaction mixture was dropped into an excess of water, and filtered, and a yellow oily liquid was obtained (3.6 g, 95%).

To a solution of yellow oily liquid (3.6 g, 9.5 mmol) in 50 mL of anhydrous CH_2Cl_2, diethylene glycol (0.41 g, 4 mmol), DMAP (0.34 g, 2.8 mmol), and EDCI•HCl (3.63 g, 19 mmol) were added under a nitrogen atmosphere in an ice bath. The reaction mixture was stirred for 3 d at room temperature. It washed with distilled water (3×100 mL) and saturated NaCl aqueous solution (2×50 mL). The organic layer was dried with Na_2SO_4, filtered and concentrated under reduced pressure. The solid was purified by column chromatography on silica gel, eluting with a CH_2Cl_2 to afford the primrose yellow oily liquid **BNI** (3.2 g, 94%). ^1H NMR (500 MHz, $CDCl_3$, ppm): δ 6.10 (s, 4H, CH=CH), 4.23 (s, 4H, CH_2NCO), 3.71 (s, 4H, $OCOCH_2$), 3.39 (s, 4H, CH_2N), 3.32 (s, 4H, OCCH), 3.24 (s, 4H, OCH_2C*H*O), 2.34 (t, 4H, J = 9.02 Hz, $OCOCH_2$), 1.78-1.13 (m, 40H, =CHCHCH_2 + $OCOCH_2(CH_2)_9CH_2N$). ^{13}C NMR (125 MHz, $CDCl_3$, ppm): δ 177.64,173.54, 134.29, 68.98, 63.29, 52.13, 45.72, 44.78, 38.09, 33.91, 29.16, 27.57, 26.64, 24.69, 20.88.

Synthesis of (norbornene pyrrolidine) phenyl perylene bisimide (NP)

To a suspension of 1,6,7,12-tetrachloroperylene-3,4:9,10-tetracarboxylic dianhydride (5.3 g,

10 mmol) in 50 mL of toluene, 2-ethylhexylamine (1.42 g, 11 mmol) and 12-amino dodecanoic acid (2.37 g, 11 mmol) were added and stirred under a N_2 atmosphere at reflux for 24 h. After being cooled to room temperature, 100 mL of CH_2Cl_2 was added, and a red precipitate was formed. The CH_2Cl_2 phase was evaporated to remove solvent, and purified by column chromatography on silica gel using CH_2Cl_2/EA (10:1) as eluent. The product **PBI-COOH** was obtained as a dark red powder (2 g, 40%).

To a solution of **PBI-COOH** (1 g, 1.2 mmol) in 50 mL of anhydrous CH_2Cl_2, *endo*-**HNP** (0.37 g, 1.43 mmol) and DMAP (0.04 g, 0.36 mmol) were added under stirring at ice-water bath for 5 min, then EDCI•HCl (0.34 g, 1.8 mmol) was added to the mixture, stirred at room temperature for 3 days. The solvent was removed under reduced pressure. The solid was purified by column chromatography on silica gel using CH_2Cl_2/CH_3OH (200:1) as eluent. The red powder **NP** (1.04 g, 80%) was obtained. ^{1}H NMR (500 MHz, $CDCl_3$, ppm): δ 8.68 (s, 4H, pery), 7.09-7.02 (d, 2H, J = 6.82 Hz, NCCH), 6.41-6.38 (d, 2H, J = 6.82 Hz, CHCOCO), 6.14 (s, 2H, CH=CH), 4.27-4.08 (br, 4H, CH_2NCO), 3.27-3.05 (dd, 4H, CH_2NAr), 2.95 (s, 4H, =CHCHC*H*), 2.91-2.84 (d, J = 9.50 Hz, 4H, =CHC*H*CH), 2.50-2.47 (t, 4H, J = 9.02 Hz, $OCOCH_2$), 1.77-1.17 (m, 40H, =CHCHCH_2 + $OCOCH_2(CH_2)_9CH_2N$). ^{13}C NMR (125 MHz, $CDCl_3$, ppm): δ 173.40, 162.36, 145.81, 140.52, 135.95, 135.36, 132.94, 131.44, 128.59, 127.27, 121.79, 52.21, 50.85, 46.52, 45.63, 40.98, 34.31, 29.49, 29.46, 29.41, 29.31, 29.23, 29.13, 28.10, 27.06, 25.07,13.08, 9.24.

General procedure for metathesis polymerization

Typically, the **Ru-III**-initiated successive ring-opening metathesis polymerization (ROMP) of bi- and monofunctional norbornene derivatives were carried out in a Schlenk tube under dry nitrogen atmosphere at 30 °C in CH_2Cl_2 for a preset time. After confirmed the monomer conversion by TLC, ethyl vinyl ether (0.2 mL) was added to the reaction mixture and stirred for further 1 h, and the mixture was concentrated and poured into an excess of acetone. The dark red polymer was washed with acetone, and dried in a vacuum oven at 40 °C to a constant weight.

Successive ROMP of BNP and TNP for synthesis of linear copolymers

A 100 mL of Schlenk tube was charged with bifunctional monomer **BNP** (54 mg, 0.04 mmol) dissolved in 20 mL of CH_2Cl_2. **Ru-III** (3.6 mg, 4 μmol) and monofunctional monomer **TNP** (34 mg, 0.08 mmol) were dissolved in 1 mL of CH_2Cl_2 in two 10 mL of Schlenk tubes, respectively. After degassed with three freeze-vacuum-thaw cycles, **Ru-III** solution was injected into the **BNP** solution via a syringe under vigorous stirring at 30 °C for 0.5 h, and

then **TNP** solution was injected into the reaction mixture and stirred for further 0.5 h, affording the double-stranded linear triblock copolymer poly[bis(norbornene pyrrolidine phenyl perylene bisimide)]-{*block*-poly[N-3,5-bis(trifluoromethyl)biphenyl-norbornene pyrrolidine]}$_2$, *l*-**PBNP**$_{20}$-(*b*-**PTNP**$_{20}$)$_2$ (82.1 mg, 93%). ^{1}H NMR (500 MHz, $CDCl_3$): δ 8.73-8.54 (br, pery), 8.01-7.30 (br, CCCH + F_3CCCH + F_3CCCHCCF_3), 7.01-6.38 (br, NCC*H*CH), 5.59-5.26 (br, *trans*-CH on PNBE backbone), 4.34-4.06 (br, CHCH_2O), 3.43-2.24 (br, CH_2NAr + =CHC*H*CH + =CHCHC*H* + NCH_3 + OCOCH_2), 2.02-0.77 (br, =CHCHCH_2 + OCOCH_2(CH_2)$_9$CH_2N). ^{13}C NMR (125 MHz, $CDCl_3$): δ 172.59, 161.85, 148.18, 146.22, 143.62, 141.01, 135.29, 132.70, 131.38, 129.64, 128.48, 127.34, 126.03, 124.40, 123.11, 122.76, 122.06, 113.33, 49.67, 46.59, 45.29, 40.07, 34.56, 30.96, 29.20, 28.12, 27.04, 25.27, 22.71. IR (KBr): 2925 (CH_2), 2857 (C=C), 1750 (C=O), 1699, 1664, 1609, 1470, 1380, 1330, 1290, 1178, 1135, 1062, 970, 923, 912, 840, 820, 705, 683, 557 cm^{-1}. GPC: M_n = 23.2 kg mol^{-1}, PDI = 1.38.

Successive ROMP of BNP, TNP, and BNP for synthesis of monocyclic polymers

Two copies of bifunctional monomers **BNP** (27 mg, 0.02 mmol) were dissolved in 40 mL and 10 mL of CH_2Cl_2 in 250 mL and 50 mL of Schlenk tubes separately, as well as **Ru-III** (3.6 mg, 4 μmol) and monofunctional monomer **TNP** (34 mg, 0.08 mmol) were dissolved in 1 mL of CH_2Cl_2 in two 10 mL of Schlenk tubes, respectively. After degassed with three freeze-vacuum-thaw cycles, **Ru-III** solution was injected into the first **BNP** solution in a 250 mL of Schlenk tube via a syringe under vigorous stirring at 30 °C for 0.5 h, and then **TNP** was injected into the reaction solution and stirred for further 0.5 h. At last, the second 40 mL of **BNP** (27 mg, 0.02 mmol) solution was injected into the reaction mixture and stirred for another 0.5 h, affording the monocyclic block copolymer poly[bis(norbornene pyrrolidine phenyl perylene bisimide)]-[*block*-poly(N-3,5-bis(trifluoromethyl)biphenyl-norbornene pyrrolidine)]$_2$-*block*- poly[bis(norbornene pyrrolidine phenyl perylene bisimide)], *c*-[**PBNP**$_{10}$-(*b*-**PTNP**$_{20}$)$_2$-*b*-**PBNP**$_{10}$] (81.5 mg, 91%). ^{1}H NMR (500 MHz, $CDCl_3$): δ 8.74-8.59 (br, pery), 8.03-7.33 (br, CCCH + F_3CCCH + F_3CCCHCCF_3), 7.02-6.35 (br, NCC*H*CH + OCCH_2), 5.59-5.26 (br, *trans*-CH on PNBE backbone), 4.35-4.04 (br, CHCH_2O), 3.42-2.22 (br, CH_2NAr + =CHC*H*CH + =CHCHC*H* + NCH_3 + OCOCH_2), 2.06-0.79 (br, =CHCHCH_2 + OCOCH_2(CH_2)$_9$CH_2N). ^{13}C NMR (125 MHz, $CDCl_3$): δ 171.99, 161.72, 148.19, 146.23, 143.51, 141.21, 135.23, 132.75, 131.39, 129.62, 128.59, 127.35, 126.02, 124.38, 123.24, 122.78, 122.08, 113.34, 49.69, 46.60, 45.39, 40.18, 34.57, 30.98, 29.25, 28.15, 27.07, 25.37, 22.74. IR (KBr): 2925 (CH_2), 2860 (C=C), 1753 (C=O), 1700, 1664, 1609, 1470, 1380, 1330,

1292, 1178, 1132, 1064, 965, 925, 915, 830, 823, 709, 684, 558 cm^{-1}. GPC: M_n = 16.2 kg mol^{-1}, PDI = 1.32.

Successive ROMP of BNP and NP for synthesis of linear copolymers

A 100 mL of Schlenk tube was charged with bifunctional monomer **BNP** (54 mg, 0.04 mmol) dissolved in 20 mL of CH_2Cl_2. **Ru-III** (3.6 mg, 4 μmol) and monofunctional monomer **NP** (137.3 mg, 0.32 mmol) were dissolved in 2 mL of CH_2Cl_2 in two 10 mL of Schlenk tubes, respectively. After degassed with three freeze-vacuum-thaw cycles, **Ru-III** solution was injected into the **BNP** solution via a syringe under vigorous stirring at 30 °C for 0.5 h, and then **NP** solution was injected into the reaction mixture and stirred for further 0.5 h, affording the double-stranded linear triblock copolymer poly[bis(norbornene pyrrolidine phenyl perylene bisimide)]-[*block*-poly(norbornene pyrrolidine phenyl perylene bisimide)]$_2$, *l*-$\mathbf{PBNP}_{20}$-(*b*-$\mathbf{PNP}_{80}$)$_2$ (180 mg, 94%). GPC: M_n = 106.1 kg mol^{-1}, PDI = 1.33.

Successive ROMP of BNP, NP, and BNP for synthesis of monocyclic polymers

Two copies of bifunctional monomers **BNP** (27 mg, 0.02 mmol) were dissolved in 40 mL and 10 mL of CH_2Cl_2 in 250 mL and 50 mL of Schlenk tubes separately, as well as **Ru-III** (3.6 mg, 4 μmol) and monofunctional monomer **NP** (137.3 mg, 0.32 mmol) were dissolved in 2 mL of CH_2Cl_2 in two 10 mL of Schlenk tubes, respectively. After degassed with three freeze-vacuum-thaw cycles, **Ru-III** solution was injected into the first **BNP** solution in a 250 mL of Schlenk tube via a syringe under vigorous stirring at 30 °C for 0.5 h, and then **NP** was injected into the reaction solution and stirred for further 0.5 h. At last, the second 40 mL of **BNP** (27 mg, 0.02 mmol) solution was injected into the reaction mixture and stirred for another 0.5 h, affording the monocyclic block copolymer poly[bis(norbornene pyrrolidine phenyl perylene bisimide)]-[*block*-poly(norbornene pyrrolidine phenyl perylene bisimide)]$_2$-*block*-poly[bis(norbornene pyrrolidine phenyl perylene bisimide)], *c*-[$\mathbf{PBNP}_{10}$-(*b*-$\mathbf{PNP}_{80}$)$_2$-*b*-$\mathbf{PBNP}_{10}$] (175 mg, 92%). ^{1}H NMR (500 MHz, $CDCl_3$): δ 8.76-8.56 (br, pery), 6.98-6.75 (br, OCC*H*), 6.68-6.44 (br, NCC*H*CH) 5.63-5.25 (br, CH on PNBE backbone), 4.30-4.02 (br, CHCH_2O), 3.36-2.24 (br, CH_2NAr + =CHC*H*CH + =CHCHC*H* + NCH_3 + OCOCH_2), 1.99-0.79 (br, =CHCHCH_2 + OCOCH_2(CH_2)$_9$CH_2N). GPC: M_n = 89.2 kg mol^{-1}, PDI = 1.29.

ROMP of BNP

A 250 mL of Schlenk tube was charged with monomer **BNP** (54 mg, 0.04 mmol) dissolved in $CHCl_3$ (40 mL). In another 25 mL of Schlenk tube, **Ru-III** (3.6 mg, 4 μmol) was dissolved in

$CHCl_3$ (1 mL). After degassed in three freeze-vacuum-thaw cycles, the catalyst solution of **Ru-III** was then injected into the monomer solution via a syringe under vigorous stirring at 30 ^{o}C for 0.5 h, generating the corresponding poly[bis(norbornene pyrrolidine phenyl perylene bisimide)] **PBNP**$_{20}$ (53 mg, 99%). ^{1}H NMR (500 MHz, $CDCl_3$, ppm): δ 8.72-8.55 (br, pery), 7.02-6.75 and 6.68-6.41 (br, CH=CH on phenyl), 5.75-5.28 (br, *trans*-CH on backbone), 4.37-4.03 (br, CH_2NCO), 3.33-3.00 (br, =CHCHC*H*), 2.98-2.71 (br, =CHC*H*CH), 2.68-2.17 (br, OCOCH_2), 1.97-1.20 (br, =CHCHCH_2 + CH_2OCO(CH_2)$_9$$CH_2$N). GPC: M_n = 17.1 kg mol^{-1}, PDI = 1.42.

ROMP of TNP

A 250 mL of Schlenk tube was charged with monomer **TNP** (275 mg, 0.64 mmol, 320 equiv) dissolved in $CHCl_3$ (20 mL). In another 25 mL of Schlenk tube, catalyst **Ru-III** (1.8 mg, 2 μmol) was dissolved in $CHCl_3$ (1 mL). After degassed in three freeze-vacuum-thaw cycles, the catalyst solution was then injected into the monomer solution via a syringe under vigorous stirring at 30 ^{o}C for 1 h, affording the corresponding poly[bis(norbornene pyrrolidine phenyl perylene bisimide)] **PTNP**$_{320}$ (270 mg, 99%). GPC: M_n = 107.2 kg mol^{-1}, PDI = 1.24.

Successive ROMP of BNP, TNP, and NP for synthesis of linear pentablock copolymers

A 100 mL of Schlenk tube was charged with bifunctional monomer **BNP** (27 mg, 0.02 mmol) dissolved in 20 mL of CH_2Cl_2. **Ru-III** (3.6 mg, 4 μmol), monomer **TNP** (275 mg, 0.64 mmol), and **NP** (22 mg, 0.02 mmol) were dissolved in 1 mL of CH_2Cl_2 in three 10 mL of Schlenk tubes, separately. After degassed with three freeze-vacuum-thaw cycles, **Ru-III** solution was injected into the **BNP** solution via a syringe under vigorous stirring at 30 ^{o}C for 0.5 h, and then **TNP** solution was injected into the reaction mixture and stirred for further 0.5 h. At last, the **NP** solution was injected into the reaction mixture and stirred for another 0.5 h, affording the linear pentablock copolymer poly[bis(norbornene pyrrolidine phenyl perylene bisimide)]-{*block*-poly[N-3,5-bis(trifluoromethyl)biphenyl-norbornene pyrrolidine]-*block*-poly[norbornene pyrrolidine phenyl perylene bisimide]}$_2$, *l*-**PBNP**$_{10}$-(*b*-**PTNP**$_{160}$)$_2$-(*b*-**PNP**$_5$)$_2$ (315.0 mg, 97%). ^{1}H NMR (500 MHz, $CDCl_3$): δ 8.74-8.58 (br, pery), 8.03-7.36 (br, CCCH + F_3CCCH + F_3CCCHCCF_3), 6.98-6.45 (br, NCC*H*CH + OCCH_2), 5.61-5.25 (br, *trans*-CH on PNBE backbone), 4.29-4.08 (br, CHCH_2O), 3.51-2.14 (br, CH_2NAr + =CHC*H*CH + =CHCHC*H* + NCH_3 + OCOCH_2), 2.02-1.17 (br, =CHCHCH_2 + OCOCH_2(CH_2)$_9$$CH_2$N). GPC: M_n = 127.9 kg mol^{-1}, PDI = 1.38.

Hydrolysis of cyclic polymer

c-[**PBNP**$_{10}$-(*b*-**PTNP**$_{160}$)$_2$-*b*-**PBNP**$_{10}$] (60 mg, 0.015 mmol of ester group) and KOH (8.4 mg, 0.15 mmol) dissolved in 10 mL of H_2O/dioxane (1:9 v/v). After reflux for 6 h, the mixture was concentrated and poured into an excess of acetone, and the hydrolyzed polymer **PTNP**$_{160}$-(*b*-**PHNP**$_{10}$)$_2$ was obtained. ^{1}H NMR (500 MHz, $CDCl_3$): 7.99-7.34 (br, CCCH + F_3CCCH + F_3CCCHCCF_3), 7.06-6.57 (br, NCC*H*CH), 5.64-5.23 (br, *trans*-CH on PNBE backbone), 3.56-2.57 (br, CH_2NAr + =CHC*H*CH + =CHCHC*H*), 2.11-1.27(br, =CHCHC*H*$_2$). GPC: M_n = 57.9 kg mol^{-1}, PDI = 1.33.

Synthesis of POSS propylisobutyl-1-carbethoxysemicarbazide

NH_2-POSS (8.74 g, 10 mmol) and THF (50 mL) were charged into a 1 L of round-bottom flask equipped with a magnetic stirrer under nitrogen atmosphere, and the mixture was stirred at -10 °C for 10 min. 4-Nitrophenyl chloroformate (4.02 g, 20 mmol) and triethylamine (6 mL) were added to the solution, and stirred for 30 min. Next, extra triethylamine (6 mL) was added, followed by a solution of ethyl carbazate (2.08 g, 20 mmol) in THF (20 mL). After stirring overnight at 40 °C, the mixture was concentrated in vacuum and purified by water and acetonitrile to give the product as a solid white powder (9.15 g, 9.1 mmol) in a yield of 91%. ^{1}H NMR (500 MHz, $CDCl_3$): δ 3.18 (t, 2H, NHC*H*$_2$), 1.93-1.81 (m, 7H, C*H*(CH_3)$_2$), 1.66-1.57 (m, 2H, CH_3C*H*$_2$O), 1.36-1.23 (m, 2H, SiCH_2C*H*$_2$), 0.98 (d, 24H, CH_3 + CH(C*H*$_3$)$_2$), 0.69-0.55 (m, 16H, SiCH_2). ^{13}C NMR (125 MHz, $CDCl_3$): δ 157.99, 43.05, 25.69, 23.87, 23.49, 22.47, 9.41.

Synthesis of POSS propylisobutyl-1,2,4-triazoline-3,5-diones (POSS-TAD)

POSS propylisobutyl-1-carbethoxysemicarbazide (9 g, 9 mmol) was dissolved in 1,4-dioxane solution (50 mL), then aqueous K_2CO_3 solution (4 mol L^{-1}, 20 mL) was added. The mixture was refluxed at 100 °C for 24 h, warm filtered, cooled to room temperature, and acidified until pH 1 by addition of hydrogen chloride. The combined solution was concentrated in vacuum, dissolved in dichloromethane, and dried with $MgSO_4$. The mixture was filtered off and concentrated in vacuum to give the product as light yellow waxy solid POSS propylisobutyl-1,2,4-urazole (7.9 g, 8.2 mmol) in a yield of 76%.

The POSS propylisobutyl-1,2,4-urazole (7.9 g, 8.2 mmol), trichloroisocyanuric aid (0.96 g, 4.12 mmol), and dichloromethane (50 mL) were charged into a 250 mL of round-bottom flask equipped with a magnetic stirrer under nitrogen atmosphere and stirred for 4 h in an ice-bath. The reaction mixture was filtered off and concentrated in vacuum. Then by dissolving in petroleum ether, centrifugation, and filtration, the filtrate was concentrated in vacuum to give the product as bright red crystals POSS

propylisobutyl-1,2,4-triazoline-3,5-dione (7.9 g, 8.2 mmol) in a yield of 99%. ^{1}H NMR (500 MHz, $CDCl_3$): δ 3.15 (t, 1H, NHCH_2), 1.94-1.82 (m, 7H, CH(CH_3)$_2$), 1.34-1.23 (m, 2H, NHCH_2CH_2), 0.94 (d, 21H, CH(CH_3)$_2$), 0.79-0.45 (m, 16H, SiCH_2). ^{13}C NMR (125 MHz, $CDCl_3$): δ 154.82, 44.55, 26.09, 25.53, 23.49, 24.41, 22.15.

Post-polymerization of cyclic polymer

c-[**PBNP**$_{10}$-(*b*-**PTNP**$_{160}$)$_2$-*b*-**PBNP**$_{10}$] (30 mg, 0.067 mmol of double bond) dissolved in 30 mL of $CHCl_3$ was added into the **POSS-TAD** (66.7 mg, 0.067 mmol of TAD group) solution in CH_2Cl_2. After 12 h reaction at room temperature, the mixture was concentrated and poured into an excess of CH_3OH, and the POSS-modified cyclic polymer *c*-[**PBNP**$_{10}$-(*b*-**PTNP**$_{20}$)$_2$-*b*-**PBNP**$_{10}$]-**POSS** was obtained. ^{1}H NMR (500 MHz, $CDCl_3$): δ 8.77-8.52 (br, pery), 8.05-7.32 (br, CCCH + F_3CCCH + F_3CCCHCCF_3), 7.05-6.33 (br, NCCHCH + OCCH_2), 5.59-5.24 (br, *trans*-CH on PNBE backbone), 4.34-4.06 (br, CHCH_2O), 3.62-3.47 (br, NCHCHPOSS), 3.46-0.52 (br, CH_2NAr + =CHCHCH + =CHCHCH + NCH_3 + OCOCH_2 + =CHCHCH_2 + OCOCH_2(CH_2)$_9$$CH_2$N + CH on POSS). ^{13}C NMR (125 MHz, $CDCl_3$): δ 174.72, 162.39, 148.59, 143.35, 142.79, 135.14, 132.58, 131.45, 128.08, 126.18, 124.49, 123.36, 122.35, 120.25, 119.15, 113.02, 50.02, 47.90, 46.77, 45.08, 41.15, 40.25, 37.60, 28.86, 25.79, 23.88, 22.37. GPC: M_n = 206.4 kg mol^{-1}, PDI = 1.45.

Post-polymerization of linear polymer

l-**PBNP**$_{20}$-(*b*-**PTNP**$_{160}$)$_2$ (30 mg, 0.067 mmol of double bond) dissolved in 30 mL of $CHCl_3$ was added into the **POSS-TAD** (66.7 mg, 0.067 mmol of TAD group) solution in CH_2Cl_2. After 12 h reaction at room temperature, the mixture was concentrated and poured into an excess of CH_3OH, and the POSS-modified linear polymer *l*-**PBNP**$_{20}$-(*b*-**PTNP**$_{20}$)$_2$-**POSS** was obtained. ^{1}H NMR (500 MHz, $CDCl_3$): δ 8.73-8.58 (br, pery), 8.01-7.26 (br, CCCH + F_3CCCH + F_3CCCHCCF_3), 7.12-6.64 (br, NCCHCH + OCCH_2), 5.72-5.30 (br, CH on PNBE backbone), 4.34-4.12 (br, CHCH_2O), 4.08-3.84 (br, NCHCHPOSS), 3.46-0.71 (br, CH_2NAr + =CHCHCH + =CHCHCH + NCH_3 + OCOCH_2 + =CHCHCH_2 + OCOCH_2(CH_2)$_9$$CH_2$N + CH on POSS). ^{13}C NMR (125 MHz, $CDCl_3$): δ 175.12, 162.18, 141.58, 135.69, 132.45, 132.24, 132.02, 131.15, 128.08, 129.03, 128.48, 126.22, 124.53, 123.40, 122.28, 120.59, 119.47, 113.28, 49.98, 47.72 46.77, 45.60, 41.06, 37.99, 36.47, 34.25, 29.86, 27.67, 23.69. GPC: M_n = 265.2 kg mol^{-1}, PDI = 1.40.

Supplementary Discussion

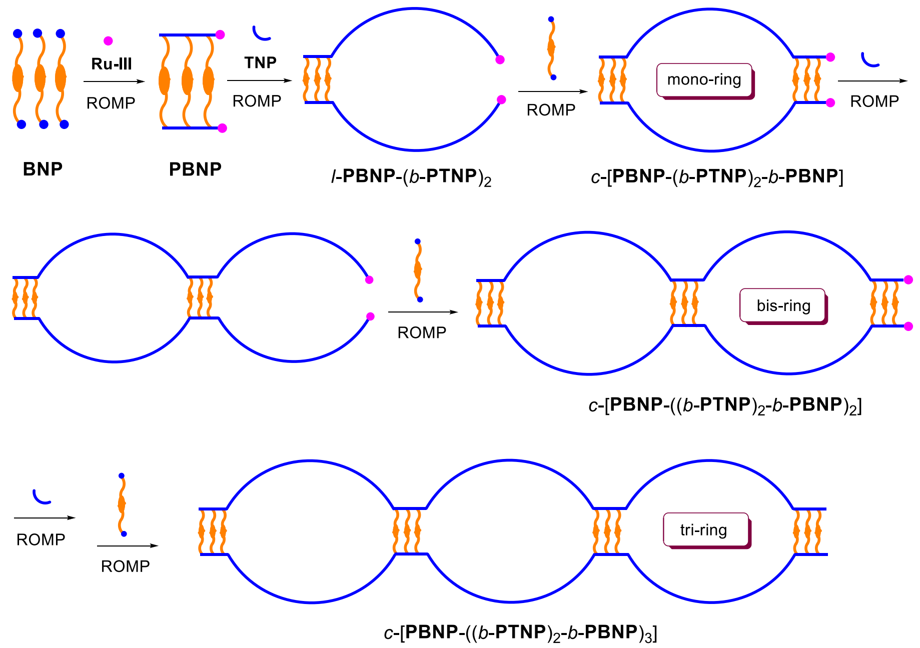

Supplementary Figure 1. The cartoon representation for blocking-cyclization process.

Supplementary Note 1. Characteristics of linear and monocyclic polymers

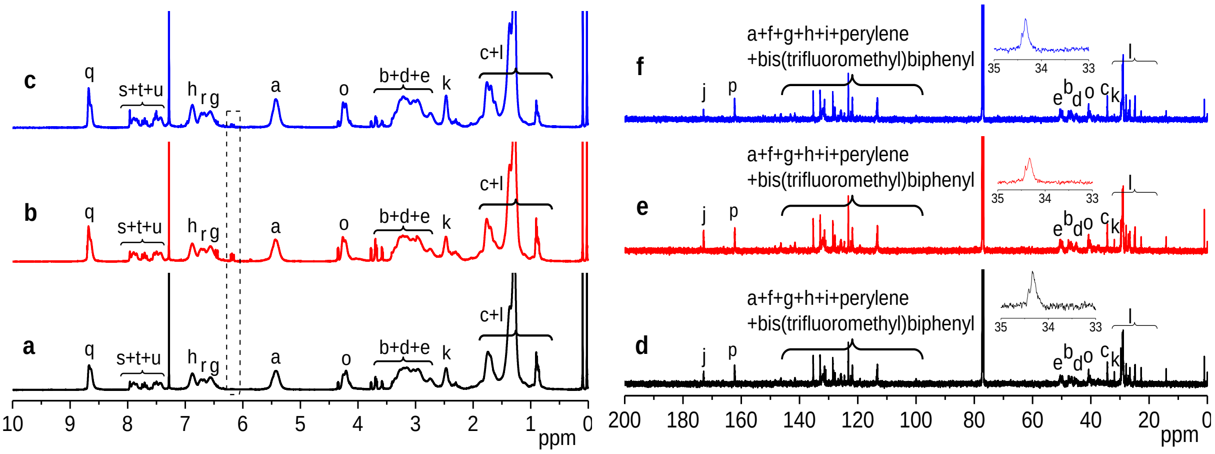

Supplementary Figure 2. ^{1}H (a-c) and ^{13}C (d-f) NMR spectra of ***l*-PBNP$_{40}$-(*b*-PTNP$_{20}$)$_2$** (a,d) and *c*-[**PBNP$_{20}$-(*b*-PTNP$_{20}$)$_2$-*b*-PBNP$_{20}$**] (b,e and c,f) in $CDCl_3$.

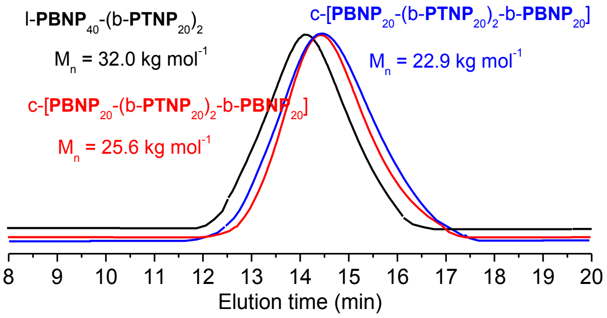

Supplementary Figure 3. GPC traces of linear and monocyclic block copolymers.

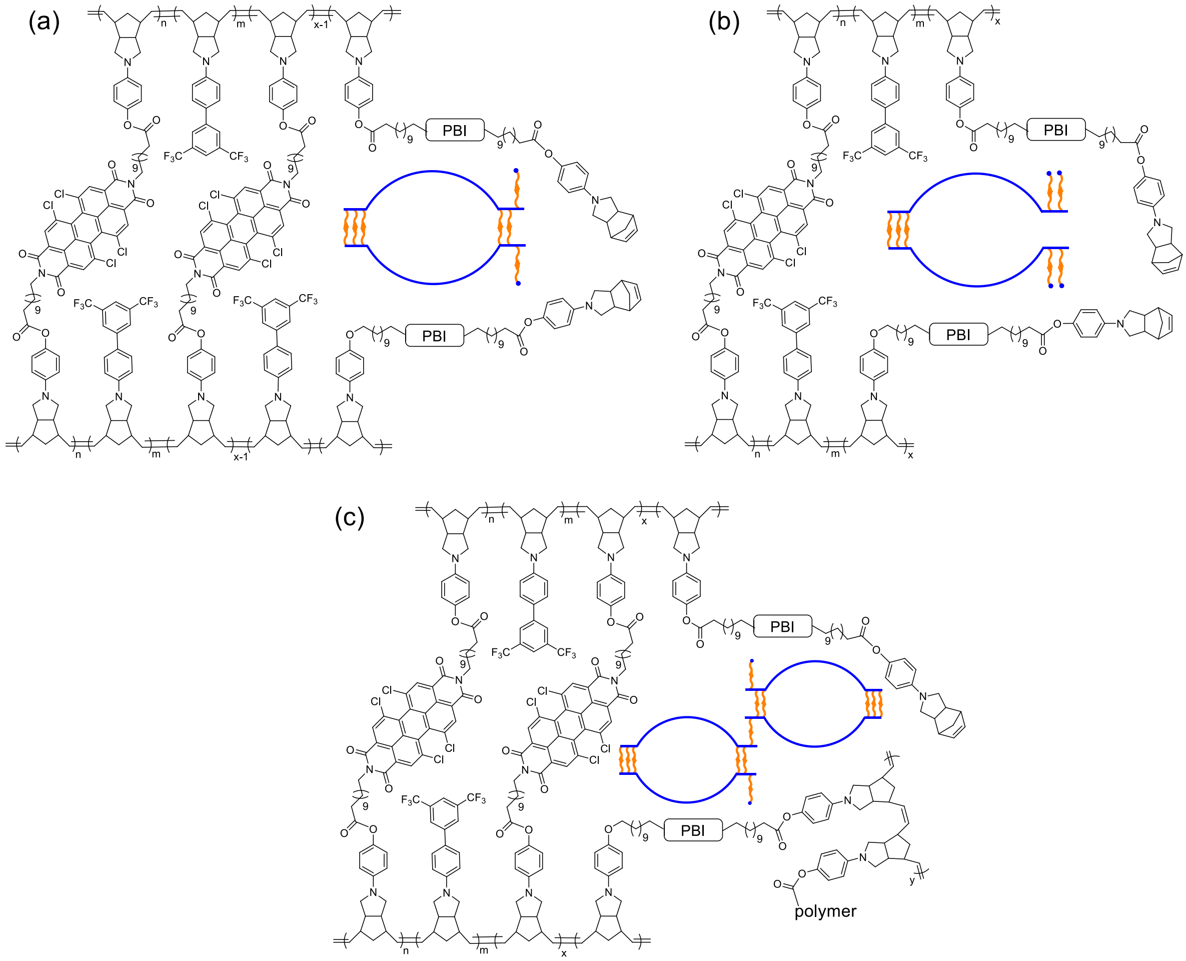

Supplementary Figure 4. Possible defective cyclic structure and cartoon representation of cyclic polymer with little unreacted norbornene groups (a). Cartoon representations for impossible structures (b,c).

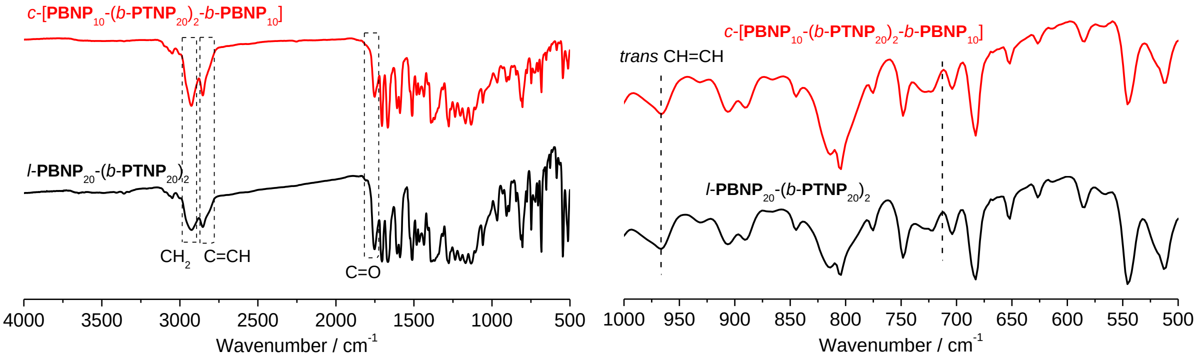

Supplementary Figure 5. IR spectra of *l*-**PBNP**$_{20}$-(*b*-**PTNP**$_{20}$)$_2$ and *c*-[**PBNP**$_{10}$-(*b*-**PTNP**$_{20}$)$_2$-*b*-**PBNP**$_{10}$].

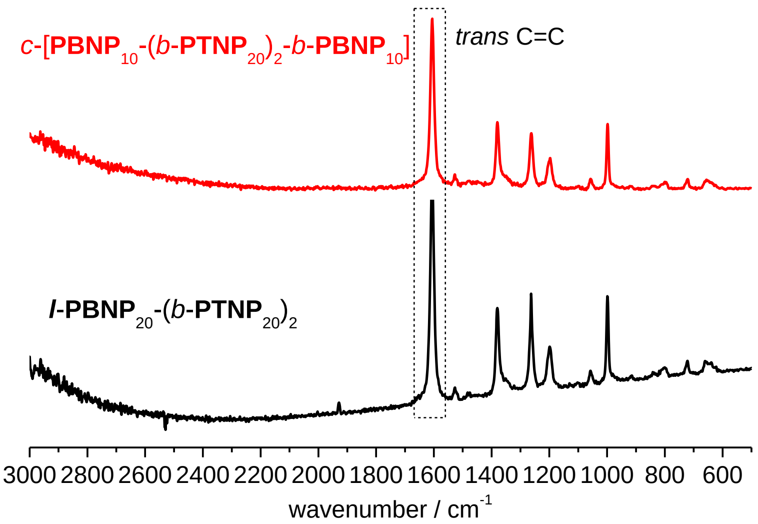

Supplementary Figure 6. Raman spectra of linear and monocyclic copolymers.

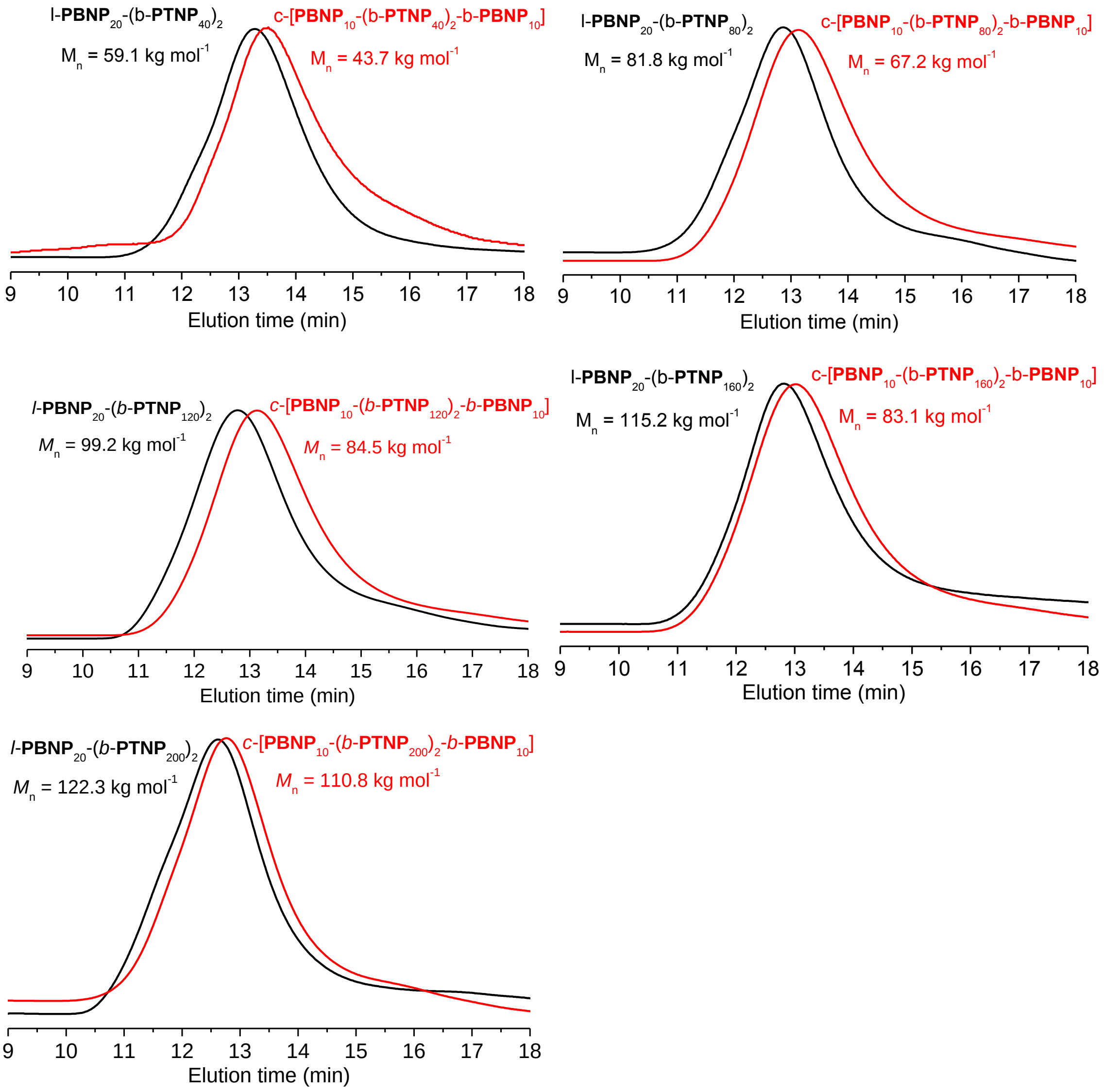

Supplementary Figure 7. GPC traces of linear and monocyclic block copolymers.

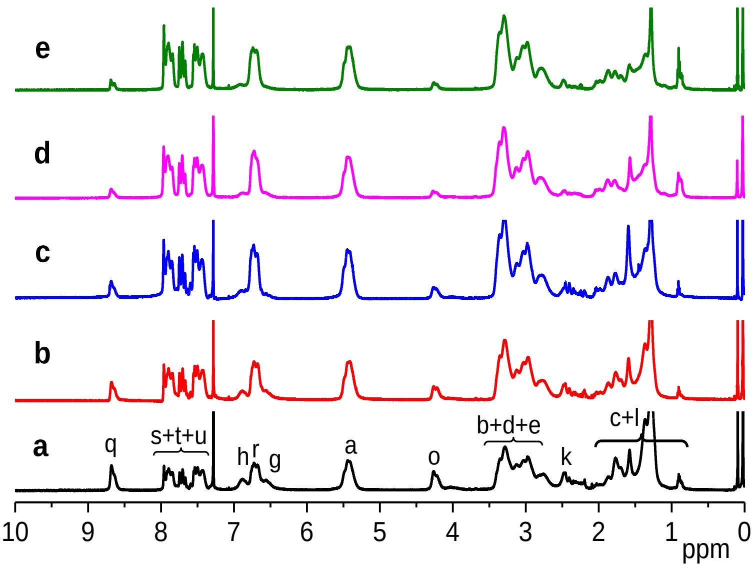

Supplementary Figure 8. ^{1}H NMR spectra of *c*-[**PBNP**$_{10}$-(*b*-**PTNP**$_{40}$)$_2$-*b*-**PBNP**$_{10}$] (a), *c*-[**PBNP**$_{10}$-(*b*-**PTNP**$_{80}$)$_2$-*b*-**PBNP**$_{10}$] (b), *c*-[**PBNP**$_{10}$-(*b*-**PTNP**$_{120}$)$_2$-*b*-**PBNP**$_{10}$] (c), *c*-[**PBNP**$_{10}$-(*b*-**PTNP**$_{160}$)$_2$-*b*-**PBNP**$_{10}$] (d), and *c*-[**PBNP**$_{10}$-(*b*-**PTNP**$_{200}$)$_2$-*b*-**PBNP**$_{10}$] (e) in $CDCl_3$.

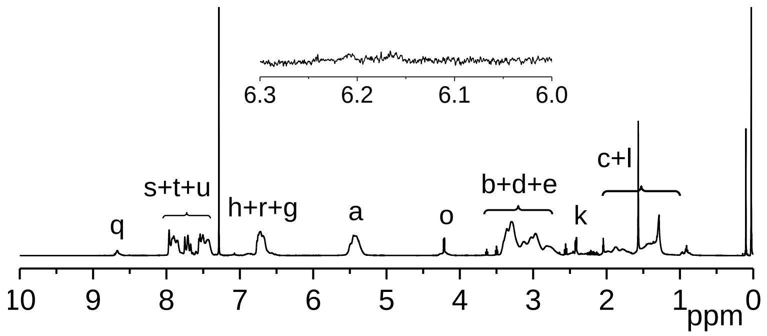

Supplementary Figure 9. ^{1}H NMR spectrum of *c*-[**PBNP**$_{10}$-(*b*-**PTNP**$_{160}$)$_2$-*b*-**PBNP**$_{10}$] when the coherent accumulation number added up to 5000.

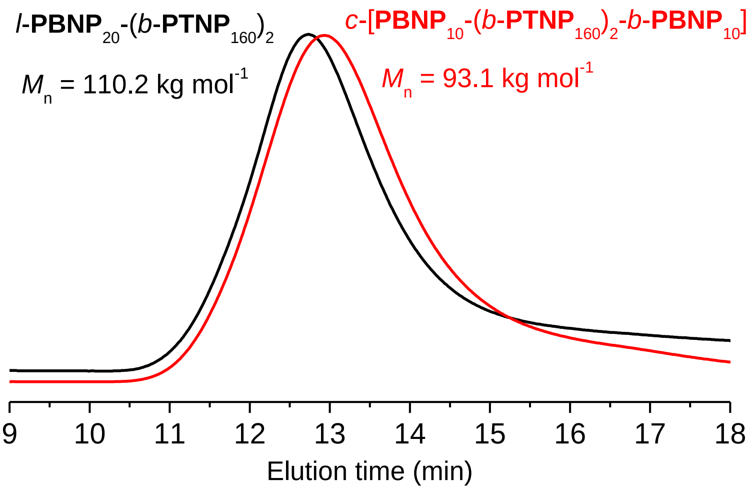

Supplementary Figure 10. GPC traces of linear and monocyclic block copolymers prepared at -20 ^{o}C.

Supplementary Note 2. More insight into the ladderphane and the rigid single-stranded polymer structure

The well-defined ladderphane structure was obtained under the optimized reaction conditions mentioned above, while the ill-defined structure may result in unexpected or complicated polymer structures. Therefore, the difference in polymer feature with or without the well-defined ladderphane structure will play a vital role in successful or failed synthesis of cyclic polymer. For comparison, several homopolymers and copolymers were prepared, and the reaction conditions and polymer characteristics were listed in Supplementary Table 1.

Relevance of initial difunctional monomer concentration to the ladderphane structure and polydispersity of polymers. The contribution of initial monomer concentration to the formation of well-defined ladderphane structure and the relatively broad PDI of polymers was evaluated. Firstly, the homopolymer **PTNP**$_{320}$ was synthesized by ROMP of monofunctional **TNP** at the conventional concentration of 3.2×10^{-2} mol L^{-1} (Supplementary Section 1), although the electron-donating effect of pyrrolidine moiety in **TNP** increased polymerization activity in comparison to the NBE imide monomer to broaden the PDI of polymers,[4-6] while **PTNP**$_{320}$ exhibited a lower M_n of 107.2 kg mol^{-1} and a narrower PDI of 1.24 (Supplementary Fig. 11) compared to those (M_n = 115.2 kg mol^{-1}, PDI = 1.43, Table 1) of *l*-**PBNP**$_{20}$-(*b*-**PTNP**$_{160}$)$_2$ with the same **PTNP** units, suggesting that the broad PDI of copolymers was not caused mainly by the monofunctional **TNP** but the difunctional **BNP** during polymerization. Then, ROMP of difunctional **BNP** under varied conditions was investigated to reveal the influence of ladderphane **PBNP** structure on the PDI value of copolymers. Two ladderphanes **PBNP**$_{10}$ and **PBNP**$_{20}$ were achieved at the feed ratios ([**BNP**]/[Cat]) of 5 and 10, respectively, under a low concentration of 1×10^{-3} mol L^{-1} in CH_2Cl_2, and the measured M_ns of 8.3 and 17.1 kg mol^{-1} from their GPC curves (Supplementary Fig. 12a) were less than the theoretical values of 13.4 and 26.8 kg mol^{-1}. As predicted, the ladderphanes **PBNP**$_{10}$ and **PBNP**$_{20}$ displayed the broader PDIs of 1.40 and 1.42, respectively, compared with the single-stranded **PTNP**$_{320}$. Therefore, the broad PDI of copolymers was actually contributed from the ladderphane **PBNP** segment, which were also observed in some other ladderphane polymers (PDI ~ 1.5).[7-9] According to the report in literature,[10] the PBI-functionalized di(metacrylic acid ester) monomers could be regularly arranged in solution by the strong π-π interaction between the PBI moieties, and the monomers preferred to form the polymer with stable double-stranded structure rather than cross-linking structure in the process of *in situ* polymerization. Similarly, the difunctional

BNP in diluted solution might form a large number of separated cluster of regularly arranged monomers by the π-π stacking of PBI linker in **BNP**, and the difference of the monomer numbers in each cluster possibly resulted in the broad PDI. It was found that the M_n value of **PBNP** had exactly doubled as the feed ratio increased from 5 to 10, and the ^{1}H NMR spectrum of **PBNP**$_{20}$ (Supplementary Fig. 13) showed that the signal of olefinic protons on the NBE ring at 6.2 ppm was disappeared completely after polymerization, indicating that ROMP of difunctional **BNP** was well-controllable and there was no unreacted NBE unit left in **PBNP**$_{20}$. By virtue of the peak area ratio of double-bond protons (S_a) on the PNBE backbone at 5.59-5.24 ppm to that of the end phenyl group (S_{Ph}) at 7.26-7.15 ppm (Supplementary Fig. 13), the degree of polymerization [DP = $(S_a/2)/(S_{Ph}/5)$] was calculated as about 18, which was highly close to the theoretical value of 20 according to the feed ratio, indirectly proved that ladderphane **PBNP** has a predicted double-stranded structure. Furthermore, if the initial concentration of difunctional **BNP** doubled to 5×10^{-2} mol L^{-1}, although the feed ratio ([**BNP**]/[Cat]) of 5 was unchanged, the obtained **PBNP**$_n$ possesses an increased M_n of 18.9 kg mol^{-1} and a broader PDI of 1.43, and the GPC curve showed a shoulder peak (Supplementary Fig. 12a), likely due to the branched structure in polymer (Supplementary Fig. 14) formed at this higher monomer concentration. **PBNP**$_{20}$ with the well-defined ladderphane structure was selected as a representative for the measurement of its absolute molecular weight ($M_{MALDI\text{-}TOF}$) by MALDI-TOF MS, as shown in Supplementary Fig. 15. The peaks were separated by 1356.53 (m + Na) mass unit, which corresponded to the $M_{\mathbf{BNP}}$ of 1333.50 for each monomer unit. The $M_{MALDI\text{-}TOF}$ of **PBNP**$_{20}$ was 21.79 kg mol^{-1}, and the calculated DP was about 17, which was in good agreement with the DP of 18 determined by ^{1}H NMR analysis. The $M_{MALDI\text{-}TOF}$ value was obviously higher than the M_n value of **PBNP**$_{20}$ by GPC testing, implying the depressed hydrodynamic volume of ladderphane structure.

Lastly, as the monofunctional **TNP** was added into the high concentration reaction system of **PBNP**$_n$ bearing multiple propagating carbenes (Supplementary Fig. 14), the resulting copolymer **PBNP**$_n$-(*b*-**PTNP**$_{160}$)$_m$ presented a larger M_n of 151.4 kg mol^{-1} and more broader PDI of 1.64 than those (M_n = 108.9 kg mol^{-1}, PDI = 1.33) of *l*-**PBNP**$_{10}$-(*b*-**PTNP**$_{160}$)$_2$ obtained under a lower **BNP** concentration of 1×10^{-3} mol L^{-1}, while the GPC curve still showed a shoulder peak (Supplementary Fig. 12b), which also proved that the initial **PBNP**$_n$ segment has primarily branched structure. When the difunctional **BNP** was added again to the solution of **PBNP**$_n$-(*b*-**PTNP**$_{160}$)$_m$ bearing multiple propagating carbenes, the generated copolymer

PBNP$_n$-(*b*-**PTNP**$_{160}$)$_m$-*b*-**PBNP**$_x$ has a further increased M_n of 219.3 kg mol^{-1} and wide PDI of 2.23, and obviously the GPC curve showed two distinct peaks (Supplementary Fig. 12b) corresponding to the M_ns of 745.1 and 183.2 kg mol^{-1}, respectively, indicating that the polymerization produced copolymers with highly branched or slightly cross-linked structures. From these results, it could be concluded that the lower initial difunctional **BNP** concentration ($\leq 1\times10^{-3}$ mol L^{-1}) has an important contribution to the formation of the first ladderphane **PBNP** segment with well-defined structure, which would play a key role in preparing the linear triblock copolymer *l*-**PBNP**-(*b*-**PTNP**)$_2$ and thus the derived cyclic copolymer *c*-**PBNP**-(*b*-**PTNP**)$_2$-*b*-**PBNP**.

Influence of U shape on the hydrodynamic volume of triblock copolymers. The linear triblock copolymer *l*-**PBNP**$_{20}$-(*b*-**PTNP**$_{160}$)$_2$ might adopt U shape, ascribed to its chemical structure features of one short rigid ladderphane **PBNP** segment and two long flexible single-stranded **PTNP** segments. When the ladderphane **PBNP** segment in *l*-**PBNP**$_{20}$-(*b*-**PTNP**$_{160}$)$_2$ was wholly or half replaced with the single-stranded **PNP** segment bearing the PBI pendants, a linear triblock copolymer *l*-**PTNP**$_{160}$-*b*-**PNP**$_{20}$-*b*-**PTNP**$_{160}$ ([**TNP**]:[**NP**]:[**TNP**]:[Cat] = 160:20:160:1, [**TNP**]$_0$ = 3.2×10^{-2} mol L^{-1}) or a pentablock copolymer *l*-**PBNP**$_{10}$-(*b*-**PNP**$_5$)$_2$-(*b*-**PTNP**$_{160}$)$_2$ ([**BNP**]:[**NP**]:[**TNP**]:[Cat] = 5:5:160:1, [**BNP**]$_0$ = 1×10^{-3} mol L^{-1}) was yielded by ROMP of **TNP** and **NP** (Supplementary Figs 16 and 17) or **BNP**, **NP**, and **TNP** with the corresponding feed ratios, which displayed a higher M_n of 136.1 or 126.4 kg mol^{-1} and a narrower PDI of 1.27 or 1.36 (Supplementary Fig. 18a) than those (M_n = 115.2 kg mol^{-1}, PDI = 1.42, Table 1) of *l*-**PBNP**$_{20}$-(*b*-**PTNP**$_{160}$)$_2$, suggesting that the ladderphane-contained *l*-**PBNP**$_{20}$-(*b*-**PTNP**$_{160}$)$_2$ in "U" shape could reduce the hydrodynamic volume or lowered the M_n value (even $M_{\mathbf{BNP}}$ (1333.50 g mol^{-1}) > $M_{\mathbf{NP}}$ (1035.34 g mol^{-1})) and broaden the PDI value. From the M_n and PDI values of *l*-**PBNP**$_{20}$-(*b*-**PTNP**$_{160}$)$_2$, *l*-**PBNP**$_{10}$-(*b*-**PNP**$_5$)$_2$-(*b*-**PTNP**$_{160}$)$_2$, and *l*-**PTNP**$_{160}$-*b*-**PNP**$_{20}$-*b*-**PTNP**$_{160}$, it was deduced that the higher DP of ladderphane **PBNP** segment is, the lower hydrodynamic volume or M_n value, and the broader PDI of copolymer. By changing the adding sequence of **TNP** and **NP** in polymerization, the resulted another pentablock copolymer *l*-**PBNP**$_{10}$-(*b*-**PTNP**$_{160}$)$_2$-(*b*-**PNP**$_5$)$_2$ has a much larger M_n of 127.9 kg mol^{-1} and the same PDI of 1.38 compared to those (M_n = 96.8 kg mol^{-1}, PDI = 1.38, Table 1) of *c*-[**PBNP**$_{10}$-(*b*-**PTNP**$_{160}$)$_2$-*b*-**PBNP**$_{10}$], because the second ladderphane **PBNP** segment was replaced with two single-stranded **PNP** segments and thus lost the cyclic structure. Intuitively, *l*-**PBNP**$_{10}$-(*b*-**PNP**$_5$)$_2$-(*b*-**PTNP**$_{160}$)$_2$ has nearly the same M_n and PDI values as

l-**PBNP**$_{10}$-(*b*-**PTNP**$_{160}$)$_2$-(*b*-**PNP**$_5$)$_2$ on the basis of their almost overlapped GPC curves (Supplementary Fig. 18a), indicating that the sequence of single-stranded **PNP** and **PTNP** blocks in copolymer did not affect the hydrodynamic volume. According to the feed ratio, the theoretical block ratio of (**PBNP**+**PNP**)/**PTNP** in *l*-**PBNP**$_{10}$-(*b*-**PTNP**$_{160}$)$_2$-(*b*-**PNP**$_5$)$_2$ should be (10+10):320. By integrating the peak signals related to the aromatic protons (H_q) on PBI linkers at 8.72-8.54 ppm and those on bis(trifluoromethyl)biphenyl (H_{s+t+u}) at 8.09-7.34 ppm in ^{1}H NMR spectrum (Supplementary Fig. 19), the actual (**PBNP**+**PNP**)/**PTNP** ratio was calculated to be 24:317.1, which was almost consistent with the theoretical block ratio.

Influence of second ladderphane structure on cyclic polymer topology. Instead of the rigid PBI-linked difunctional **BNP** for preparation of second ladderphane segment as cyclizing unit, while the flexible linker-contained difunctional **BNI** (Supplementary Figs 16 and 20) was added into the polymerizing system and further propagated on the living linear intermediate *l*-**PBNP**$_{10}$-(*b*-**PTNP**$_{160}$)$_2$, it was astonishingly to find that not a cyclic but actually a linear copolymer *l*-**PBNP**$_{10}$-(*b*-**PTNP**$_{160}$)$_2$-(*b*-**PBNI**$_5$)$_2$ was generated, judged by the GPC traces as depicted in Supplementary Fig. 18b. This copolymer has a larger M_n of 114.1 kg mol^{-1} and broader PDI of 1.38 than those (M_n = 108.9 kg mol^{-1}, PDI = 1.33) of linear intermediate *l*-**PBNP**$_{10}$-(*b*-**PTNP**$_{160}$)$_2$, and importantly these enlarged M_n and PDI values were in contrast with those of cyclic polymer, because the M_n of cyclic polymer should be less than that of linear polymer ($M_{n,c} < M_{n,l}$), suggesting that the **PBNI** segment-contained copolymer was not a cyclic topology, or the cyclization was not conducted at all. Besides, the GPC trace (Supplementary Fig. 18b) showed a shoulder peak with M_n of about 321.7 kg mol^{-1}, indicating the branched (Supplementary Fig. 16) or other complicated structures[11,12] existed in such copolymer from the flexible linker-contained difunctional **BNI**, although no precipitate has been observed in polymerization process. The observed result was a strong evidence to support that the second ladderphane **PBNP** containing the rigid PBI linkers indeed played a crucial role in successful preparation of cyclic polymer via blocking-cyclization technique, due to the regular arrangement of difunctional **BNP** monomers in solution[10] for well-controlled polymerization.

Influence of the rigid single-stranded polymer chain bearing large aryl pendants on the hydrodynamic volume. According to the reports, the hydrodynamic volume of cyclic polyacetylene with rigid backbone[13] and cyclic poly(4-vinylbenzyl-carbazole) bearing rigid aromatic pendants[14] were smaller than those of the linear analogues. In order to verify the influence of the relatively rigid N-arylpyrrolidine-attached PNBE chains on the hydrodynamic volume, the monofunctional **TNP** was replaced by **NP** bearing more rigid and larger PBI-R

pendants for preparing the linear *l*-**PBNP**$_{20}$-(*b*-**PNP**$_{120}$)$_2$ and its cyclic counterpart *c*-[**PBNP**$_{10}$-(*b*-**PNP**$_{120}$)$_2$-*b*-**PBNP**$_{10}$] (Supplementary Fig. 21) with the feed ratios ([**BNP**]:[**NP**]:[**BNP**]:[Cat]) of 10:120:0:1 and 5:120:5:1 (entries 14 and 15, Supplementary Table 1), respectively, and the corresponding GPC curves provided the dominating peaks (97%) with the M_ns of 125.2 and 109.0 kg mol^{-1} for linear and cyclic copolymers (Supplementary Fig. 22a), demonstrating that the cyclic copolymer had a smaller hydrodynamic volume in comparison to the linear analogue. Therefore, the hydrodynamic volume was still a useful evidence to distinguish between linear and cyclic polymers with the rigid PNBE main chain. Besides, the GPC curves showed another small peak (3%) with the M_n of 1.08 kg mol^{-1} for unreacted monomer. This was likely due to the adverse steric hindrance effect of large PBI-R pendants on **PNP** chain, which inhibited the chain growth during polymerization as the degree of polymerization reached a critical value. The ^{1}H NMR spectrum of *c*-[**PBNP**$_{10}$-(*b*-**PNP**$_{120}$)$_2$-*b*-**PBNP**$_{10}$] showed a weak signal of the residual olefinic protons on the NBE ring at 6.14 ppm (Supplementary Fig. 23a). By integrating the ^{1}H NMR signal of olefinic protons at 6.14 ppm and that on the main chain of PNBE at 5.63-5.25 ppm (H_a), the molar ratio was calculated to be about 1:40, which was nearly consistent with that by GPC analysis, indicating that the signal of double bonds on the NBE rings came from the residual monomer **NP** rather than the unreacted NBE rings in the copolymers.

In order to enable the monofunctional **NP** to fully react, the [**NP**]/[Cat] ratio decreased to 80 (entries 12 and 13, Supplementary Table 1), and the signal of unreacted monomers was disappeared completely in the GPC curves (Supplementary Fig. 22b) and ^{1}H NMR spectrum (Supplementary Fig. 23b). The M_n of 89.2 kg mol^{-1} for *c*-[**PBNP**$_{10}$-(*b*-**PNP**$_{80}$)$_2$-*b*-**PBNP**$_{10}$] was lower than that of 106.1 kg mol^{-1} for linear counterpart *l*-**PBNP**$_{20}$-(*b*-**PNP**$_{80}$)$_2$. The PDIs of 1.33 and 1.29 for *l*-**PBNP**$_{20}$-(*b*-**PNP**$_{80}$)$_2$ and *c*-[**PBNP**$_{10}$-(*b*-**PNP**$_{80}$)$_2$-*b*-**PBNP**$_{10}$] were narrowed by comparing with those of 1.45 and 1.43 for *l*-**PBNP**$_{20}$-(*b*-**PTNP**$_{80}$)$_2$ and *c*-[**PBNP**$_{10}$-(*b*-**PTNP**$_{80}$)$_2$-*b*-**PBNP**$_{10}$] bearing the biphenyl pendants on **PTNP** chain, respectively, because the stronger π-π stacking of large PBI-R pendants preferred to bring two linear **PNP** chains closer together, and thus the two end groups of linear *l*-**PBNP**$_{20}$-(*b*-**PNP**$_{80}$)$_2$ should be in close proximity to each other to promote its cyclization. As a result, the large PBI-R pendant would have an opposite effect in the blocking-cyclization process, it was on the one hand helpful for cyclizing linear intermediate, and on the other hand, too large pendant was in disfavor of the formation of long polymer chain to get the large cyclic structure. Therefore, the size of pendants on the single-stranded chain should be optimized.

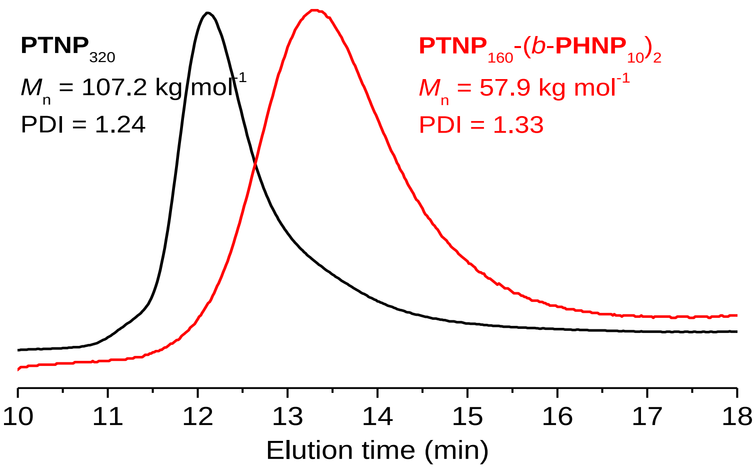

Supplementary Figure 11. GPC traces of homopolymer (black) and the hydrolyzed copolymer (red) from *c*-[**PBNP**$_{10}$-(*b*-**PTNP**$_{160}$)$_2$-*b*-**PBNP**$_{10}$].

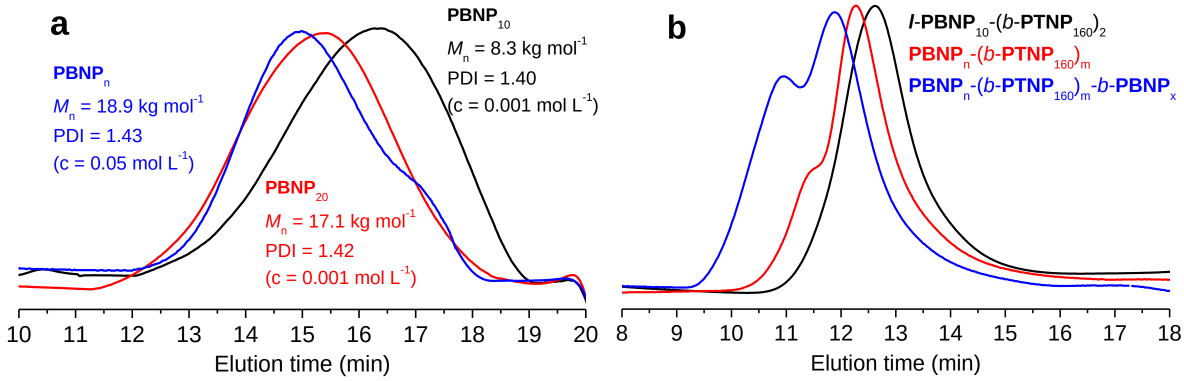

Supplementary Figure 12. GPC traces of homopolymers (a) and copolymers (b).

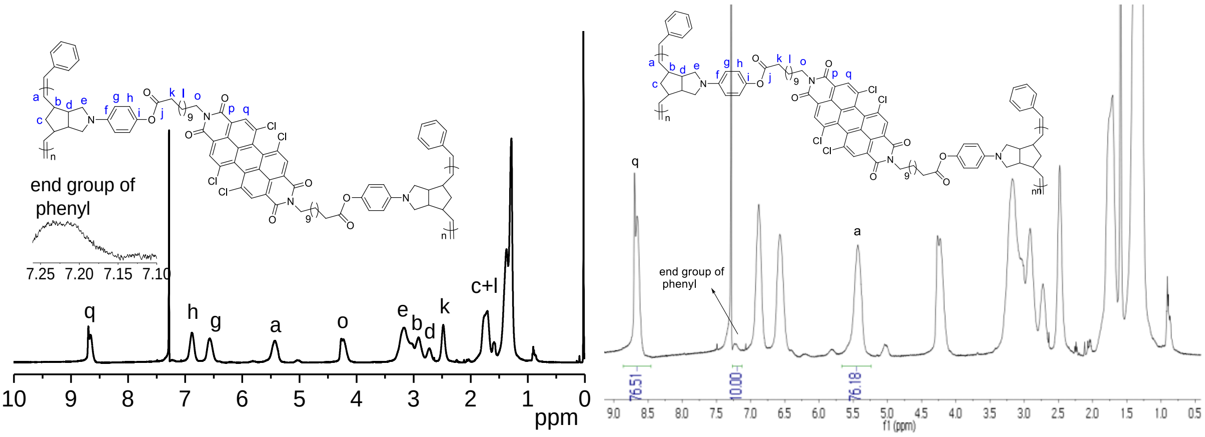

Supplementary Figure 13. ^{1}H NMR spectra of **PBNP**$_{20}$ in $CDCl_3$.

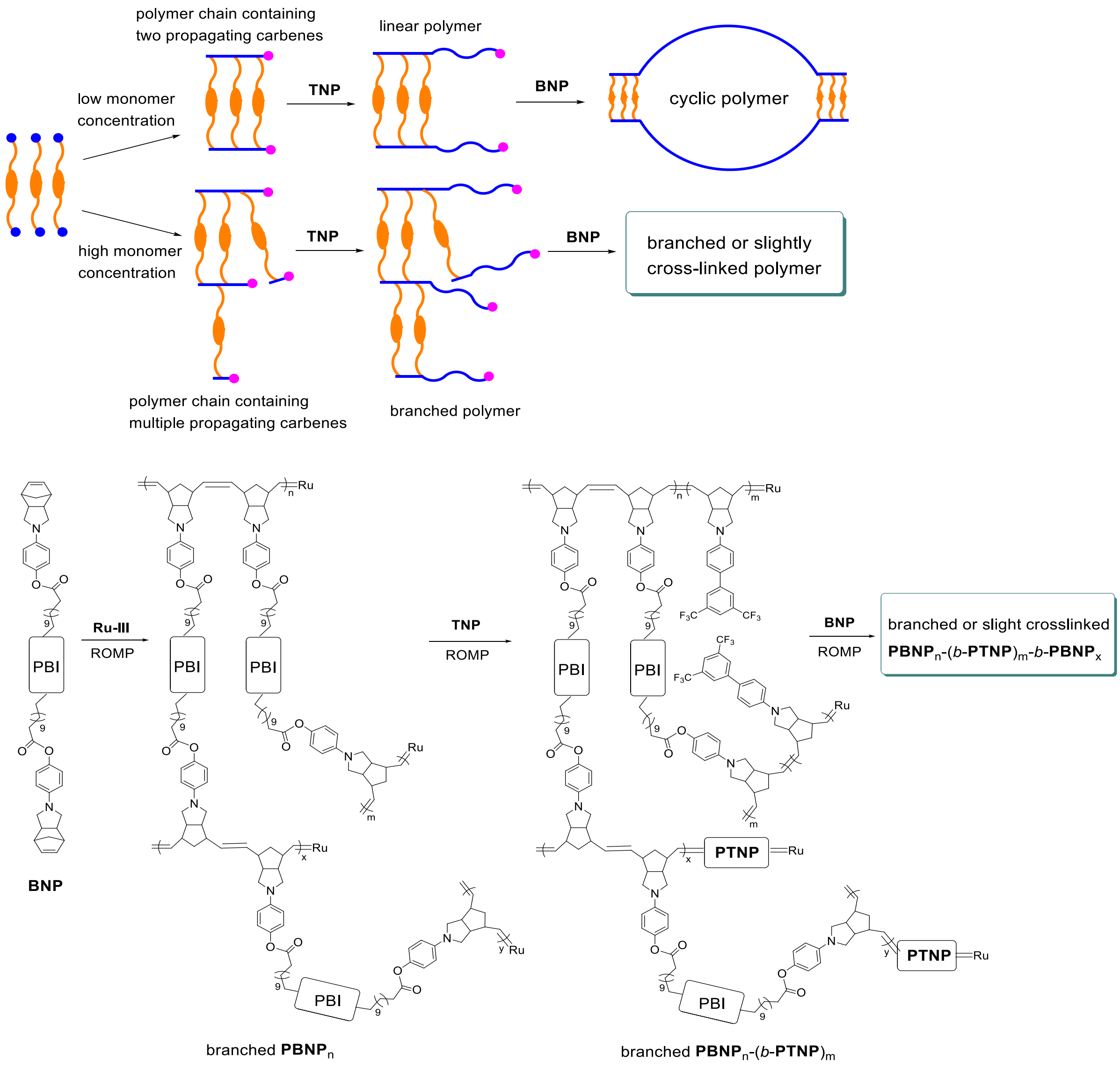

Supplementary Figure 14. Cartoon representations for comparison of polymer structures obtained from different initial monomer concentration (top) and the schematic diagram of branched or slightly cross-linked polymers formed at high monomer concentration of 0.05 mol L^{-1} (down).

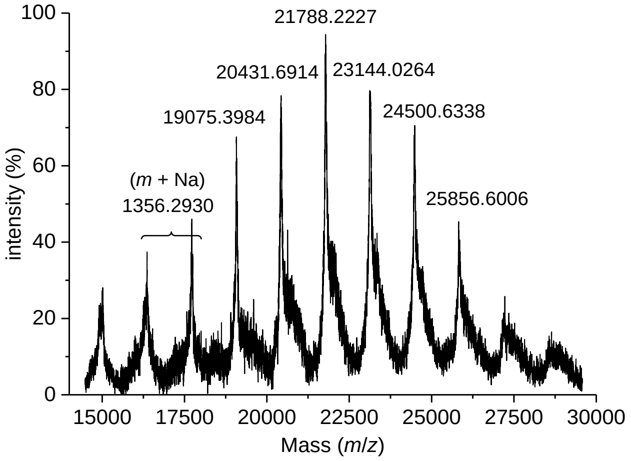

Supplementary Figure 15 MALDI-TOF MS of **PBNP**$_{20}$.

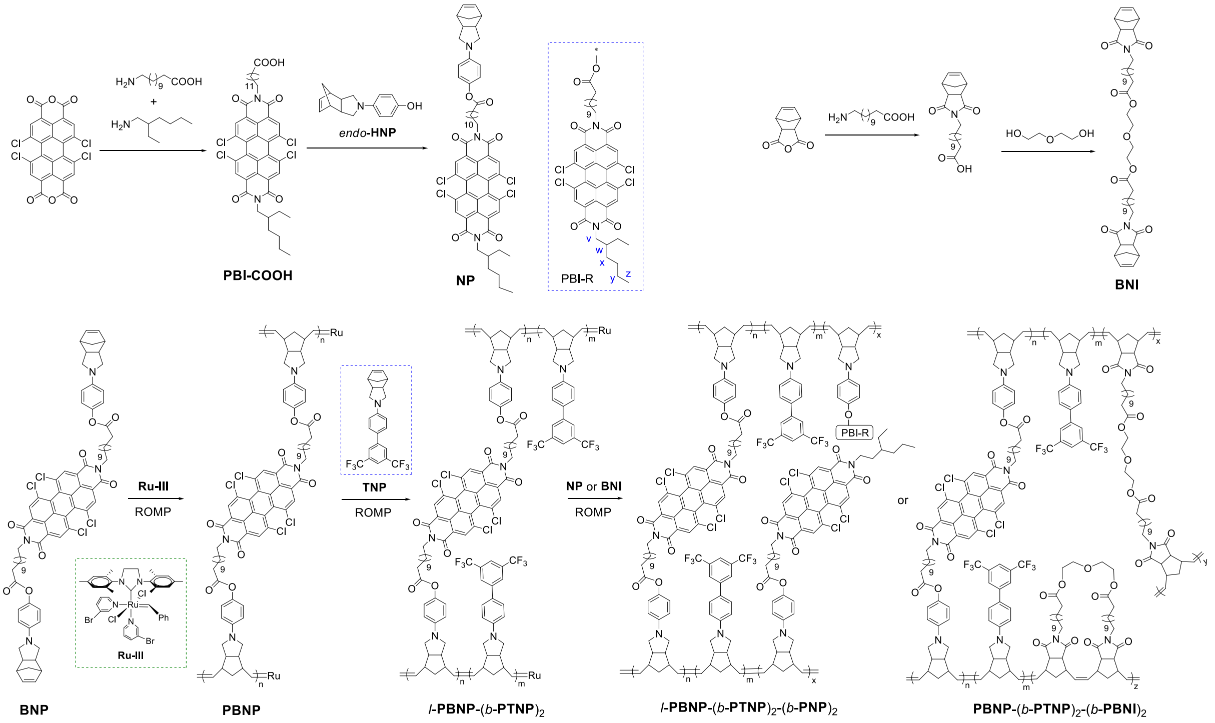

Supplementary Figure 16. Syntheses of PBI-R pendant or flexible linker-contained monomers and polymers.

Supplementary Table 1 Characteristics for polymers[a]

Entry	Polymer	[BNP]:[NP]:[TNP]:[BNP]:[NP]:[BNI]:[TNP]:[Cat][b]	[PBNP+PNP]/[PTNP][c]	M_n[d] (kg mol^{-1})	PDI[d]	Yield (%)
1[e]	**PTNP**$_{320}$	0:0:320:0:0:0:0:1	/	107.2	1.24	99
2[f]	**PBNP**$_{10}$	5:0:0:0:0:0:0:1	/	8.3	1.40	98
3[f]	**PBNP**$_{20}$	10:0:0:0:0:0:0:1	/	17.1	1.42	99
4[g]	**PBNP**$_n$	5:0:0:0:0:0:0:1	/	18.9	1.43	97
5[g]	**PBNP**$_n$-(b-**PTNP**$_{160}$)$_m$	5:0:160:0:0:0:0:1	/	151.4	1.64	98
6[g]	**PBNP**$_n$-(b-**PTNP**$_{160}$)$_m$-b-**PBNP**$_x$	5:0:160:5:0:0:0:1	/	219.3	2.23	94
7[e]	l-**PTNP**$_{160}$-b-**PNP**$_{20}$-b-**PTNP**$_{160}$	0:0:160:0:20:0:160:1	/	136.1	1.27	93
8[f]	l-**PBNP**$_{10}$-(b-**PNP**$_5$)$_2$-(b-**PTNP**$_{160}$)$_2$	5:5:160:0:0:0:0:1	/	126.4	1.36	95
9[f]	l-**PBNP**$_{10}$-(b-**PTNP**$_{160}$)$_2$-(b-**PNP**$_5$)$_2$	5:0:160:0:5:0:0:1	24:317.1	127.9	1.38	97
10[f]	l-**PBNP**$_{10}$-(b-**PTNP**$_{160}$)$_2$	5:0:160:0:0:0:0:1	/	108.9	1.33	95
11[f]	l-**PBNP**$_{10}$-(b-**PTNP**$_{160}$)$_2$-(b-**PBNI**$_5$)$_2$	5:0:160:0:0:5:0:1	/	114.1	1.38	98
12[h]	l-**PBNP**$_{20}$-(b-**PNP**$_{80}$)$_2$	10:80:0:0:0:0:0:1	/	106.1	1.33	94
13[i]	c-[**PBNP**$_{10}$-(b-**PNP**$_{80}$)$_2$-b-**PBNP**$_{10}$]	5:80:0:5:0:0:0:1	/	89.2	1.29	92
14[h]	l-**PBNP**$_{20}$-(b-**PNP**$_{120}$)$_2$	10:120:0:0:0:0:0:1	/	125.2	1.34	85
15[i]	c-[**PBNP**$_{10}$-(b-**PNP**$_{120}$)$_2$-b-**PBNP**$_{10}$]	5:120:0:5:0:0:0:1	/	109.0	1.31	81

[a] Polymerization conditions: using **Ru-III** as catalyst, CH_2Cl_2 as solvent, temperature = 30 ^oC. Polymerization time: 60 min for entries 1-4, (30+30 min) for entries 5, 10, 12, and 14, and (30+30+30 min) for entries 6-9, 11, 13, and 15.

[b] The feed ratios in of monomers to catalyst for polymerization of di- and monofunctional monomers in sequential addition manner

[c] The block ratio of **PBNP** to **PTNP** by ^{1}H NMR spectroscopy analysis
[d] Determined by GPC in THF relative to monodispersed polystyrene standards
[e] [**TNP**]$_0$ = 0.032 mol mL^{-1} refers to the initial monomer concentration of **TNP**
[f] [**BNP**]$_0$ = 0.001 mol L^{-1} refers to the initial monomer concentration of **BNP**
[g] [**BNP**]$_0$ = 0.05 mol L^{-1} refers to the initial monomer concentration of **BNP**
[h] [**BNP**]$_0$ = 2×10^{-3} mol L^{-1} refers to the initial monomer concentration of **BNP**
[i] [**BNP**]$_0$ = 5×10^{-4} mol L^{-1} refers to the initial monomer concentration of **BNP**

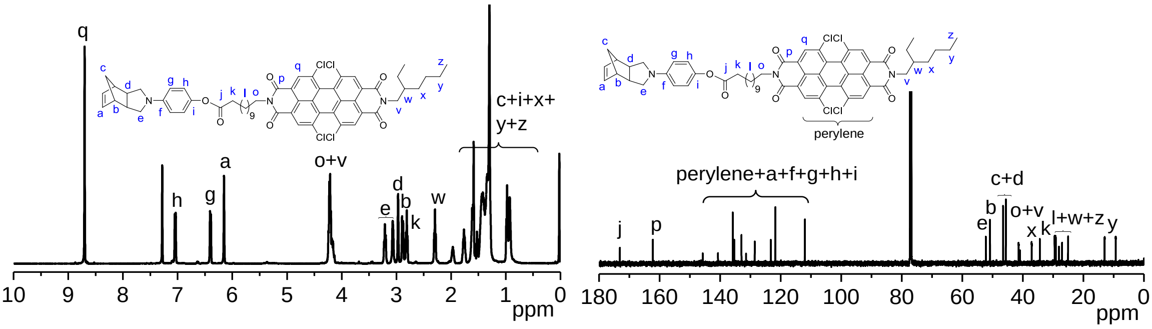

Supplementary Figure 17. ^{1}H and ^{13}C NMR spectra of **NP** in $CDCl_3$.

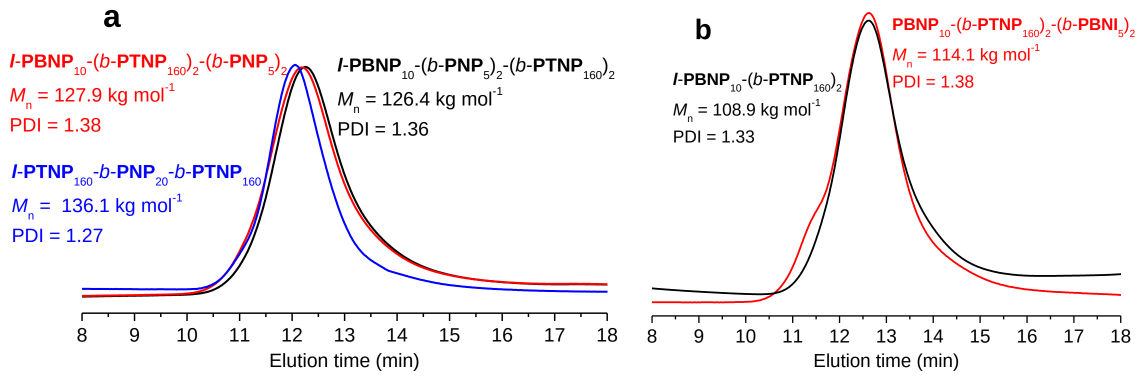

Supplementary Figure 18. GPC traces of copolymers with or without the ladderphane segment (a) and the flexible linked segment (b).

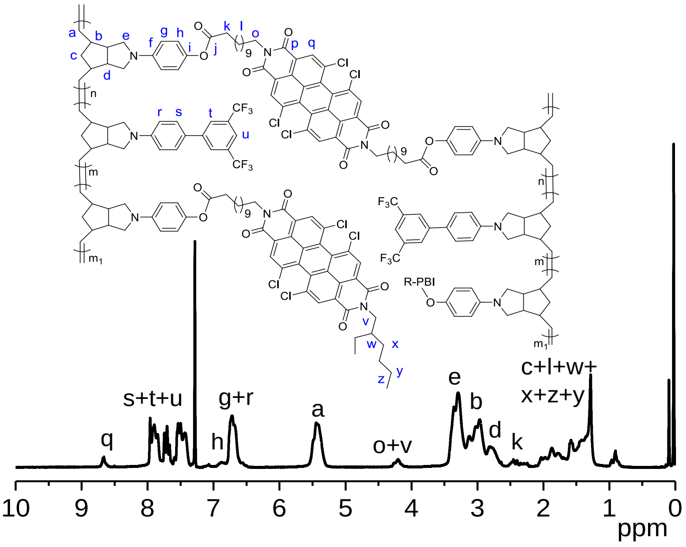

Supplementary Figure 19. ^{1}H NMR spectrum of *l*-**PBNP**$_{10}$-(*b*-**PTNP**$_{160}$)$_2$-(*b*-**PNP**$_5$)$_2$ in $CDCl_3$.

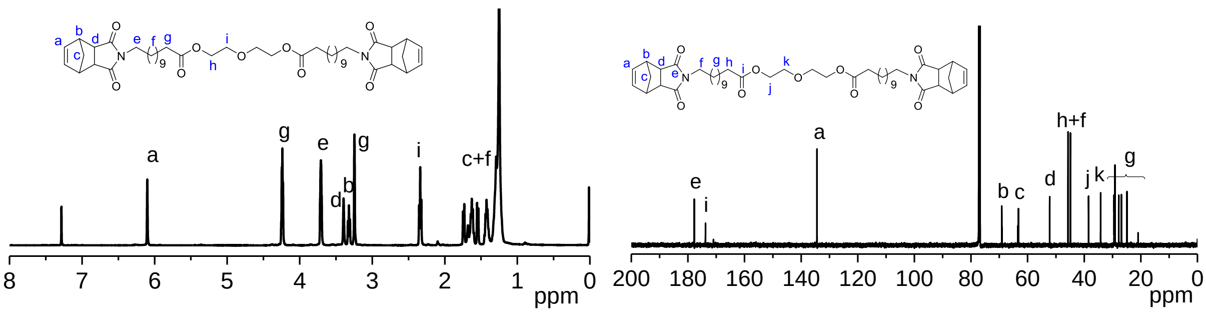

Supplementary Figure 20. ^{1}H and ^{13}C NMR spectra of **BNI** in $CDCl_3$.

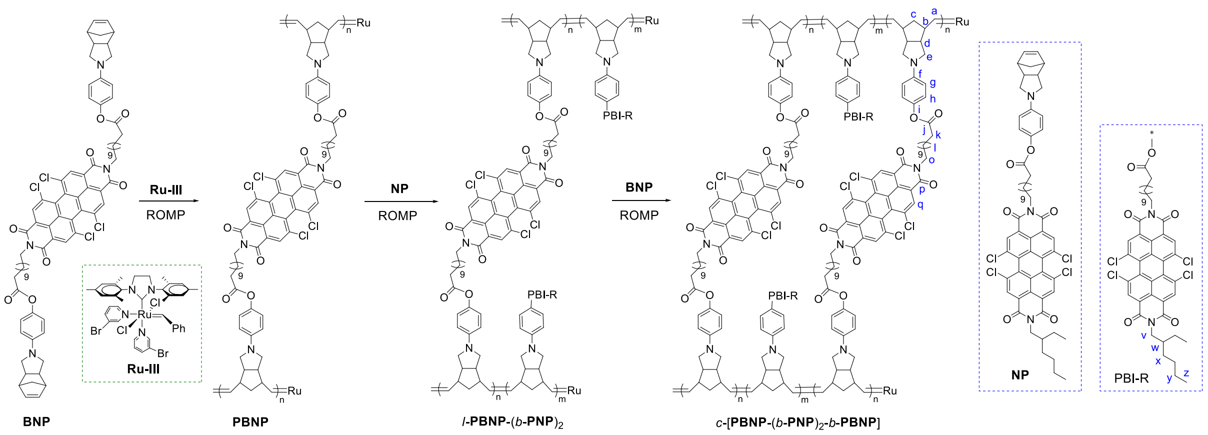

Supplementary Figure 21. Blocking-cyclization process. Syntheses of ***l*-PBNP-(*b*-PNP)**$_2$ and *c*-[**PBNP-(*b*-PNP)**$_2$-*b*-**PBNP**] by successive ROMP.

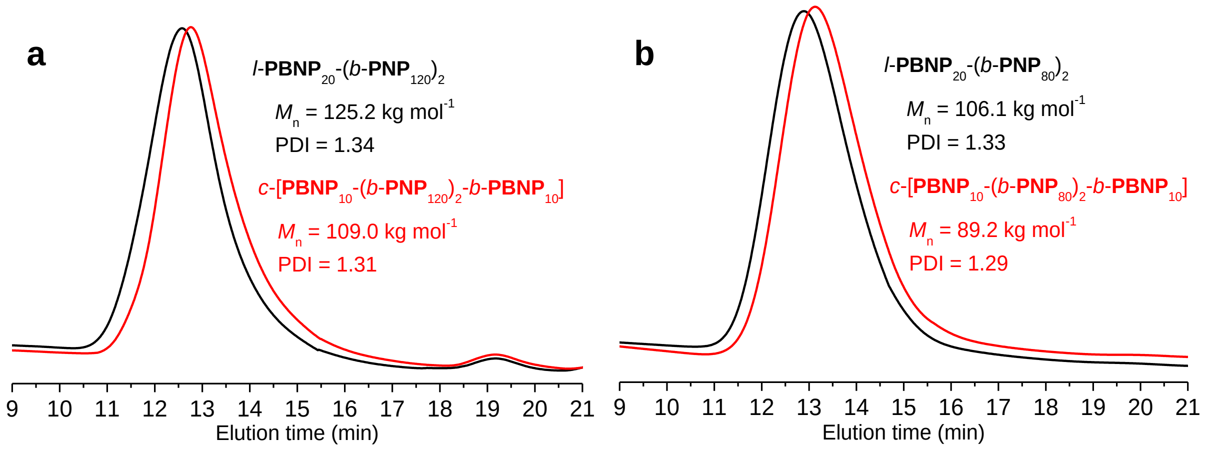

Supplementary Figure 22. GPC traces of linear and monocyclic block copolymers with rigid PBI pendants.

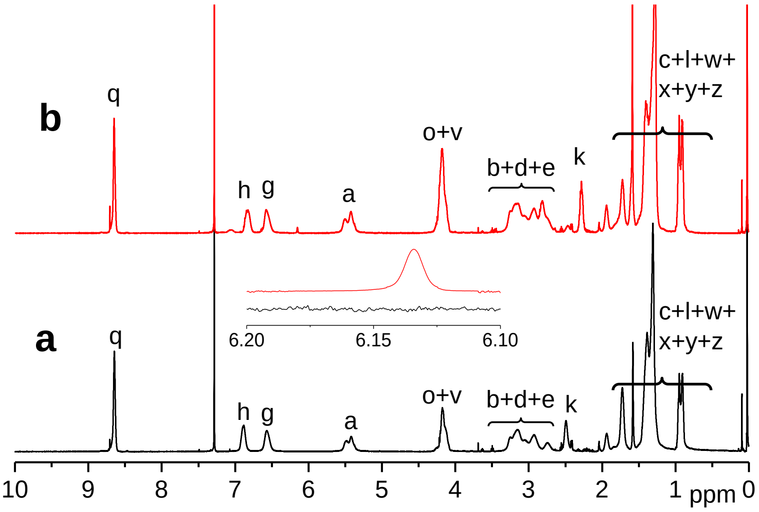

Supplementary Figure 23. ^{1}H NMR spectra of *c*-[**PBNP**$_{10}$-(*b*-**PNP**$_{120}$)$_2$-*b*-**PBNP**$_{10}$] (**a**) and *c*-[**PBNP**$_{10}$-(*b*-**PNP**$_{80}$)$_2$-*b*-**PBNP**$_{10}$] (**b**) in $CDCl_3$.

Supplementary Note 3. Verification of cyclic topology by hydrolysis of cyclic polymer

Because two **PTNP** segments in *c*-$\textbf{PBNP}_{10}$-(*b*-$\textbf{PTNP}_{160}$)$_2$-*b*-$\textbf{PBNP}_{10}$ were connected by two ladderphane **PBNP** segments with several di(lauric acid ester)-PBI linkers, therefore, each cyclic copolymer could be hydrolyzed into two linear triblock copolymer $\textbf{PTNP}_{160}$-(*b*-$\textbf{PHNP}_{10}$)$_2$ accompanying with the removal of PBI linkers under the high temperature and alkaline conditions (Supplementary Fig. 24), and the M_n of this triblock copolymer should be slightly higher than half a M_n value of $\textbf{PTNP}_{320}$. If this case was not true, the branched or partially cross-linked structure might be included in cyclic copolymer. Consequently, the hydrolytic reaction of cyclic copolymer was conducted to indirectly elucidate the validity of cyclic structure. After hydrolysis of *c*-[$\textbf{PBNP}_{10}$-(*b*-$\textbf{PTNP}_{160}$)$_2$-*b*-$\textbf{PBNP}_{10}$], the PBI signal at 8.72-8.54 ppm was disappeared in the ^{1}H NMR spectrum (Supplementary Fig. 25) of the resulting hydrolyzed polymer, suggesting that the hydrolytic reaction was performed well and the PBI linkers were eliminated completely from the original cyclic copolymer. Besides, the block ratio of **PHNP** to **PTNP** could be calculated from the ^{1}H NMR spectrum by integrating the peak area of phenyl protons (S_{f+g}) on **PHNP** blocks at 7.06-6.57 ppm and that of biphenyl protons (S_{i+j+k}) on **PTNP** block at 7.99-7.34 ppm according to the formula [**PHNP**]/[**PTNP**] = [(S_{f+g+h} - S_{i+j+k}×0.4)/4]/(S_{i+j+k}/5), giving the result of [**PHNP**]/[**PTNP**] = [(2.0-4.04×0.4)/4]/(4.04/5) = 20:168.3, which is close to the theoretical value of 20:160 in $\textbf{PTNP}_{160}$-(*b*-$\textbf{PHNP}_{10}$)$_2$, and also coincides with the ratio of 20:328.6 (entry 13, Table 1) for **PBNP** and **PTNP** segments when considering two **PTNP** segments incorporated in *c*-[$\textbf{PBNP}_{10}$-(*b*-$\textbf{PTNP}_{160}$)$_2$-*b*-$\textbf{PBNP}_{10}$]. In addition, the hydrolyzed copolymer $\textbf{PTNP}_{160}$-(*b*-$\textbf{PHNP}_{10}$)$_2$ displayed one symmetric unimodal GPC curve (Supplementary Fig. 11) with a lowered PDI of 1.33 and a reduced M_n of 57.9 kg mol^{-1}, which was expected as slightly higher than half a M_n value of $\textbf{PTNP}_{320}$, indicating that the cyclic structure of *c*-$\textbf{PBNP}_{10}$-(*b*-$\textbf{PTNP}_{160}$)$_2$-*b*-$\textbf{PBNP}_{10}$ was pure and well-defined. Otherwise, the M_n value of hydrolyzed polymer will be much large if the branched or cross-linked structure existed in cyclic polymer.

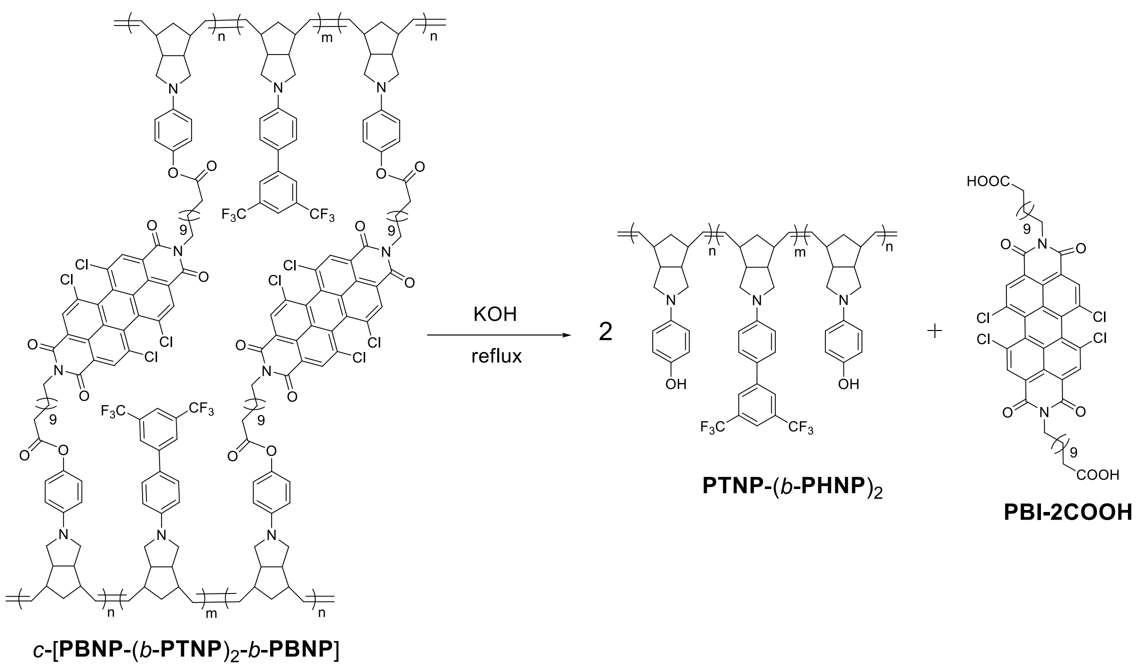

Supplementary Figure 24. Hydrolysis of monocyclic copolymer *c*-[**PBNP**$_{10}$-(*b*-**PTNP**$_{160}$)$_2$-*b*-**PBNP**$_{10}$].

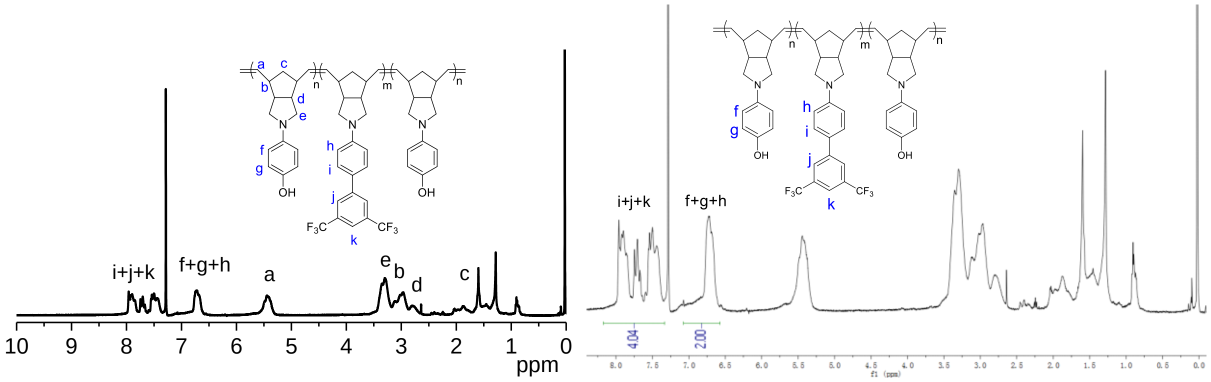

Supplementary Figure 25. ^{1}H NMR spectra of hydrolyzed copolymer **PTNP**$_{160}$-(*b*-**PHNP**$_{10}$)$_2$ in $CDCl_3$.

Supplementary Note 4. Characteristics of bis- and tricyclic polymers

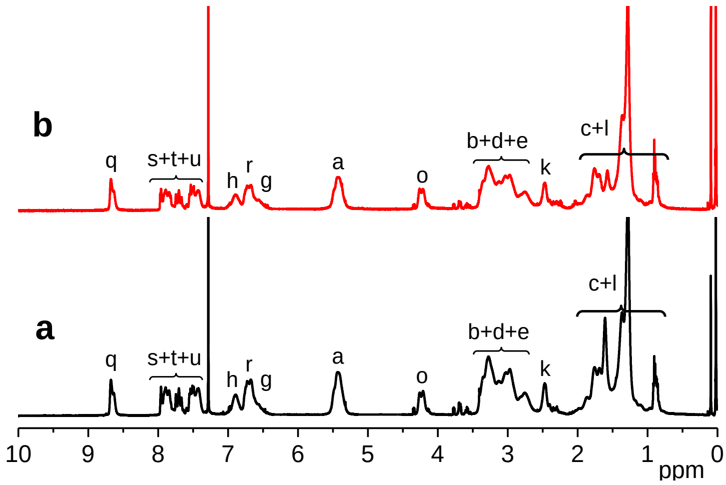

Supplementary Figure 26. ^{1}H NMR spectra of linear *l*-**PBNP**$_{30}$-(*b*-**PTNP**$_{40}$)$_2$ (a) and biscyclic *c*-[**PBNP**$_{10}$-((*b*-**PTNP**$_{20}$)$_2$-*b*-**PBNP**$_{10}$)$_2$] (b) in $CDCl_3$.

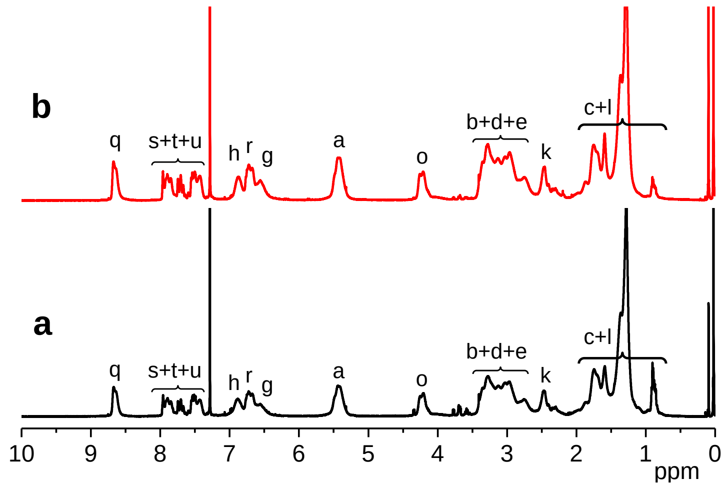

Supplementary Figure 27. ^{1}H NMR spectra of linear *l*-**PBNP**$_{40}$-(*b*-**PTNP**$_{60}$)$_2$ (a) and tricyclic *c*-[**PBNP**$_{10}$-((*b*-**PTNP**$_{20}$)$_2$-*b*-**PBNP**$_{10}$)$_3$] (b) in $CDCl_3$.

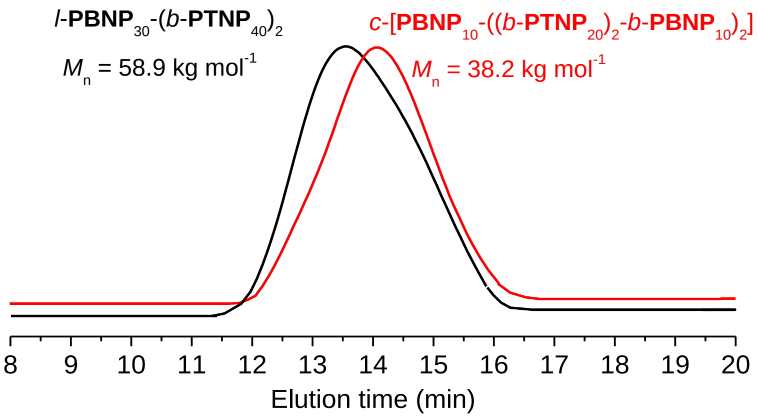

Supplementary Figure 28. GPC traces of linear and biscyclic block copolymers.

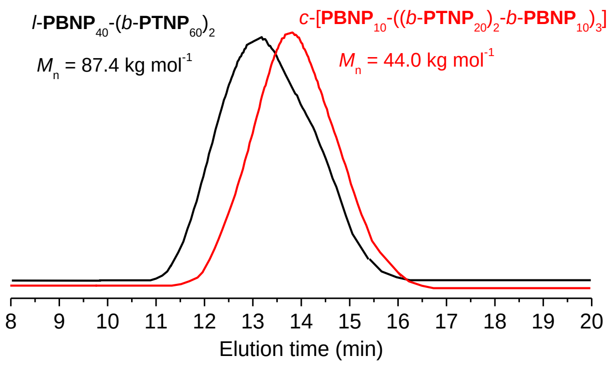

Supplementary Figure 29. GPC traces of linear and tricyclic block copolymers.

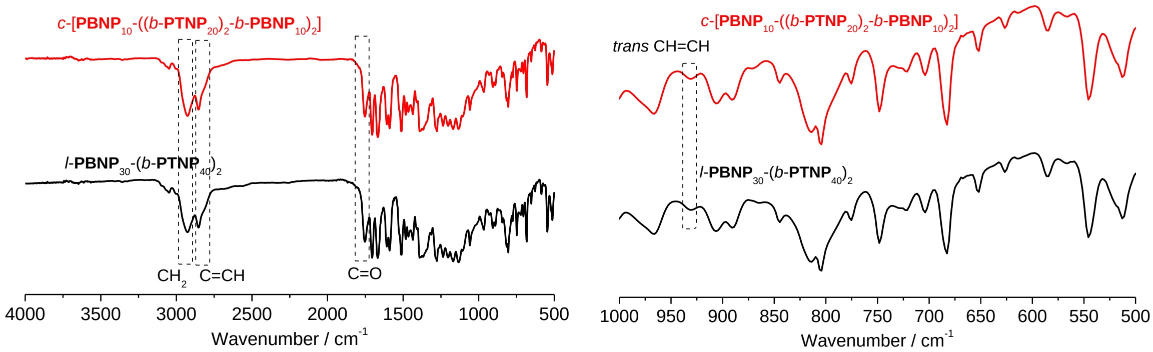

Supplementary Figure 30. IR spectra of *l*-**PBNP**30-(*b*-**PTNP**40)2 and biscyclic *c*-[**PBNP**10-((*b*-**PTNP**20)2-*b*-**PBNP**10)2].

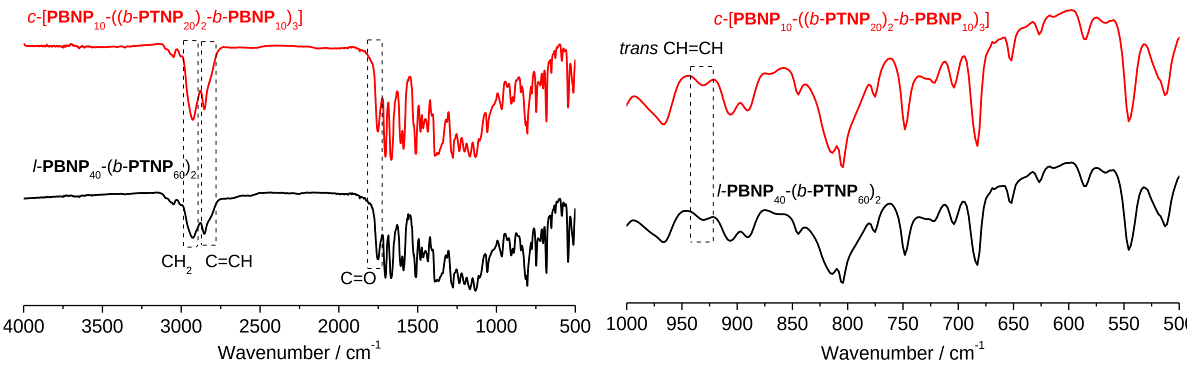

Supplementary Figure 31. IR spectra of *l*-**PBNP**40-(*b*-**PTNP**60)2 and tricyclic *c*-[**PBNP**10-((*b*-**PTNP**20)2-*b*-**PBNP**10)3].

Supplementary Note 5. Thermal properties of linear and cyclic polymers

The thermal stability of polymers was investigated by the thermal gravimetric analysis (TGA). The thermal decomposition temperatures (T_d, 5% weight loss) of linear and cyclic polymers were all over 380 ^oC, and about 30% of residue was retained at 600 ^oC (Supplementary Fig. 32 and Supplementary Table 2). All polymers exhibited only one stage decomposition, because of almost the same decomposition behavior for **PTNP** and **PBNP** blocks. The results fully confirmed the better thermal resistance, which was attributed to the rigid ladderphane structure and the cyclic topology.

According to chain-end free volume theory,[15-17] a cyclic polymer tends to have a higher glass transition temperature (T_g) than its linear analogue, because cyclic polymer without chain-ends exhibits a more rigid conformation. The T_gs of linear and cyclic polymers were tested by differential scanning calorimeter (DSC) technique, and the effect of cyclic topology on the thermal behavior was remarkable (Supplementary Table 2). For the monocyclic polymers *c*-[**PBNP**-(*b*-**PTNP**)$_2$-*b*-**PBNP**], the DSC plots showed only one T_g (Supplementary Fig. 33) for each cyclic polymer, which increased slightly as the repeat units or M_n of polymers and is much higher than the linear analogue (197 *vs* 173, 201 *vs* 175, 203 *vs* 178, 203 *vs* 179, and 203 *vs* 182 ^oC), implying that the linear intermediate **PBNP**-(*b*-**PTNP**)$_2$ was successfully cyclized rather than generated a mixture of linear and cyclic structures. Other than a kind of traditional cyclic polymers with flexible chain without rigid aromatic structure, the T_g of each cyclic polymer was 20 ^oC more higher than that of its linear analogue, which might be attributed to the rigid structure of ladderphane **PBNP** and the π-π stacking effect of **PTNP** segments. In block copolymers, the linear **PTNP** segment by ROMP was basically to take stereoregularity configuration,[6] which made it easy to get close to the adjacent pendant groups by the π-π interaction. Although the average space between the adjacent pendant groups was 0.65 nm,[18] the part of flexible **PTNP** backbone was curved, so that the average space decreased to 0.37 nm to match the distance requirement of π-π stacking (Supplementary Fig. 34). Contrast to linear copolymers, two **PTNP** segments in single cyclic macromolecule were not far from each other because of the cyclic topology. Beyond the π-π stacking effect of adjacent pendant groups in one bent **PTNP** segment, some of biphenyl groups in two **PTNP** segments got into each other's space between the adjacent pendants (Supplementary Fig. 35), because the electron cloud density difference between two phenyl groups in biphenyl moiety made electron-deficient phenyl easier to stack on electron-rich phenyl, which formed the intra-chain entanglement junctions by tangling under the condition of π-π stacking effect,[19] and enhanced the rigidity of polymer chain, resulting in an increased T_g.

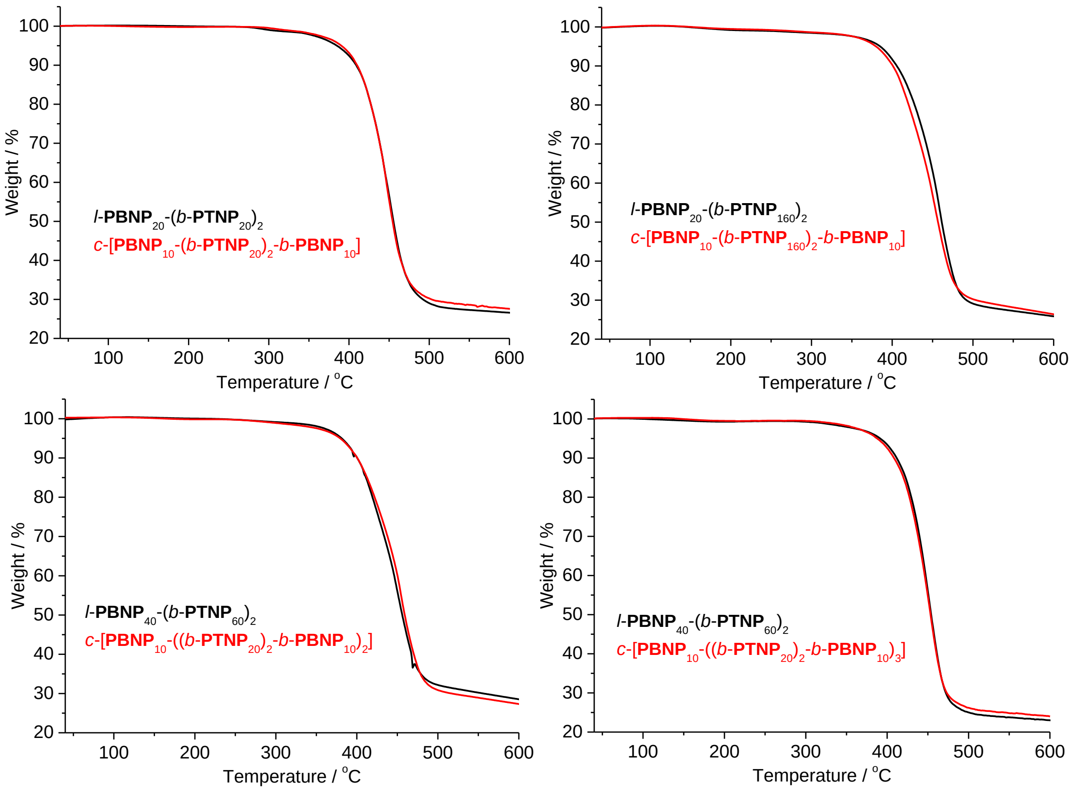

Supplementary Figure 32. TGA curves of linear and mono-, bis- and tricyclic block copolymers.

Supplementary Table 2 Thermal properties and sizes of linear and cyclic polymers

**Entry**	**Polymer**	T_g[a] (ºC)	T_d[b] (ºC)	D_h[c] (nm)
1	l-**PBNP**$_{20}$-(b-**PTNP**$_{20}$)$_2$	173	381	4.2
2	c-[**PBNP**$_{10}$-(b-**PTNP**$_{20}$)$_2$-b-**PBNP**$_{10}$]	197	386	3.5
3	l-**PBNP**$_{20}$-(b-**PTNP**$_{40}$)$_2$	175	/	/
4	c-[**PBNP**$_{10}$-(b-**PTNP**$_{40}$)$_2$-b-**PBNP**$_{10}$]	201	/	/
5	l-**PBNP**$_{20}$-(b-**PTNP**$_{80}$)$_2$	178	/	4.7
6	c-[**PBNP**$_{10}$-(b-**PTNP**$_{80}$)$_2$-b-**PBNP**$_{10}$]	203	/	4.0
7	l-**PBNP**$_{20}$-(b-**PTNP**$_{120}$)$_2$	179	/	/
8	c-[**PBNP**$_{10}$-(b-**PTNP**$_{120}$)$_2$-b-**PBNP**$_{10}$]	203	/	/
9	l-**PBNP**$_{20}$-(b-**PTNP**$_{160}$)$_2$	180	382	4.8
10	c-[**PBNP**$_{10}$-(b-**PTNP**$_{160}$)$_2$-b-**PBNP**$_{10}$]	203	387	4.3
11	l-**PBNP**$_{20}$-(b-**PTNP**$_{200}$)$_2$	182	/	/
12	c-[**PBNP**$_{10}$-(b-**PTNP**$_{200}$)$_2$-b-**PBNP**$_{10}$]	204	/	/
13	l-**PBNP**$_{30}$-(b-**PTNP**$_{40}$)$_2$	180	382	5.4
14	c-[**PBNP**$_{10}$-((b-**PTNP**$_{20}$)$_2$-b-**PBNP**$_{10}$)$_2$]	201	382	3.4
15	l-**PBNP**$_{40}$-(b-**PTNP**$_{60}$)$_2$	182	385	6.1
16	c-[**PBNP**$_{10}$-((b-**PTNP**$_{20}$)$_2$-b-**PBNP**$_{10}$)$_3$]	203	384	3.4
17	l-**PBNP**$_{20}$-(b-**PTNP**$_{80}$)$_2$-**POSS**	/	/	7.0/22
18	c-[**PBNP**$_{10}$-(b-**PTNP**$_{80}$)$_2$-b-**PBNP**$_{10}$]-**POSS**	/	/	6.9/19
19	l-**PBNP**$_{20}$-(b-**PTNP**$_{160}$)$_2$-**POSS**	/	/	7.2/23
20	c-[**PBNP**$_{10}$-(b-**PTNP**$_{160}$)$_2$-b-**PBNP**$_{10}$]-**POSS**	/	/	7.0/22

[a] Determined by DSC. [b] Determined by TGA. [c] Determined by DLS.

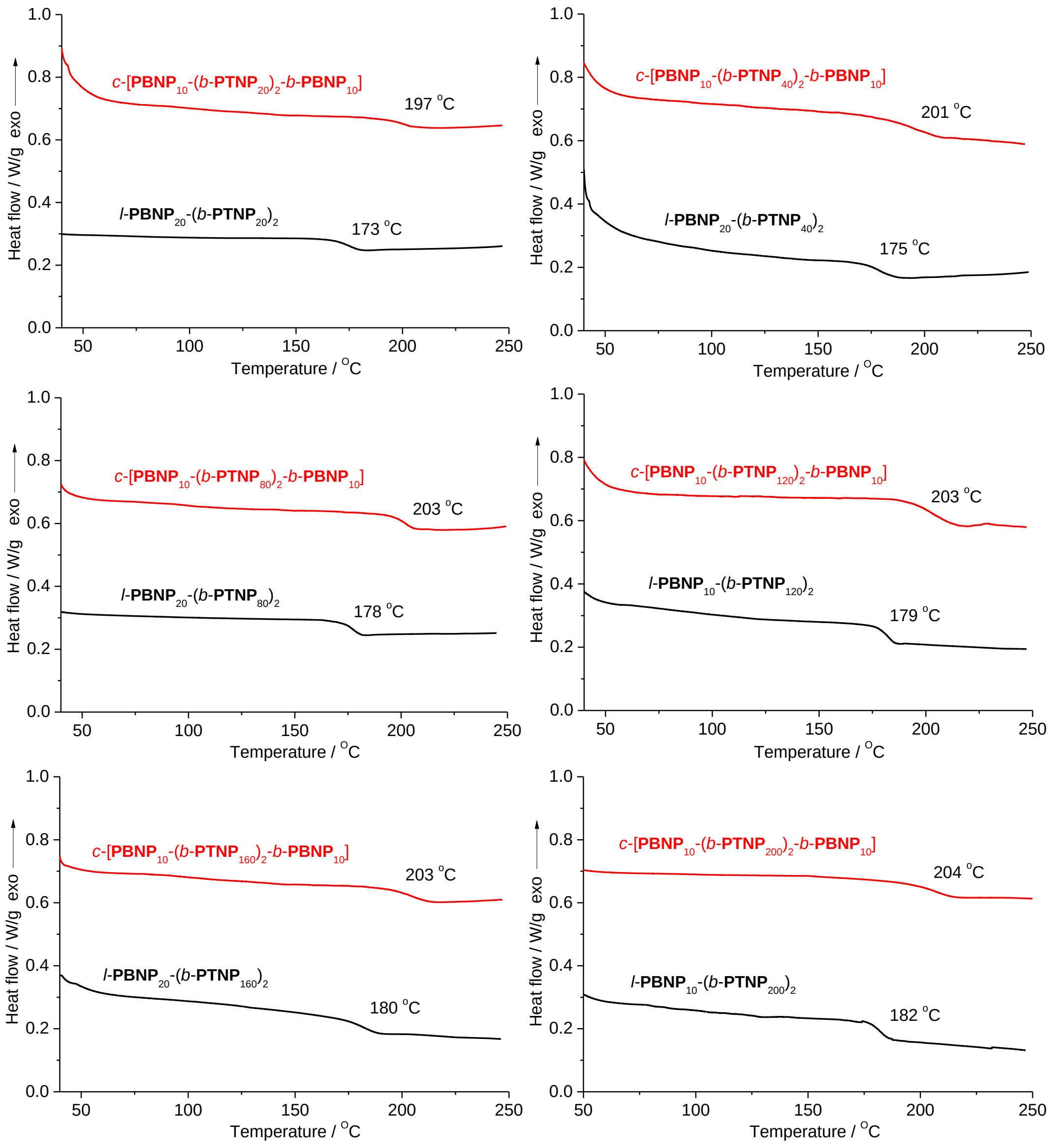

Supplementary Figure 33. DSC curves of linear and monocyclic block copolymers in the second heating process.

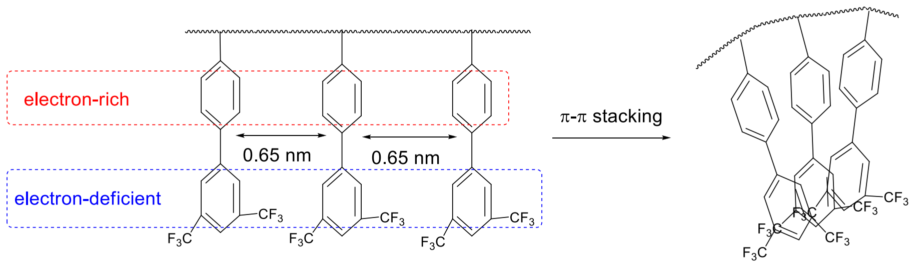

Supplementary Figure 34. The twisted part of flexible PNBE backbone by π-π stacking effect.

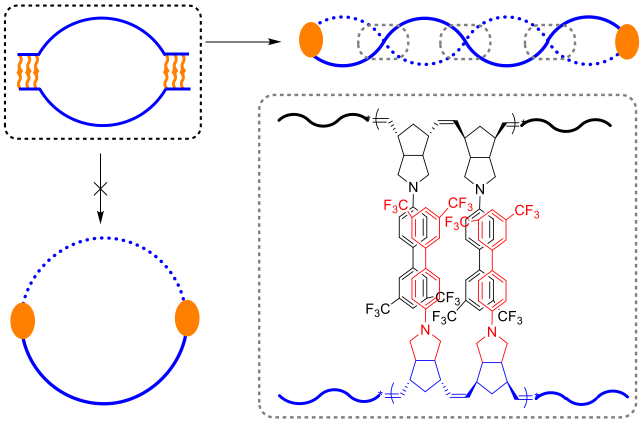

Supplementary Figure 35. Schematic illustration for the intra-chain entanglement junctions of cyclic polymers.

Similarly, the T_gs of biscyclic *c*-[**PBNP**$_{10}$-((*b*-**PTNP**$_{20}$)$_2$-*b*-**PBNP**$_{10}$)$_2$] and tricyclic *c*-[**PBNP**$_{10}$-((*b*-**PTNP**$_{20}$)$_2$-*b*-**PBNP**$_{10}$)$_3$] reached to 201 °C and 203 °C, respectively, the T_gs of their linear counterparts were 180 °C and 182 °C, respectively, and the T_g value of each cyclic polymer was also 20 °C more higher than that of its linear analogue (Supplementary Fig. 36). Therefore, the feature of drastic up in T_g is indicative of such cyclic polymer containing the rigid ladderphane blocks.

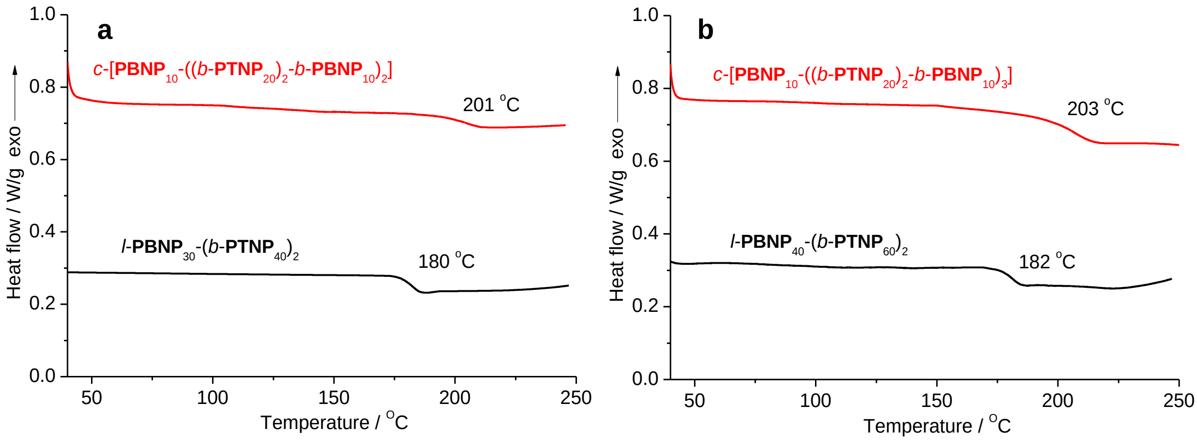

Supplementary Figure 36. DSC curves of linear *l*-**PBNP**$_{30}$-(*b*-**PTNP**$_{40}$)$_2$ and biscyclic *c*-[**PBNP**$_{10}$-((*b*-**PTNP**$_{20}$)$_2$-*b*-**PBNP**$_{10}$)$_2$] (a) as well as linear *l*-**PBNP**$_{40}$-(*b*-**PTNP**$_{60}$)$_2$ and tricyclic *c*-[**PBNP**$_{10}$-((*b*-**PTNP**$_{20}$)$_2$-*b*-**PBNP**$_{10}$)$_3$] (b) in the second heating process.

Supplementary Note 6. Post-polymerization of monocyclic polymers

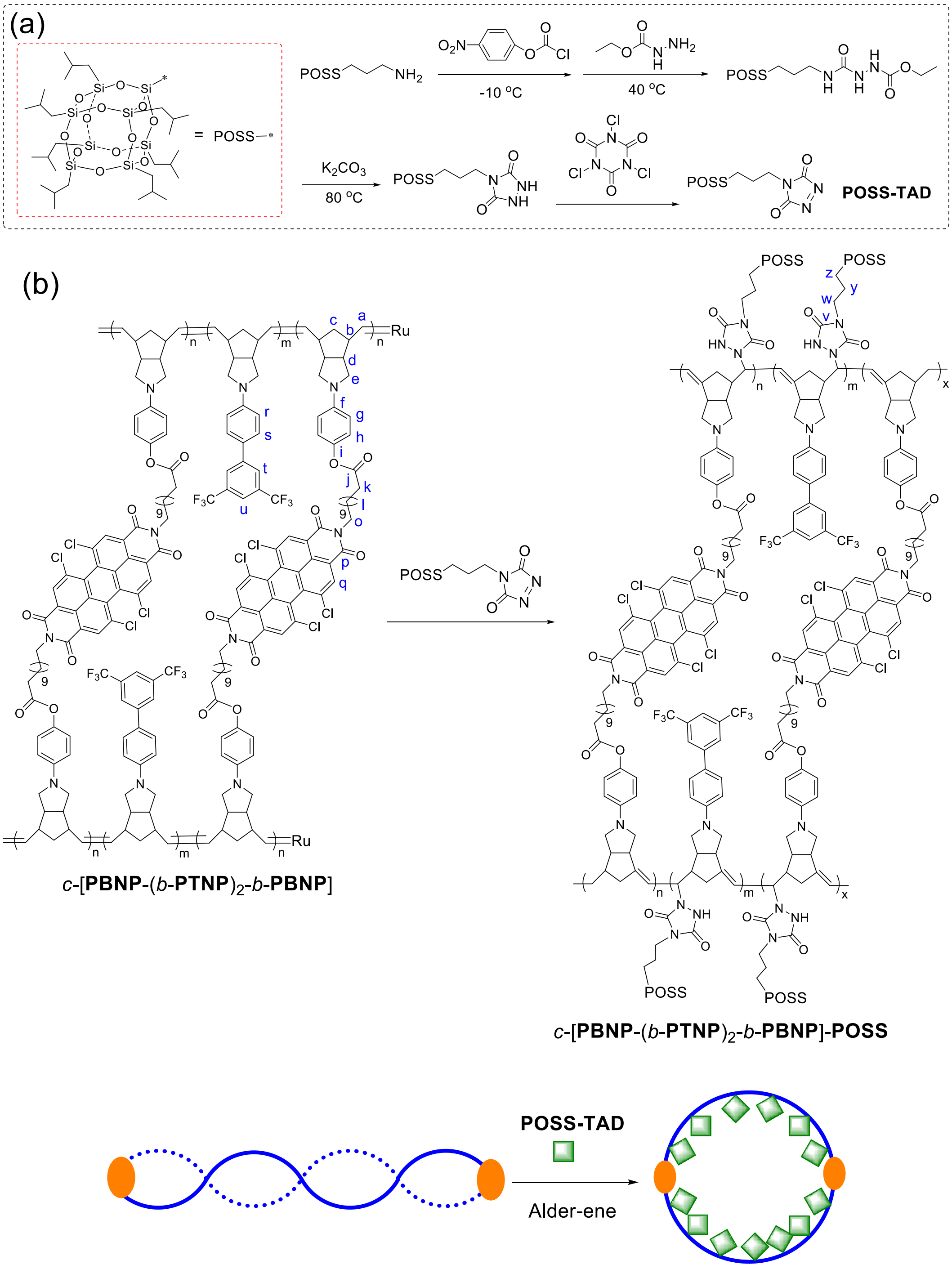

Supplementary Figure 37. Preparation of **POSS-TAD** (a) and the modified cyclic polymer (b).

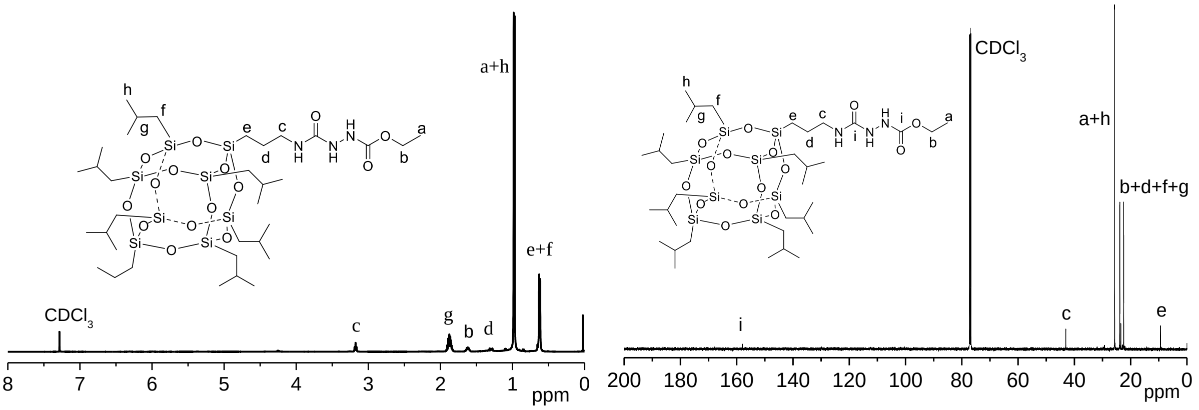

Supplementary Figure 38. ^{1}H and ^{13}C NMR spectra of POSS propylisobutyl-1-carbethoxysemicarbazide.

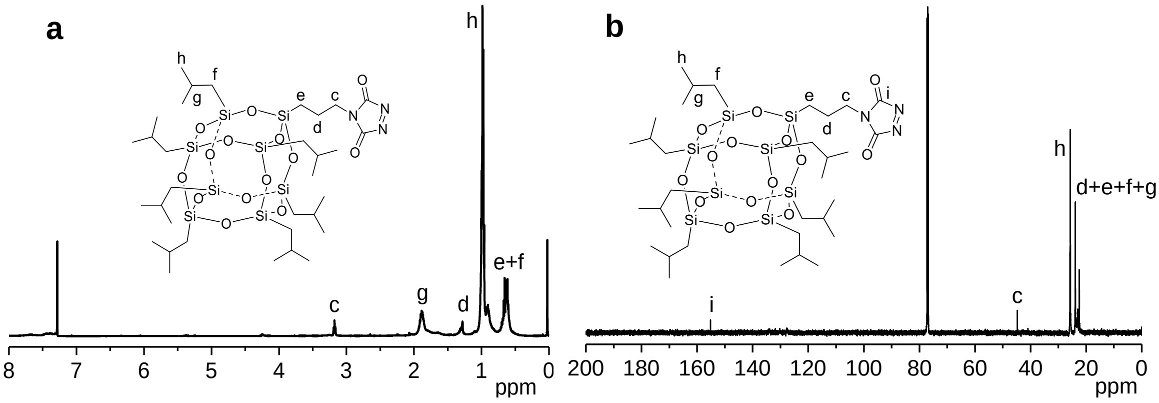

Supplementary Figure 39. ^{1}H (a) and ^{13}C NMR (b) spectra of **POSS-TAD** in $CDCl_3$.

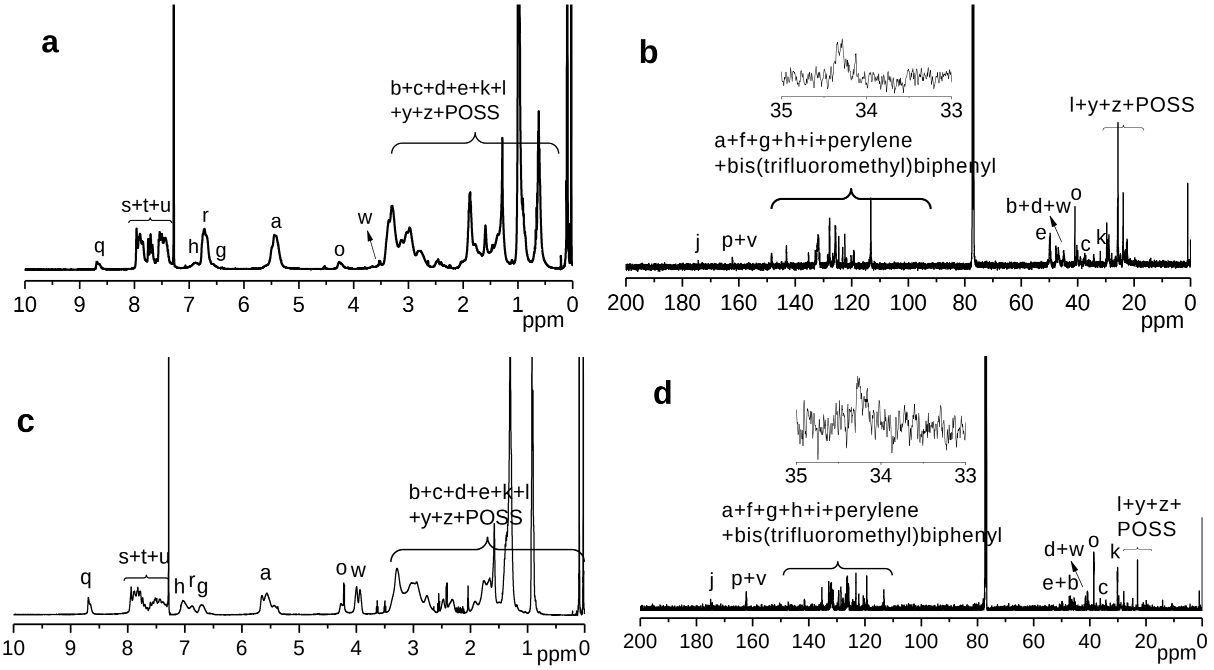

Supplementary Figure 40. ^{1}H (a,c) and ^{13}C (b,d) NMR spectra of *c*-[**PBNP**$_{10}$-(*b*-**PTNP**$_{160}$)$_2$-*b*-**PBNP**$_{10}$]-**POSS** (a,b) and *l*-**PBNP**$_{20}$-(*b*-**PTNP**$_{160}$)$_2$-**POSS** (c,d).

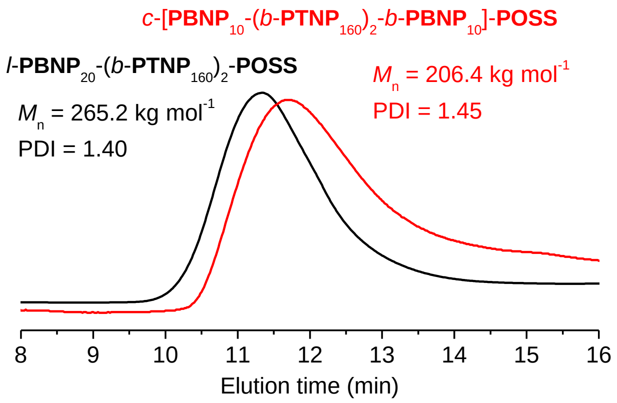

Supplementary Figure 41. GPC traces of *l*-**PBNP**$_{10}$-(*b*-**PTNP**$_{160}$)$_2$-**POSS** and *c*-[**PBNP**$_{10}$-(*b*-**PTNP**$_{160}$)$_2$-*b*-**PBNP**$_{10}$]-**POSS**.

Supplementary Note 7. Photophysical properties of linear and cyclic polymers

Although the intra-chain entanglement junctions could not be observed directly, its effect would be readily detected by monitoring the optical properties[20-22] and the chain aggregation of polymers.[19] The UV-vis absorption spectra of linear *l*-**PBNP**$_{20}$-(*b*-**PTNP**$_{20}$)$_2$, *l*-**PBNP**$_{20}$-(*b*-**PTNP**$_{40}$)$_2$, and *l*-**PBNP**$_{20}$-(*b*-**PTNP**$_{80}$)$_2$, as well as the corresponding monocyclic *c*-[**PBNP**$_{10}$-(*b*-**PTNP**$_{20}$)$_2$-*b*-**PBNP**$_{10}$], *c*-[**PBNP**$_{10}$-(*b*-**PTNP**$_{40}$)$_2$-*b*-**PBNP**$_{10}$], and *c*-[**PBNP**$_{10}$-(*b*-**PTNP**$_{80}$)$_2$-*b*-**PBNP**$_{10}$] showed the characteristic absorptions at 400-600 nm for PBI linker in ladderphane **PBNP** segment and at 265-385 nm for **PTNP** segment (Supplementary Fig. 42a-c). The absorption at 265-385 nm strengthened with the [**TNP**]/[**BNP**] ratio increase, which proved that **PTNP** block really lengthened as increasing **TNP** loading and was connected to the ladderphane **PBNP** segment. In particular, for the same **PTNP**/**PBNP** ratio, the absorption strength of **PTNP** segments in each cyclic polymer was stronger than that of **PTNP** segments in linear counterpart, and the absorption difference obviously enhanced with the **PTNP** chain length increase, which may because that the long cyclic polymer chain had more intra-chain entanglement junctions so as to promote the absorption strength of polymer due to the π-π interaction effect. Similarly, the absorption strength of bis- and tricyclic polymers at 265-385 nm was stronger than those of their linear counterparts, and the discrepancy increased with the ring number increase (Supplementary Fig. 43). Therefore, the result from the UV-vis characterization can be used as an evidence for comparing and confirming the cyclic structure, ring size, and ring number. The disruption of the stereoregularity microstructure feature of PNBE backbone after connection of POSS moiety resulted in decrease of the π-π interaction effect, while the adjacent pendant groups in the **PTNP** segment got away from each other. Therefore, the entangled **PTNP** segments were pushed apart and the intra-chain entanglement junctions disappeared in POSS-modified cyclic polymers. Although the absorption of **PTNP** blocks at 265-385 nm strengthened with the increase of **PTNP**/**PBNP** ratio after POSS modification (Supplementary Fig. 42d-f), more interestingly, the characteristic absorptions of the **PTNP** segments in POSS-modified cyclic polymers were similar to those of the linear analogues by the good superposition of curves (Supplementary Fig. 44), because there was no any π-π stacking effect of intra-chain entanglement junctions in the modified cyclic polymers, which confirmed the speculation of intra-chain entanglement junctions in cyclic polymers.

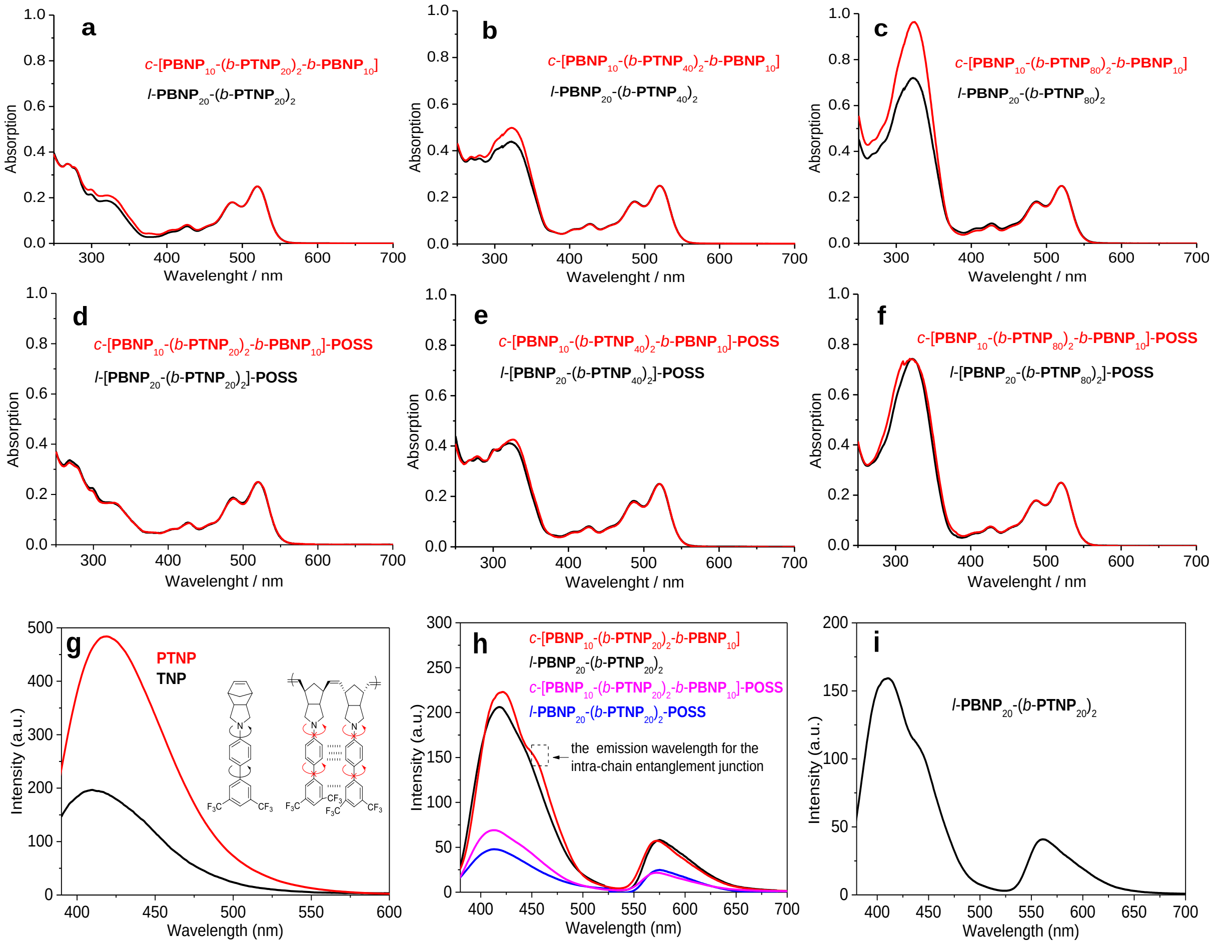

Supplementary Figure 42. Photophysical features. UV-vis absorption spectra of linear and monocyclic polymers before (**a-c**) and after (**d-f**) POSS modification at 0.01 mg mL^{-1} in $CHCl_3$. Fluorescence emission spectra of monomer and homopolymer (**g**), copolymers with or without POSS modification (**h**) at 0.01 mg mL^{-1}, and *l*-**PBNP**$_{20}$-(*b*-**PTNP**$_{20}$)$_2$ (**i**) at 10 mg mL^{-1} in $CHCl_3$ under different maximum excited wavelengths for each monomer and polymer. The inset in (**g**) is the diagrammatic illustration for the rotation of biphenyl pendant groups.

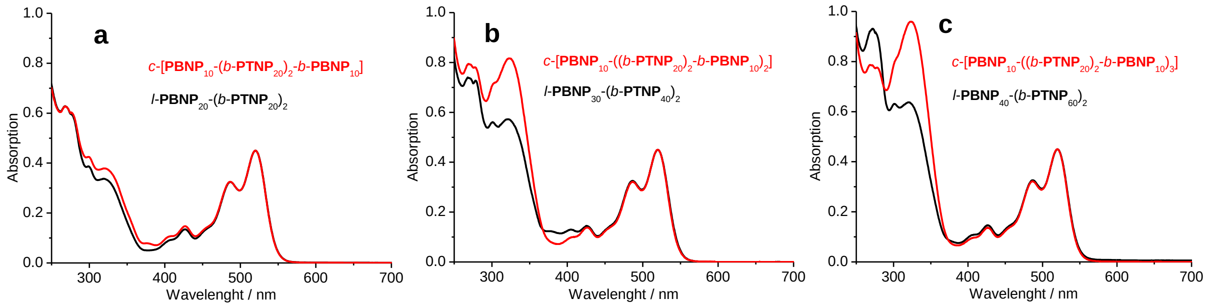

Supplementary Figure 43. UV-vis absorption spectra of linear and the corresponding mono- (a), bis- (b), and tricyclic (c) block copolymers at 0.01 mg mL^{-1} in $CHCl_3$.

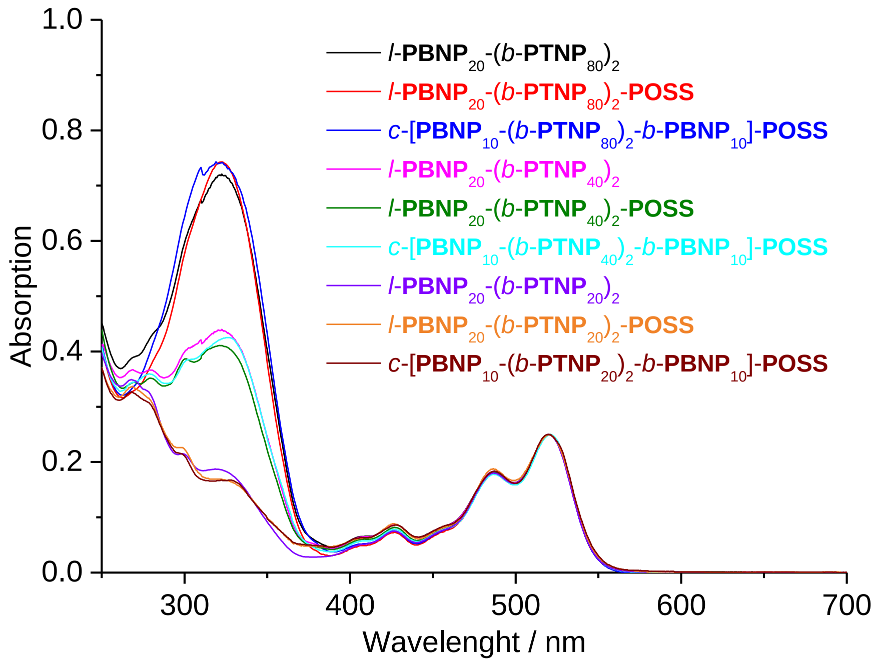

Supplementary Figure 44. UV-vis absorption spectra of linear, cyclic, and the corresponding **POSS-TAD** modified block copolymers at 0.01 mg mL^{-1} in $CHCl_3$.

The maximum excitation wavelength of **TNP**, **PTNP**, *l*-**PBNP**$_{20}$-(*b*-**PTNP**$_{20}$)$_2$, *c*-[**PBNP**$_{10}$-(*b*-**PTNP**$_{20}$)$_2$-*b*-**PBNP**$_{10}$], *l*-**PBNP**$_{20}$-(*b*-**PTNP**$_{20}$)$_2$-**POSS,** and *c*-[**PBNP**$_{10}$-(*b*-**PTNP**$_{20}$)$_2$-*b*-**PBNP**$_{10}$]-**POSS** were 352, 364, 366, 372, 342, and 362 nm in sequence (Supplementary Fig. 45). With the increase of the π-π stacking effect, the corresponding maximum excitation wavelength red-shifted. The emission wavelengths of **PBNP** and **PTNP** blocks were appeared at 525-700 and 380-520 nm, respectively, in the fluorescence emission spectra under the maximum excitation wavelength (Supplementary Fig. 42g-i), and the inter-chain interaction of polymers could be almost ignored in a low concentration solution. Firstly, by comparing the emission intensity of monomer **TNP** and homopolymer **PTNP**, **TNP** has a lower emission intensity than that of **PTNP** under the same concentration of 0.01 mg mL^{-1}, and the maximum emission wavelength red-shifted from 410 to 419 nm (Supplementary Fig. 42g). This was because that the biphenyl groups in **TNP** could be easy to rotate freely when the molecules absorbed light energy in the solution, resulting in that most of the energy was consumed and the emission intensity weakened.[23] For **PTNP**, however, the π-π stacking effect of adjacent biphenyl pendant groups inhibited the rotation due to the *trans*-configuration and high stereoregularity of PNBE backbone, so the emission intensity was stronger. Then, for the linear and cyclic copolymers, taking *l*-**PBNP**$_{20}$-(*b*-**PTNP**$_{20}$)$_2$ and *c*-[**PBNP**$_{10}$-(*b*-**PTNP**$_{20}$)$_2$-*b*-**PBNP**$_{10}$] as the typical examples, their emission intensities at 525-700 nm were almost equal under the maximum excited wavelength

(Supplementary Fig. 42h and Supplementary Table 3), due to the same concentration and molecular chain structure of **PBNP** segments. Differently, the maximum emission intensity and wavelength at 380-520 nm of *c*-[**PBNP**$_{10}$-(*b*-**PTNP**$_{20}$)$_2$-*b*-**PBNP**$_{10}$] was greater than the linear analogue, because the two **PTNP** segments in a single cyclic polymer molecule was easier to be entangled itself in solution and became more rigid than that of linear analogue, which contributed to reduce the energy loss of fluorescence emission caused by inhibiting local polymer chain motion.[24,25] In addition, the emerging emission peak at 455 nm for the intra-chain entanglement junctions of *c*-[**PBNP**$_{10}$-(*b*-**PTNP**$_{20}$)$_2$-*b*-**PBNP**$_{10}$] was observed at a low concentration of 0.01 mg mL^{-1} (Supplementary Fig. 42h). As the concentration of *l*-**PBNP**$_{20}$-(*b*-**PTNP**$_{20}$)$_2$ increased to 10 mg mL^{-1}, due to the existence of inter-chain entanglement junctions, the emission peak at 455 nm could be also observed (Supplementary Fig. 42i). When the POSS moiety was attached to the backbone of *c*-[**PBNP**$_{10}$-(*b*-**PTNP**$_{20}$)$_2$-*b*-**PBNP**$_{10}$], the emission peak at 455 nm disappeared (Supplementary Fig. 42h), because the intra-chain entanglement junctions were depressed. Besides, for the POSS-modified *l*-**PBNP**$_{20}$-(*b*-**PTNP**$_{20}$)$_2$-**POSS** and *c*-[**PBNP**$_{10}$-(*b*-**PTNP**$_{20}$)$_2$-*b*-**PBNP**$_{10}$]-**POSS**, although the emission intensity of **PBNP** block at 525-700 nm was unchanged, the emission intensity and wavelength of **PTNP** block at 380-520 nm weakened and blue-shifted (from 418 to 411 nm and 420 to 413 nm) substantially in comparison to those of linear and cyclic copolymers without POSS moiety, because the partially disappeared π-π stacking effect made the part of biphenyl group rotate freely in the excitation light, resulting in a loss of emission energy. If excited under wavelength λ = 525 nm (Supplementary Fig. 46a), because the repeat unit number of one ladderphane **PBNP** block in linear copolymers was twice of each ladderphane **PBNP** block in cyclic polymers, the more co-facial PBI linkers in the former further promoted electron delocalization, which caused a slight red-shift in fluorescence emission of linear copolymers with or without POSS modification (Supplementary Fig. 46b).

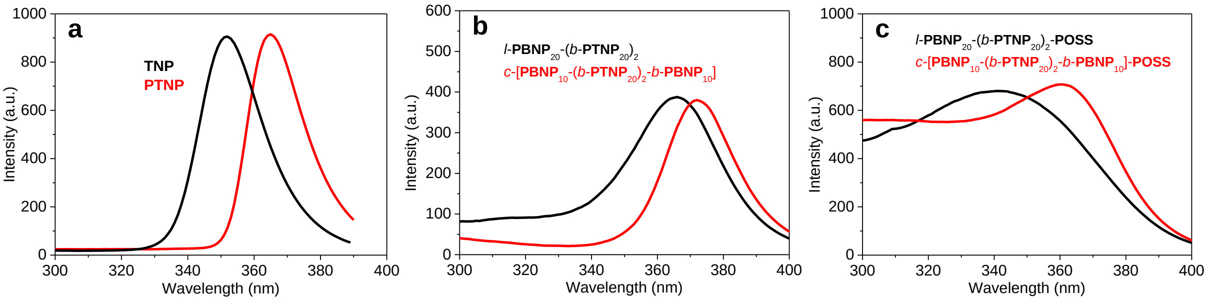

Supplementary Figure 45. Fluorescence excitation spectra of monomer **TNP** and polymer **PTNP** at 0.01 mg mL^{-1} in $CHCl_3$ under the maximum emission wavelengths.

Supplementary Table 3 Fluorescence analysis for polymers[a]

Entry	Polymer	I_1[b]	I_2[c]	K[d]
1	l-**PBNP**$_{20}$-(b-**PTNP**$_{40}$)$_2$	205.99	56.70	3.63
2	c-[**PBNP**$_{10}$-((b-**PTNP**$_{40}$)$_2$-b-**PBNP**$_{10}$)]	223.82	56.70	3.94
3	l-**PBNP**$_{20}$-(b-**PTNP**$_{40}$)$_2$-**POSS**	48.45	24.74	1.96
4	c-[**PBNP**$_{10}$-((b-**PTNP**$_{40}$)$_2$-b-**PBNP**$_{10}$)]-**POSS**	68.83	22.47	3.06

[a] Testing conditions: the samples were excited at 380 nm and emission spectra were recorded from 380 to 700 nm; the concentration of polymers was 0.01 mg mL^{-1}; and the solvent was $CHCl_3$.

[b] The maximum fluorescence emission intensity of **PTNP** block.

[c] The maximum fluorescence emission intensity of **PBNP** block.

[d] K = I_1/I_2.

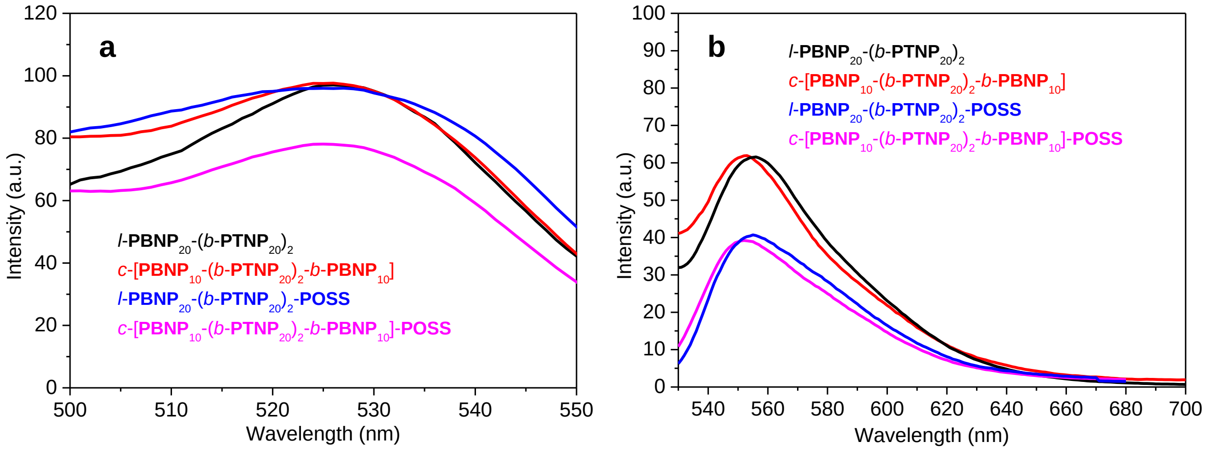

Supplementary Figure 46. Fluorescence excitation (a) and emission (b) spectra of **PBNP** segments in copolymers at 0.01 mg mL^{-1} in $CHCl_3$ under excited wavelength λ = 525 nm.

Supplementary Note 8. Hydrodynamic diameter and topology of linear and cyclic polymers

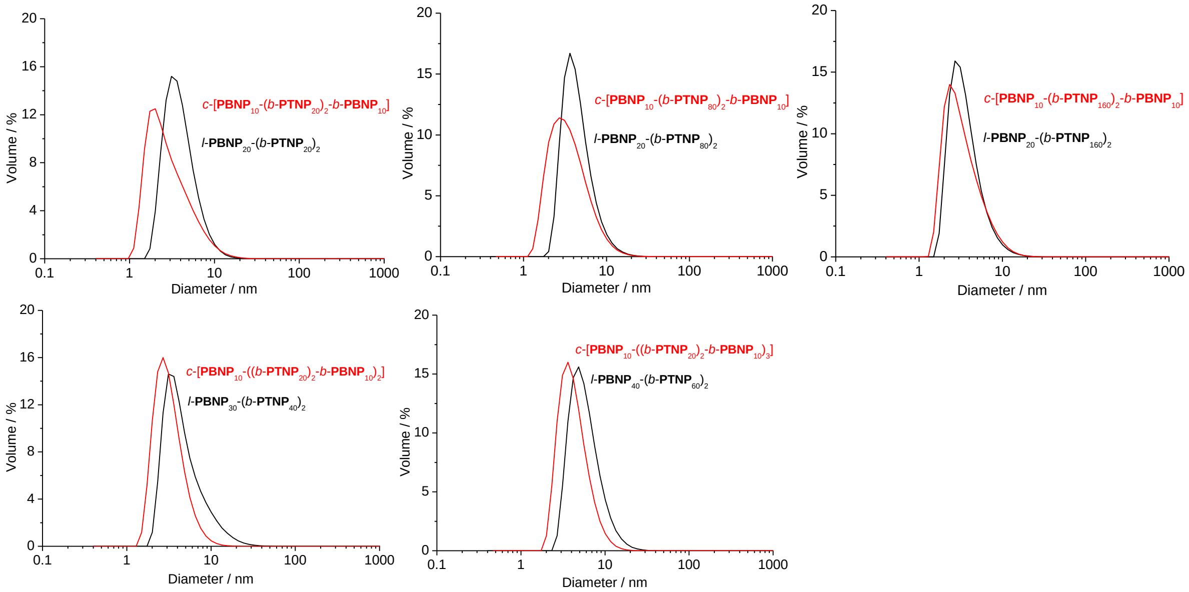

Supplementary Figure 47. Sizes of polymers in $CHCl_3$ at 0.1 mg mL^{-1} by means of DLS.

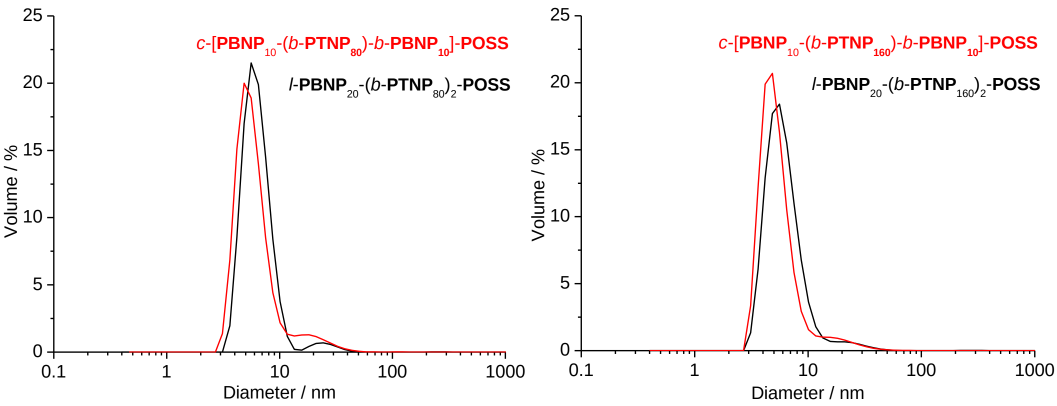

Supplementary Figure 48. Sizes of **POSS-TAD** modified polymers in $CHCl_3$ at 0.1 mg mL^{-1} by DLS.

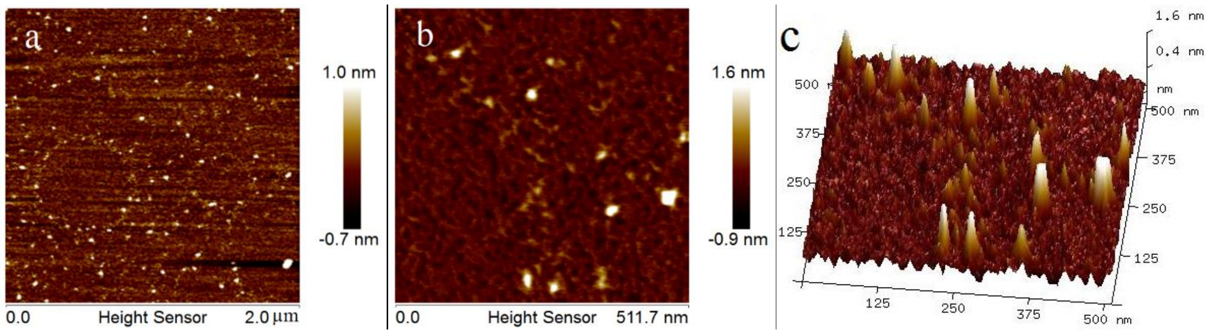

Supplementary Figure 49. AFM height (**a**,**b**) images and the 3-D plot feature (**c**) of *c*-[**PBNP**$_{10}$-(*b*-**PTNP**$_{160}$)$_2$-*b*-**PBNP**$_{10}$].

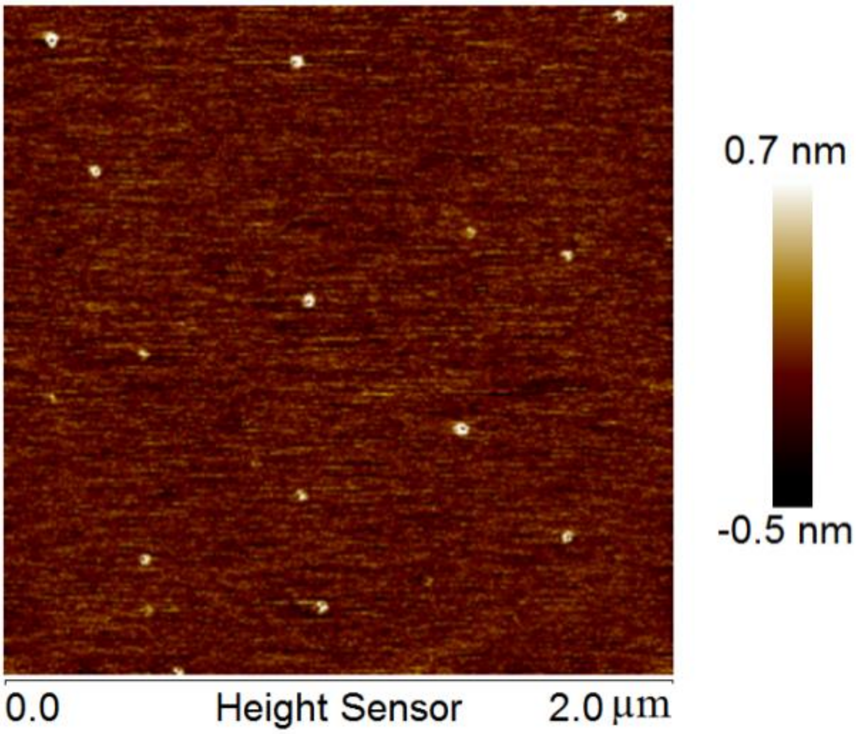

Supplementary Figure 50. AFM height image of *c*-[**PBNP**$_{10}$-(*b*-**PTNP**$_{160}$)$_2$-*b*-**PBNP**$_{10}$]-**POSS**.

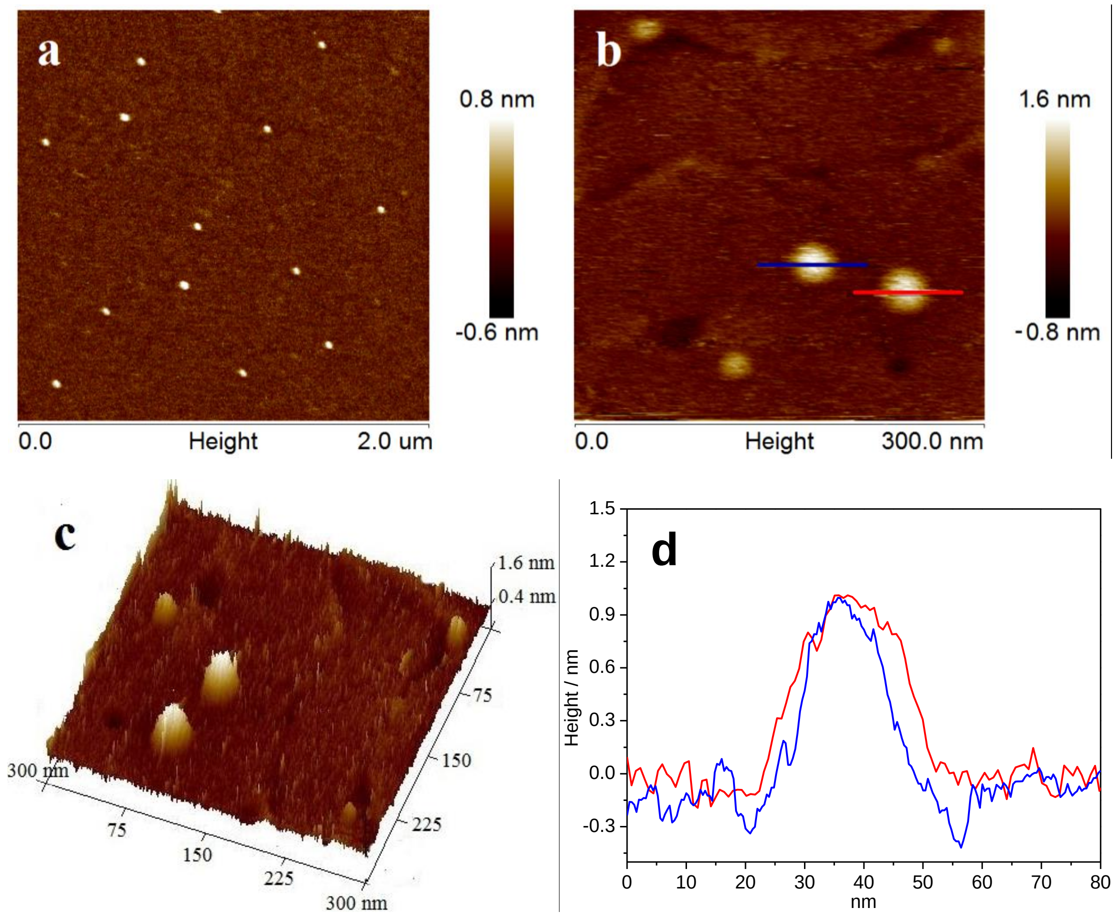

Supplementary Figure 51. AFM height images (**a**-**c**) of *l*-**PBNP**$_{20}$-(*b*-**PTNP**$_{160}$)$_2$-**POSS**. **d** Profile analysis of polymer marked by the red and blue lines in (**b**).

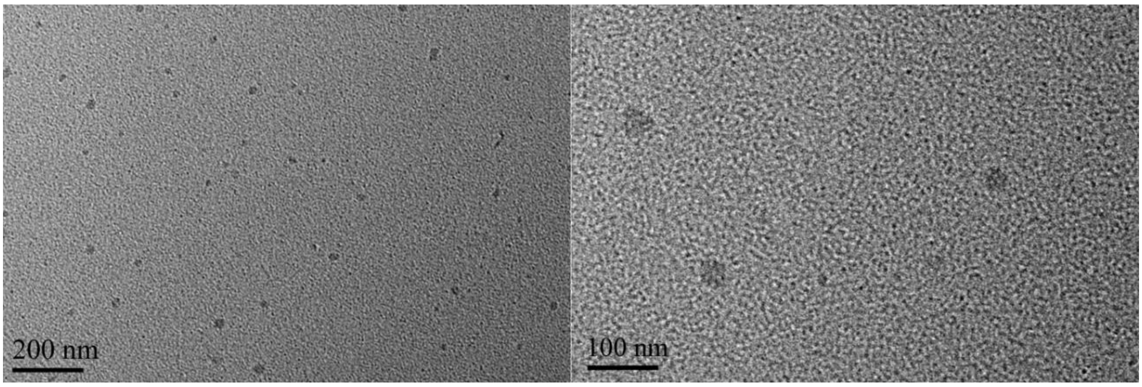

Supplementary Figure 52. TEM images of *c*-[**PBNP**$_{10}$-(*b*-**PTNP**$_{160}$)$_2$-*b*-**PBNP**$_{10}$] in $CHCl_3$ at 0.005 mg mL^{-1}.

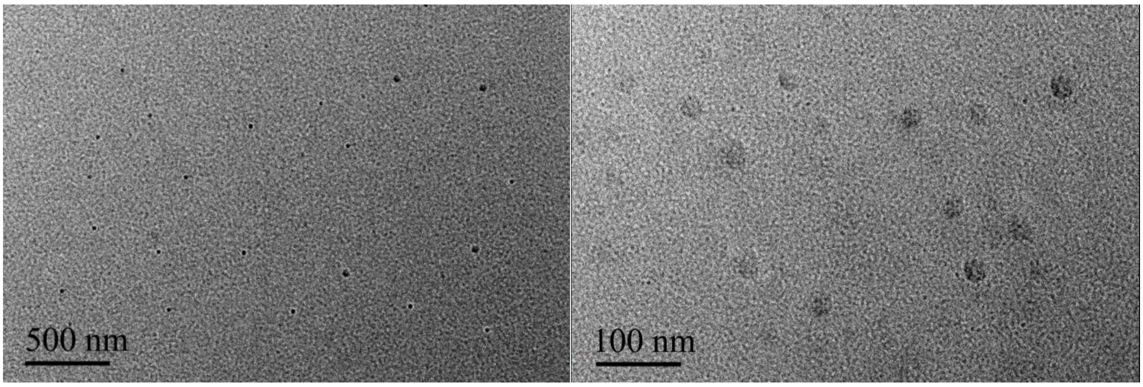

Supplementary Figure 53. TEM images of *c*-[**PBNP**$_{10}$-(*b*-**PNP**$_{80}$)$_2$-*b*-**PBNP**$_{10}$] in $CHCl_3$ at 0.005 mg mL^{-1}.

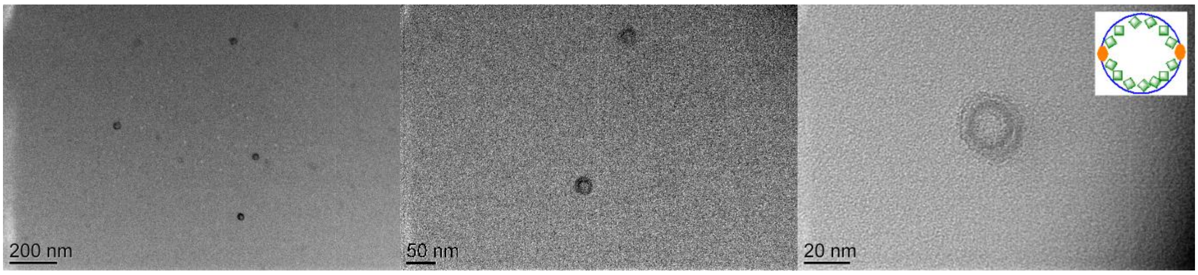

Supplementary Figure 54. TEM images of *c*-[**PBNP**$_{10}$-(*b*-**PTNP**$_{160}$)$_2$-*b*-**PBNP**$_{10}$]-**POSS** in $CHCl_3$ at 0.001 mg mL^{-1}.

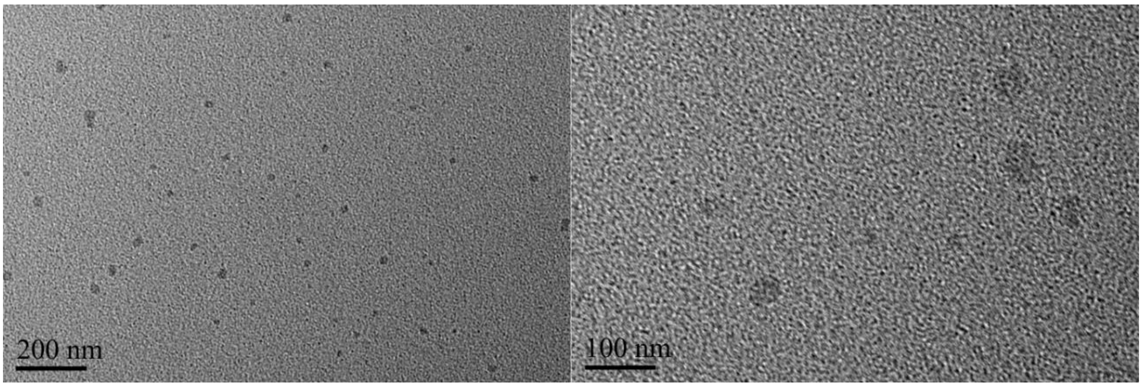

Supplementary Figure 55. TEM image of *l*-**PBNP**$_{20}$-(*b*-**PTNP**$_{160}$)$_2$ in $CHCl_3$ at 0.005 mg mL^{-1}.

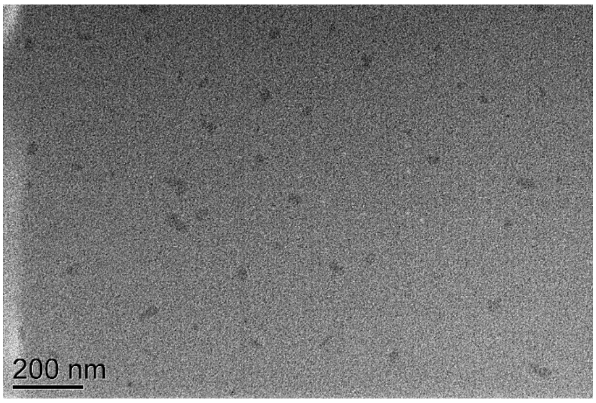

Supplementary Figure 56. TEM image of *l*-**PBNP**$_{20}$-(*b*-**PTNP**$_{160}$)$_2$-**POSS** in $CHCl_3$ at 0.005 mg mL^{-1}.

Supplementary Note 9. Dielectric feature of linear and cyclic polymers

The frequency dependence of the dielectric loss ($\varepsilon''_{M\mathrm{w}}(\omega)$) for two linear copolymers and the corresponding cyclic polymers with different M_ws at room temperature was exhibited in Supplementary Fig. 57a,b. The $\varepsilon''_{M\mathrm{w}}(\omega)$ mostly came from the local group motion responsible for the β-relaxation when the measured temperature was much lower than T_g.[26] The $\varepsilon''_{M\mathrm{w}}(\omega)$ was composed of the dielectric loss of end-groups ($\varepsilon''_{\mathrm{EG}}(\omega)$) and side-groups ($\varepsilon''_{M\mathrm{w}\to\infty}(\omega)$).[27,28] For the representative *l*-**PBNP**$_{20}$-(*b*-**PTNP**$_{20}$)$_2$ and *l*-**PBNP**$_{20}$-(*b*-**PTNP**$_{160}$)$_2$, as M_w increased, the peak position was moved from 2 Hz to 1 Hz (Supplementary Fig. 57a), and the intensity of dielectric loss decreased from 0.147 to 0.112 accompanying with the narrower loss peak, which was undoubtedly ascribed to the weakened end-group motion, because chain ends became more diluted and the $\varepsilon''_{\mathrm{EG}}(\omega)$ was mitigated.[27,29] However, the peak positions for *c*-[**PBNP**$_{10}$-(*b*-**PTNP**$_{20}$)$_2$-*b*-**PBNP**$_{10}$] and *c*-[**PBNP**$_{10}$-(*b*-**PTNP**$_{160}$)$_2$-*b*-**PBNP**$_{10}$] were in the vicinity of 1 Hz (Supplementary Fig. 57b), and the intensity of $\varepsilon''_{M\mathrm{w}}(\omega)$ for the high M_w cyclic polymer was slightly greater than that for the low M_w cyclic polymer (0.115 *vs* 0.122), because increasing the macroring size could offset the part of topological constrains, and the local group motion intensified.[27]

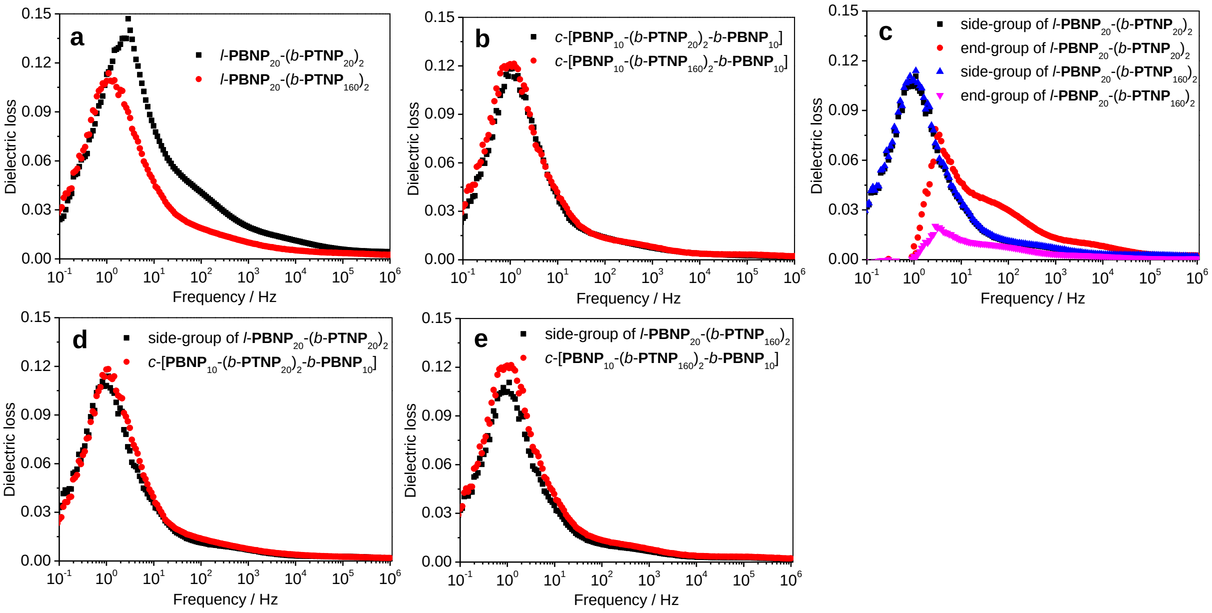

Supplementary Figure 57. Frequency dependence of dielectric loss. **a**,**b** $\varepsilon''_{M\mathrm{w}}(\omega)$ for the β-relaxation of representative linear (**a**) and cyclic (**b**) copolymers with different molecular weights at room temperature. **c** The resolved contributions originating from $\varepsilon''_{\mathrm{EG}}(\omega)$ and $\varepsilon''_{M\mathrm{w}\to\infty}(\omega)$ of two linear copolymers with different molecular weights. **d**,**e** Plots of $\varepsilon''_{\mathrm{EG}}(\omega)$ overlaying the $\varepsilon''_{M\mathrm{w}}(\omega)$ of cyclic polymers.

The individual plots of $\varepsilon''_{\mathrm{EG}}(\omega)$ and $\varepsilon''_{M\mathrm{w}\to\infty}(\omega)$ were deduced according to Supplementary equation (1):[29]

$$\varepsilon''_{M_w}(\omega) = \frac{2m_{EG}}{M_w}\varepsilon''_{\mathrm{EG}}(\omega) + (1 - \frac{2m_{EG}}{M_w})\varepsilon''_{M_{w\to\infty}}(\omega) \qquad (1)$$

where m_{EG} was the mass of end-group dipole ($m_{\mathrm{EG}} \approx m_{\mathbf{TNP}}$ = 429 g mol^{-1}). Then, to calculate the $\varepsilon''_{\mathrm{EG}}(\omega)$ and $\varepsilon''_{M\mathrm{w}\to\infty}(\omega)$, the $\varepsilon''_{M\mathrm{w}}(\omega)$ values of two linear copolymers with different M_{w}s were used, and the resulting individual plots were shown in Supplementary Fig. 57c. It was observed that the β-relaxation of end-group occurred mainly in higher frequencies in compartment in the side-group, and the peak positions of side-group and end-group were at 1 Hz and 3 Hz, respectively. Interestingly, the loss peak positions of cyclic polymers and side-groups of linear counterparts were in the same frequency (~ 1 Hz) (Supplementary Fig. 57d,e), and they could almost overlap, because the $\varepsilon''_{M\mathrm{w}}(\omega)$ of cyclic polymer came from $\varepsilon''_{M\mathrm{w}\to\infty}(\omega)$ due to the endless molecular topology, which proved that the cyclic structure was reliable and the cyclization of linear counterpart was successful. In addition, the peak intensity of cyclic polymer was stronger than the side-group of linear counterpart with the same repeat units, indicating that the macroring topology was more effective in suppressing local group motion in comparison to the linear macromolecule due to the intra-chain entanglement junctions. In brief, these observed discrepancies between linear and cyclic polymers were in well agreement with those of hydrodynamic volume, UV-vis absorption, fluorescence emission, and T_{g} values.

Supplementary References

1. Chen, J., Zhou, D., Wang, C., Liao, X., Xie, M. & Sun, R. High-performance dielectric ionic ladderphane-derived triblock copolymer with a unique self-assembled nanostructure. *RSC Adv.* **6**, 88874-88885 (2016).
2. You, Z., Song, W., Zhang, S., Jin, O. & Xie, M. Polymeric microstructures and dielectric properties of polynorbornenes with 3,5-bis(trifluoromethyl)biphenyl side groups by ring-opening metathesis polymerization. *J. Polym. Sci. Part A: Polym. Chem.* **51**, 4786-4798 (2013).
3. Love, J. A., Morgan, J. P., Trnka, T. M. & Grubbs, R. H. A practical and highly active ruthenium-based catalyst that effects the cross metathesis of acrylonitrile. *Angew. Chem. Int. Ed.* **41**, 4035-4037 (2002).
4. Choi, T. L. & Grubbs, R. H. Controlled living ring-opening metathesis polymerization by a fast-initiating ruthenium catalyst. *Angew. Chem. Int. Ed.* **115**, 1785-1788 (2003).
5. You, Z., Song, W., Zhang, S., Jin, O. & Xie, M. Polymeric microstructures and dielectric properties of polynorbornenes with 3,5-bis(trifluoromethyl)biphenyl side groups by ring-opening metathesis polymerization. *J. Polym. Sci. Part A: Polym. Chem.* **51**, 4786-4798 (2013).
6. You, Z., Gao, D., Jin, O., He, X. & Xie, M. High dielectric performance of tactic polynorbornene derivatives synthesized by ring-opening metathesis polymerization. *J. Polym. Sci. Part A: Polym. Chem.* **51**, 1292-1301 (2013).

7. Chou, C. M., Lee, S. L., Chen, C. H., Biju, A. T., Wang, H. W., Wu, Y. L., Zhang, G. F., Yang, K. W., Lim, T. S., Huang, M. J., Tsai, P. Y., Lin, K. C., Huang, S. L., Chen, C. h. & Luh, T. Y. Polymeric ladderphanes. *J. Am. Chem. Soc.* **131**, 12579-12585 (2009).
8. Yang, H. C., Lin, S. Y., Yang, H. C., Lin, C. L., Tsai, L., Huang, S. L., Chen, I. P., Chen, C. H., Jin, B. Y. and & Luh, T. Y. Molecular architecture towards helical double-stranded polymers. *Angew. Chem. Int. Ed.* **45**: 726-730 (2006).
9. Yeh, N. H., Chen, C. W., Lee, S. L., Wu, H. J., Chen, C. h. & Luh T. Y. Polynorbornene-based double-stranded ladderphanes with cubane, cuneane, tricyclooctadiene, and cyclooctatetraene linkers. *Macromolecules* **45**, 2662-2667 (2012).
10. Sun, Y. & Li, Z. In situ polymerization of supramolecular nanorods assembled from polymerizable perylene bisimide. *Polym. Chem.* **8**, 4422-4427 (2017).
11. Zhu, L., Lin, N. T., Xie, Z. Y., Lee, S. L., Huang, S. L., Yang, J. H., Lee, Y. D., Chen, C. h., Chen, C. H. & Luh, T. Y. Ruthenium-catalyzed cascade metathetical cyclopolymerization of bisnorbornenes with flexible linkers. *Macromolecules* **46**, 656-663 (2013).
12. Isono, T., Sasamori, T., Honda, K., Mato, Y., Yamamoto, T., Tajima, K. & Satoh, T. Multicyclic polymer synthesis through controlled/living cyclopolymerization of α,ω-dinorbornenyl-functionalized macromonomers. *Macromolecules* **51**, 3855-3864 (2018).
13. Roland, C., Li, H., Abboud, K., Wagener, K. & Veige, A. Cyclic polymers from alkynes. *Nat. Chem.* **8**, 791-796 (2016).
14. Zhu, X, Zhou, N., Zhang, Z., Sun, B., Yang, Y., Zhu, J. & Zhu, X. A. Cyclic polymers with pendent carbazole units: enhanced fluorescence and redox behavior. *Angew. Chem. Int. Ed.* **50**, 6615-6618 (2011).
15. Bielawski, C. W., Benitez, D. & Grubbs, R. H. An “endless” route to cyclic polymers. *Science* **297**, 2041-2044 (2002).
16. Cai, Y., Lu, J., Zhou, F., Zhou, X., Zhou, N., Zhang, Z. & Zhu, X. Cyclic amphiphilic random copolymers bearing azobenzene side chains: facile synthesis and topological effects on self-assembly and photoisomerization. *Macromol. Rapid Commun.* **35**, 901-907 (2014).
17. Laurent, B. A. & Grayson, S. M. Synthetic approaches for the preparation of cyclic polymers. *Chem. Soc. Rev.* **38**, 2202-2213 (2009).
18. Luh, T. Y. Ladderphanes: a new type of duplex polymers. *Accounts. Chem. Res.* **46**, 378-389 (2013).
19. Zhao, K., Khan, H. U., Li, R., Su, Y. & Amassian, A. Entanglement of conjugated polymer chains influences molecular self-assembly and carrier transport. *Adv. Funct. Mater.* **23**, 6024-6035 (2013).
20. Lee. J., Baek. K., Kim. M., Yun. G., Ko, Y. H., Lee, N. S., Hwang, I., Kim, J., Natarajan, R., Park, C., Sung, W. & Kim, K. Hollow nanotubular toroidal polymer microrings. *Nat. Chem.* **6**, 97-103 (2014).
21. Li, J., Zhou, N., Zhang, Z., Xu, Y., Chen, X., Tu, Y., Hu, Z. & Zhu, X. L. A smart cyclic azobenzene as pendant groups on polymer chains: topological effect of the cyclization on thermal and photoresponsive properties of the azobenzene and the polymer. *Chem. Asian. J.* **8**, 1095-1100 (2013).
22. Lai. C. T. & Hong. J. L. Influence of molecular weight on the aggregation-induced emission

enhancement and spectral stability of vinyl polymers containing the fluorescent 2,4,6-triphenylpyridine pendant groups. *J. Mater. Chem.* **22**, 9546 (2012).

23. Kwok, R. T., Leung, C. W., Lam, J. W. & Tang, B. Z. Biosensing by luminogens with aggregation-induced emission characteristics. *Chem. Soc. Rev.* **44**, 4228-4238 (2015).
24. Sanji, T., Nakamura, M., Kawamata, S., Tanaka, M., Itagaki, S. & Gunji, T. Fluorescence "turn-on" detection of melamine with aggregation-induced-emission-active tetraphenylethene. *Chem. Eur. J.* **18**, 15254-15257 (2012).
25. Noguchi, T., Roy, B., Yoshihara, D., Tsuchiya, Y., Yamamoto, T. & Shinkai, S. Cyclization-induced turn-on fluorescence system applicable to dicarboxylate sensing. *Chem. Eur. J.* **20**, 381-384 (2014).
26. Wei, J., Zhang, Z., Tseng, J. K., Treufeld, I., Liu, X., Litt, M. H. & Zhu, L. Achieving high dielectric constant and low loss property in a dipolar glass polymer containing strongly dipolar and small-sized sulfone groups. *ACS appl. Mater. Inter.* **7**, 5248-5257 (2015).
27. Gambino, T., Martínez de Ilarduya, A., Alegría, A. & Barroso-Bujans, F. Dielectric relaxations in poly(glycidyl phenyl ether): effects of microstructure and cyclic topology. *Macromolecules* **49**, 1060-1069 (2016).
28. Ochs, J., Veloso, A., Martínez-Tong, D. E., Alegria, A. & Barroso-Bujans, F. An insight into the anionic ring-opening polymerization with tetrabutylammonium azide for the ceneration of pure cyclic poly(glycidyl phenyl ether). *Macromolecules* **51**, 2447-2455 (2018).
29. Yamane, M., Hirose, Y. & Adachi, K. Dielectric study of terminal chain dynamics, segmental motion, and rotation of side groups in polyethers of type ABC. *Macromolecules* **38**, 10686-10693 (2005).
